# Supplementary material for: Genome-wide characterization of the auxin response factor (ARF) gene family of litchi (Litchi chinensis Sonn.): phylogenetic analysis, miRNA regulation and expression changes during fruit abscission
Source: PeerJ. 2019 Apr 4;7:e6677. doi: 10.7717/peerj.6677 (PMC6451834; doi:10.7717/peerj.6677)
Supplement: Supplemental Information 3 — YF, young fruit (25 days after fertilization); FF, female flower; MF, male flower; USF, undeterminated sex flowers; ML, mature leaves; YL, young leaves; FBS, fruit-bearing shoots. The red font represents the genes in group1, group2, and group3 in Fig. 5. [file peerj-07-6677-s003.docx]

Table S3 Potential functions of LcARFs

| Genes | Tissues | Functions in other studies | References |
| --- | --- | --- | --- |
| *LcARF1A* | ML/USF/FF | leaf senescence and flower abscission | (Ellis et al., 2005) |
| *LcARF1B* | ML/FBS/USF |  |  |
| *LcARF1C* | ML/FBS/USF/FF |  |  |
| *LcARF1D* | ML/USF/MF/FF |  |  |
| *LcARF2A* | ML/USF/FF/YF | leaf senescence and flower abscission, fruit ripening, female and male gametophyte development, leaf longevity, lateral root formation and flower organ senescence | (Ellis et al., 2005; Lim et al., 2010; Breitel et al., 2016; Liu et al., 2017; Ren et al., 2017) |
| *LcARF2B* | ML/USF/FF |  |  |
| *LcARF2C* | ML/USF/YF |  |  |
| *LcARF2D* | ML/USF/FF |  |  |
| *LcARF2E* | ML/USF/FF |  |  |
| *LcARF2F* | USF/MF/FF/YF |  |  |
| *LcARF3A* | ML/FBS/USF/FF | leaf polarity specification, floral meristem determinacy, lateral organs, formation of epidermal cells and trichomes, de novo shoot regeneration, female and male gametophyte development, early flower development, developmental timing and patterning, somatic embryogenesis integument development and polarity determination | (Fahlgren et al., 2006; Finet et al., 2010; Kelley et al., 2012; Liu et al., 2014a; Zhang et al., 2018; Pekker, 2005; Zheng et al., 2018; Liu et al., 2017; Cheng et al., 2013; Lin et al., 2015b) |
| *LcARF3B* | ML/USF/FF |  |  |
| *LcARF4A* | ML/FBS/USF/FF | organ polarity, lateral organs, sugar metabolism during tomato fruit development, female and male gametophyte development | (Hunter, 2006; Finet et al., 2010; Sagar et al., 2013; Liu et al., 2017; Yifhar et al., 2012; Pekker, 2005; Lin et al., 2015b) |
| *LcARF4B* | ML/FBS/USF/YF |  |  |
| *LcARF4C* | ML/FBS/USF/YF |  |  |
| *LcARF5A* | FBS/USF/MF/FF | embryonic root and flower formation, embryo patterning and vasculature, female and male gametophyte development | (Hardtke & Berleth, 1998; Liu et al., 2017) |
| *LcARF5B* | FBS/USF/MF/FF |  |  |
| *LcARF6A* | FBS/USF/MF/FF | flower maturation, floral development and female sterility, stress response | (Nagpal, 2005; Wu, Tian & Reed, 2006; Liu et al., 2014b; Jodder et al., 2017) |
| *LcARF6B* | FBS/USF/MF/FF |  |  |
| *LcARF6C* | ML/FBS/USF/MF/FF |  |  |
| *LcARF6D* | ML/FBS/USF/MF/FF |  |  |
| *LcARF7A* | ML/FBS/USF | embryo patterning and vasculature, leaf expansion and lateral root growth, fruit set fruit growth in tomatoes, cross-talk between auxin and gibberellin signaling during tomato fruit set and development | (Hardtke & Berleth, 1998; Wilmoth et al., 2005; De Jong et al., 2009, 2011) |
| *LcARF7B* | ML/FBS/USF |  |  |
| *LcARF8A* | FBS/USF/MF/FF | fertilization and fruit development, flower maturation, floral development and female sterility, nodulation and lateral root development, bacterial stress, stamen elongation and endothecium lignification, parthenocarpy | (Goetz, 2006; Nagpal, 2005; Liu et al., 2014b; Jodder et al., 2017; Goetz et al., 2007; Wang et al., 2015; Ghelli et al., 2018) |
| *LcARF8B* | ML/FBS/USF/MF/FF |  |  |
| *LcARF9A* | ML/USF/MF/FF | cell division during early tomato fruit development | (De Jong et al., 2015) |
| *LcARF9B* | ML/USF/MF/FF |  |  |
| *LcARF10A* | ML/MF | lateral root formation, ovary patterning, floral organ abscission and lamina outgrowth, seed germination and post-germination stages, somatic embryogenesis | (Wang, 2005; Damodharan, Zhao & Arazi, 2016; Liu et al., 2007; Lin et al., 2015a) |
| *LcARF10B* | ML/FBS/USF/MF |  |  |
| *LcARF16A* | ML/MF | lateral root formation, phosphate stress, ovary patterning, floral organ abscission and lamina outgrowth, somatic embryogenesis | (Wang, 2005; Damodharan, Zhao & Arazi, 2016; Shen et al., 2013; Shen et al., 2014; Lin et al., 2015a) |
| *LcARF16B* | ML/USF |  |  |
| *LcARF16C* | ML/USF/FF |  |  |
| *LcARF16D* | ML/USF/MF/FF |  |  |
| *LcARF17A* | ML/FBS/USF/MF/FF | primexine formation in pollen, ovary patterning, floral organ abscission and lamina outgrowth, somatic embryogenesis | (Yang et al., 2013; Damodharan, Zhao & Arazi, 2016; Lin et al., 2015a) |
| *LcARF17B* | ML/FBS/USF/MF/YF |  |  |
| *LcARF18A* | ML/USF | seed weight and silique length | (Liu et al., 2015) |
| *LcARF18B* | YL/ML/USF |  |  |
| *LcARF19A* | ML | leaf expansion and lateral root growth, rice leaf angles | (Wilmoth et al., 2005; Zhang et al., 2015) |
| *LcARF19B* | USF/MF/FF |  |  |

YF: young fruit (25 days after fertilization), FF: female flower, MF: male flower, USF: undeterminated sex flowers, ML:mature leaves, YL: young leaves, FBS: fruit-bearing shoots.

The red font represents the genes in group1, group2 and group3 in Figure 5.

References:

Breitel DA, Chappell-Maor L, Meir S, Panizel I, Puig CP, Hao Y, Yifhar T, Yasuor H, Zouine M, Bouzayen M, Granell Richart A, Rogachev I, Aharoni A. 2016. AUXIN RESPONSE FACTOR 2 Intersects Hormonal Signals in the Regulation of Tomato Fruit Ripening. *PLoS Genetics* 12:1–34. DOI: 10.1371/journal.pgen.1005903.

Cheng ZJ, Wang L, Sun W, Zhang Y, Zhou C, Su YH, Li W, Sun TT, Zhao XY, Li XG, Cheng Y, Zhao Y, Xie Q, Zhang XS. 2013. Pattern of Auxin and Cytokinin Responses for Shoot Meristem Induction Results from the Regulation of Cytokinin Biosynthesis by AUXIN RESPONSE FACTOR3. *Plant Physiology* 161:240–251. DOI: 10.1104/pp.112.203166.

Damodharan S, Zhao D, Arazi T. 2016. A common miRNA160-based mechanism regulates ovary patterning, floral organ abscission and lamina outgrowth in tomato. *Plant Journal* 86:458–471. DOI: 10.1111/tpj.13127.

Ellis CM, Nagpal P, Young JC, Hagen G, Guilfoyle TJ, Reed JW. 2005. AUXIN RESPONSE FACTOR1 and AUXIN RESPONSE FACTOR2regulate senescence and floral organ abscission in Arabidopsisthaliana. *Development* 132:4563–4574. DOI: 10.1242/dev.02012.

Fahlgren N, Montgomery TA, Howell MD, Allen E, Dvorak SK, Alexander AL, Carrington JC. 2006. Regulation of AUXIN RESPONSE FACTOR3 by TAS3 ta-siRNA Affects Developmental Timing and Patterning in Arabidopsis. *Current Biology* 16:939–944. DOI: 10.1016/j.cub.2006.03.065.

Finet C, Fourquin C, Vinauger M, Berne-Dedieu A, Chambrier P, Paindavoine S, Scutt CP. 2010. Parallel structural evolution of auxin response factors in the angiosperms. *Plant Journal* 63:952–959. DOI: 10.1111/j.1365-313X.2010.04292.x.

Ghelli R, Napoli N, Brunetti P, De Paolis A, Cecchetti V, Tsuge T, Serino G, Matsui M, Mele G, Rinaldi G, Palumbo GA, Barozzi F, Costantino P, Cardarelli M. 2018. A Newly Identified Flower-Specific Splice Variant of AUXIN RESPONSE FACTOR8 Regulates Stamen Elongation and Endothecium Lignification in Arabidopsis. *The Plant Cell* 30:tpc.00840.2017. DOI: 10.1105/tpc.17.00840.

Goetz M. 2006. AUXIN RESPONSE FACTOR8 Is a Negative Regulator of Fruit Initiation in Arabidopsis. *the Plant Cell Online* 18:1873–1886. DOI: 10.1105/tpc.105.037192.

Goetz M, Hooper LC, Johnson SD, Rodrigues JCM, Vivian-Smith A, Koltunow AM. 2007. Expression of Aberrant Forms of AUXIN RESPONSE FACTOR8 Stimulates Parthenocarpy in Arabidopsis and Tomato. *Plant Physiology* 145:351–366. DOI: 10.1104/pp.107.104174.

Hardtke CS, Berleth T. 1998. The *Arabidopsis* gene *MONOPTEROS* encodes a transcription factor mediating embryo axis formation and vascular development. *EMBO Journal* 17:1405–1411. DOI: 10.1093/emboj/17.5.1405.

Hunter C. 2006. Trans-acting siRNA-mediated repression of ETTIN and ARF4 regulates heteroblasty in Arabidopsis. *Development* 133:2973–2981. DOI: 10.1242/dev.02491.

Jodder J, Basak S, Das R, Kundu P. 2017. Coherent regulation of miR167a biogenesis and expression of auxin signaling pathway genes during bacterial stress in tomato. *Physiological and Molecular Plant Pathology* 100:97–105. DOI: 10.1016/j.pmpp.2017.08.001.

De Jong M, Wolters-Arts M, Feron R, Mariani C, Vriezen WH. 2009. The Solanum lycopersicum auxin response factor 7 (SlARF7) regulates auxin signaling during tomato fruit set and development. *Plant Journal* 57:160–170. DOI: 10.1111/j.1365-313X.2008.03671.x.

De Jong M, Wolters-Arts M, García-Martínez JL, Mariani C, Vriezen WH. 2011. The Solanum lycopersicum AUXIN RESPONSE FACTOR 7 (SlARF7) mediates cross-talk between auxin and gibberellin signalling during tomato fruit set and development. *Journal of Experimental Botany* 62:617–626. DOI: 10.1093/jxb/erq293.

De Jong M, Wolters-Arts M, Schimmel BCJ, Stultiens CLM, De Groot PFM, Powers SJ, Tikunov YM, Bovy AG, Mariani C, Vriezen WH, Rieu I. 2015. Solanum lycopersicum AUXIN RESPONSE FACTOR 9 regulates cell division activity during early tomato fruit development. *Journal of Experimental Botany* 66:3405–3416. DOI: 10.1093/jxb/erv152.

Kelley DR, Arreola A, Gallagher TL, Gasser CS. 2012. ETTIN (ARF3) physically interacts with KANADI proteins to form a functional complex essential for integument development and polarity determination in Arabidopsis. *Development* 139:1105–1109. DOI: 10.1242/dev.067918.

Lim PO, Lee IC, Kim J, Kim HJ, Ryu JS, Woo HR, Nam HG. 2010. Auxin response factor 2 (ARF2) plays a major role in regulating auxin-mediated leaf longevity. *Journal of Experimental Botany* 61:1419–1430. DOI: 10.1093/jxb/erq010.

Lin Y, Lai Z, Tian Q, Lin L, Lai R, Yang M, Zhang D, Chen Y, Zhang Z. 2015a. Endogenous target mimics down-regulate miR160 mediation of ARF10, -16, and -17 cleavage during somatic embryogenesis in Dimocarpus longan Lour. *Frontiers in Plant Science* 6:1–16. DOI: 10.3389/fpls.2015.00956.

Lin Y, Lin L, Lai R, Liu W, Chen Y, Zhang Z, XuHan X, Lai Z. 2015b. MicroRNA390-Directed TAS3 Cleavage Leads to the Production of tasiRNA-ARF3/4 During Somatic Embryogenesis in Dimocarpus longan Lour. *Frontiers in Plant Science* 6:1–15. DOI: 10.3389/fpls.2015.01119.

Liu X, Dinh TT, Li D, Shi B, Li Y, Cao X, Guo L, Pan Y, Jiao Y, Chen X. 2014a. AUXIN RESPONSE FACTOR 3 integrates the functions of AGAMOUS and APETALA2 in floral meristem determinacy. *Plant Journal* 80:629–641. DOI: 10.1111/tpj.12658.

Liu J, Hua W, Hu Z, Yang H, Zhang L, Li R, Deng L, Sun X, Wang X, Wang H. 2015. Natural variation in ARF18 gene simultaneously affects seed weight and silique length in polyploid rapeseed. *Proceedings of the National Academy of Sciences of the United States of America* 112:E5123-32. DOI: 10.1073/pnas.1502160112.

Liu Z, Miao L, Huo R, Song X, Johnson C, Kong L, Sundaresan V, Yu X. 2017. ARF2–ARF4 and ARF5 are Essential for Female and Male Gametophyte Development in Arabidopsis. *Plant and Cell Physiology* 59:179–189. DOI: 10.1093/pcp/pcx174.

Liu PP, Montgomery TA, Fahlgren N, Kasschau KD, Nonogaki H, Carrington JC. 2007. Repression of AUXIN RESPONSE FACTOR10 by microRNA160 is critical for seed germination and post-germination stages. *Plant Journal* 52:133–146. DOI: 10.1111/j.1365-313X.2007.03218.x.

Liu N, Wu S, Houten J Van, Wang Y, Ding B, Fei Z, Clarke TH, Reed JW, Van Der Knaap E. 2014b. Down-regulation of AUXIN RESPONSE FACTORS 6 and 8 by microRNA 167 leads to floral development defects and female sterility in tomato. *Journal of Experimental Botany* 65:2507–2520. DOI: 10.1093/jxb/eru141.

Nagpal P. 2005. Auxin response factors ARF6 and ARF8 promote jasmonic acid production and flower maturation. *Development* 132:4107–4118. DOI: 10.1242/dev.01955.

Pekker I. 2005. Auxin Response Factors Mediate Arabidopsis Organ Asymmetry via Modulation of KANADI Activity. *the Plant Cell Online* 17:2899–2910. DOI: 10.1105/tpc.105.034876.

Ren Z, Liu R, Gu W, Dong X. 2017. The Solanum lycopersicum auxin response factor SlARF2 participates in regulating lateral root formation and flower organ senescence. *Plant Science* 256:103–111. DOI: 10.1016/j.plantsci.2016.12.008.

Sagar M, Chervin C, Mila I, Hao Y, Roustan J-P, Benichou M, Gibon Y, Biais B, Maury P, Latche A, Pech J-C, Bouzayen M, Zouine M. 2013. SlARF4, an Auxin Response Factor Involved in the Control of Sugar Metabolism during Tomato Fruit Development. *Plant Physiology* 161:1362–1374. DOI: 10.1104/pp.113.213843.

Shen C, Wang S, Zhang S, Xu Y, Qian Q, Qi Y, Jiang DA. 2013. OsARF16, a transcription factor, is required for auxin and phosphate starvation response in rice (Oryza sativa L.). *Plant, Cell and Environment* 36:607–620. DOI: 10.1111/pce.12001.

Shen C, Yue R, Yang Y, Zhang L, Sun T, Tie S, Wang H. 2014. OsARF16 is involved in cytokinin-mediated inhibition of phosphate transport and phosphate signaling in rice (Oryza sativa L). *PLoS ONE* 9:3–12. DOI: 10.1371/journal.pone.0112906.

Wang J-W. 2005. Control of Root Cap Formation by MicroRNA-Targeted Auxin Response Factors in Arabidopsis. *the Plant Cell Online* 17:2204–2216. DOI: 10.1105/tpc.105.033076.

Wang Y, Li K, Chen L, Zou Y, Liu H, Tian Y, Li D, Wang R, Zhao F, Ferguson BJ, Gresshoff PM, Li X. 2015. MicroRNA167-Directed Regulation of the Auxin Response Factors *GmARF8a* and *GmARF8b* Is Required for Soybean Nodulation and Lateral Root Development. *Plant Physiology* 168:984–999. DOI: 10.1104/pp.15.00265.

Wilmoth JC, Wang S, Tiwari SB, Joshi AD, Hagen G, Guilfoyle TJ, Alonso JM, Ecker JR, Reed JW. 2005. NPH4/ARF7 and ARF19 promote leaf expansion and auxin-induced lateral root formation. *Plant Journal* 43:118–130. DOI: 10.1111/j.1365-313X.2005.02432.x.

Wu M-F, Tian Q, Reed JW. 2006. Arabidopsis microRNA167 controls patterns of ARF6 and ARF8 expression, and regulates both female and male reproduction. *Development* 133:4211–4218. DOI: 10.1242/dev.02602.

Yang J, Tian L, Sun M-X, Huang X-Y, Zhu J, Guan Y-F, Jia Q-S, Yang Z-N. 2013. AUXIN RESPONSE FACTOR17 Is Essential for Pollen Wall Pattern Formation in Arabidopsis. *Plant Physiology* 162:720–731. DOI: 10.1104/pp.113.214940.

Yifhar T, Pekker I, Peled D, Friedlander G, Pistunov A, Sabban M, Wachsman G, Alvarez JP, Amsellem Z, Eshed Y. 2012. Failure of the Tomato Trans-Acting Short Interfering RNA Program to Regulate AUXIN RESPONSE FACTOR3 and ARF4 Underlies the Wiry Leaf Syndrome. *The Plant Cell* 24:3575–3589. DOI: 10.1105/tpc.112.100222.

Zhang S, Wang S, Xu Y, Yu C, Shen C, Qian Q, Geisler M, Jiang DA, Qi Y. 2015. The auxin response factor, OsARF19, controls rice leaf angles through positively regulating OsGH3-5 and OsBRI1. *Plant, Cell and Environment* 38:638–654. DOI: 10.1111/pce.12397.

Zhang K, Wang R, Zi H, Li Y, Cao X, Li D, Guo L, Tong J, Pan Y, Jiao Y, Liu R, Xiao L, Liu X. 2018. AUXIN RESPONSE FACTOR3 Regulates Floral Meristem Determinacy By Repressing Cytokinin Biosynthesis and Signaling. *The Plant Cell* 30:tpc.00705.2017. DOI: 10.1105/tpc.17.00705.

Zheng Y, Zhang K, Guo L, Liu X, Zhang Z. 2018. AUXIN RESPONSE FACTOR3 plays distinct role during early flower development. *Plant Signaling and Behavior* 13:1–4. DOI: 10.1080/15592324.2018.1467690.
