# Supplementary material for: Genome-wide characterization of the auxin response factor (ARF) gene family of litchi (Litchi chinensis Sonn.): phylogenetic analysis, miRNA regulation and expression changes during fruit abscission
Source: PeerJ. 2019 Apr 4;7:e6677. doi: 10.7717/peerj.6677 (PMC6451834; doi:10.7717/peerj.6677)
Supplement: Supplemental Information 4 [file peerj-07-6677-s004.docx]

Supplemental Data S1_Amino acid sequences of 39 LcARFs

>LcARF6A

MRLSSAGFSPQTQEGEKRVLNSELWHACAGPLVSLPAVGSRVVYFPQGHSEQVAASTNKEVDAHIPNYPSLPPQLICQLHNVTMHADIETDEVYAQMTLQPLNPQEQKDAYLPAELGNPSKQPTNYFCKTLTASDTSTHGGFSVPRRAAEKVFPPLDFTQQPPAQELIARDLHDNEWKFRHIFRGQPKRHLLTTGWSVFVSAKRLIAGDSVLFIWNEKNQLLLGIRRANRPPTVMPSSVLSSDSMHLGLLAAAAHAAATNSRFTIFYNPRASPSEFVIPLAKYVKAVYHTRVSVGMRFRMLFETEESSVRRYMGTITGISDLDPVRWPNSHWRSVKVGWDESTAGERQPRVSLWEIEPLTTFPMYPSPFPLRLKRPWPPGLPAFHGIKEEDMGLNSQLMWLRGDGDRGIQSLNFQGLGVTPWMQPRLDASMLGLQTDMYQAMAAAALREMRAVDTTKPAHTSLLQFQQPQNLPSRSAALMQQQMLQQSHPQQTFLQGVQENQHQPHSQAQTQSHLLQPQLQHPQSFPNQQHQLPQPQQQQVDHQQVPTAVSAMPQFTSVSQSQSPPMQAVSSLCHQQSFSDSNGNTVTSPIVSPLHSLLGSFSQDESSHLINLPRSNPLIPSPTWPSKRVAVESLFPSGGPQCVLPQVEQLGPPQTNVSQNSISLPPFPGRECSIDQEGNADPHSHLLFGVNIEPPSLLMQNEMSSLGGVGSNSDSTTIPFASNYMSTAGTDFSVNPAITATSCIDESGFLQSPENVGQGNPPTRTFVKVHKSGSFGRSLDITKFSSYHELRGELARMFGLEGQLEDPLRSGWQLVFVDRENDVLLLGDDPWPEFVNSVWCIKILSPPEVQQMGKRGIELLNKVPIPRLSNNGCDDYASRQDSRNLSSGITSVGSLDY

>LcARF6B

MRPFSAGFSPQTQEGEKRVLNSELWHACAGPLVSLPAVGSRVVYFPQGHSEQVAASTNKEVDAHIPNYPSLPPQLICQLHNVTMHADIETDEVYAQMTLQPLNPQEQKDAYLPAELGNPSKQPTNYFCKTLTASDTSTHGGFSVPRRAAEKVFPPLDFTQQPPAQELIARDLHDNEWKFRHIFRGQPKRHLLTTGWSVFVSAKRLIAGDSVLFIWNEKNQLLLGIRRANRPPTVMPSSVLSSDSMHLGLLAAAAHAAATNSRFTIFYNPRASPSEFVIPLAKYVKAVYHTRVSVGMRFRMLFETEESSVRRYMGTITGISDLDPVRWPNSHWRSVKVGWDESTAGERQPRVSLWEIEPLTTFPMYPSPFPLRLKRPWPPGLPAFHGIKEEDMGLNSQLMWLRGDGDRGIQSLNFQGLGVTPWMQPRLDASMLGLQTDMYQAMAAAALREMRAVDTTKPAHTSLLQFQQPQNLPSRSAALMQQQMLQQSHPQQTFLQGVQENQHQPHSQAQTQSHLLQAQLQHPQSFPNQQHQLPQQQQQVDHQQVPTAVSAMPQFTSVSQSQSPPMQAVSSLCHQQSFSDSNGNTVTSPIVSPLHSLLGSFSQDESSHLINLPRSNPLIPSPTWPSKRVAVESLFPSGGPQCVLPQVEQLGPPQTNVSQNSISLPPFPGRECSIDQEGNADPHSHLLFGVNIDPPSILMQNEMSSLGGVGNNSDSTTMPFASNYMSTAGTDFSVNPAITPTSCIDESCFLQSPENVGQGNPPTRTFVKVHKSGSFGRSLDITKFSSYHELRSELARMFGLEGQLEDPLRSGWQLVFVDRENDVLLLGDDPWPEFVNSVWCIKILSPPEVQQMGKRGIELLNKVPIPRLSNNGCDDYASRQDSRNLSSGITSVGSLDY

>LcARF6C

MRLKTCVPPAPKPKVKTYDKLMTCELTSFELAPPQLVKTTVDNLGEKKCLNSELWHACAGPLVSLPPVGSRVVYFPQGHSEQVAASTNKEVDAHIPNYPSLPPQLICQLHNVTMHADVETDEVYAQMTLQPLSPQEQKDVYLLPAELGTGNKQPTNYFCKTLTASDTSTHGGFSVPRRAAEKVFPPLDYSLTPPAQELIARDLHDNEWKFRHIFRGQPKRHLLTTGWSVFVSAKRLVAGDSVLFIWNDKNQLLLGIRRANRPQTVMPSSVLSSDSMHIGLLAAAAHAAATNSRFTIFYNPRASPSEFVIPLAKYVKAVYHTRVSVGMRFRMLFETEESSVRRYMGTITGISDLDPVRWPNSHWRSVKVGWDESTAGERQPRVSLWEIEPLTTFPMYPSPFPLRLKRPWPSGLPSFPVVKDGDMSINSSLMWLQGGVGDQGIQSLNFQGFGVSPWMQPRLDTSMPGLQPDVYQAMAAAALQEMRTVDSSKLASQSLLQFQQSQSASNGPASLIPRQMLQQSHPQNAYVQSFQDNQASAQAQLLQQQLQRQHSYNDQRQQQQQVQQPQQMPQLSVQPQIPNIISALPHLASTSQSQPPTLQAIAPQCQQPSFSDSLGNPIATSDVSSVHSILGSLSQDGSSHLLSSNVSNPIITSSSIITKQVSVDPHLSSGVSHCVLPQVEQFGTQQSNVSELANLLPPFPGREYSSYQGSADPQNNLLFGVSIDSSSLMAQHGLQNLKNIGSENDSLSLPFAASNFTSAVGTDFPLNSEMTTSSCVDESGFLPSSENVEQVNTSNRTFVKIPNIISALPHLASTSQSQPATLQAIAPQCQQPSFSDSLGNPMVTSDVSSVHSILGSLSQDGSSHLLSSNVSNPIITSSSIITKQVSVDPHLSSGVSHCVLPQVEQFGTQQSNVSELANLLPPFPGREYSSYQGTADPQNNLLFGVSIDSSSLMAQHGLQNLKNIGSENDSLSLPFAASNFTSAVGTDFPLNSEMTTSSCVDESGFLPSSENVEQVNTSNRTFVKVHKSGSFGRSLDISKFSSYDELRSELARLFGLEGQLEDPQRSGWQLVFVDRENDVLLLGDDPWQEFVNNVWYIKILSPLEVQQMGKEGLSSMSSVPGQRLSNNNCDDYLSRQELRSSSNGVASMGSLDY

>LcARF6D

MRIKWRDGAHHKEKEGASCDLRGREKKCLNSELWHACAGPLVSLPPVGSRVVYFPQGHSEQVAASTNKEVDAHIPNYPSLPPQLICQLHNVTMHADVETDEVYAQMTLQPLSPQEQKDVYLLPAELGTGNKQPTNYFCKTLTASDTSTHGGFSVPRRAAEKVFPPLDYSLTPPAQELIARDLHDNEWKFRHIFRGQPKRHLLTTGWSVFVSAKRLVAGDSVLFIWNDKNQLLLGIRRANRPQTVMPSSVLSSDSMHIGLLAAAAHAAATNSRFTIFYNPRASPSEFVIPLAKYVKAVYHTRVSVGMRFRMLFETEESSVRRYMGTITGISDLDPVRWPNSHWRSVKVGWDESTAGERQPRVSLWEIEPLTTFPMYPSPFPLRLKRPWPSGLPSFPGLKDGDMSINPSLMWLQGGVGDQGIQSLNFQGFGVSPWMQPRLDTSMPGLQPDVYQAMAAAALQEMRTVDSSKLASQSLLQFQQSQSASNGPASLIPRQMLQQSHPQNAYVQSFQDNQASAQAQLLQQQLQRQHSYNDQRQQQQQVQQPQQMPQLSVQPQIPNIISALPHLASTSQSQPPTLQAIAPQCQQPSFSDSLGNPIATSDVSSVHSILGSLSQDGSSHLLSSNVSNPIITSSSIITKQVSVDPHLSSGVSHCVLPQVEQFGTQQSNVSELANLLPPFPGREYSSYQGTADPQNNLLFGVSIDSSSLMAQHGLQNLKNIGSENDSLSLPFAASNFTSAVGTDFPLNSEMTTSSCVDESGFLPSSENVEQVNTSNRTFVKVHKSGSFGRSLDISKFSSYDELRSELARLFGLEGQLEDPQRSGWQLVFVDRENDVLLLGDDPWQEFVNNVWYIKILSPLEVQQMGKEGLSSMSSVPGQRLSNNNCDDYLSRQELRSSSNGVASMGSLDY

>LcARF8A

MHADVETDEVYAQMTLQPLTPQEQKDTFVPMELGIPSKQPTNYFCKTLTASDTSTHGGFSVPRRAAEKVFPSLDFTQQPPAQELIARDLHDVEWKFRHIFRGQPKRHLLTTGWSVFVSAKRLVAGDAVLFIWNEKNQLLLGIRRAVRPPTVMPSSVLSSDSMHIGLLAAAAHAAATNSCFTVFYNPRASPSEFVIPLSKYVKSVFHTRISVGMRFRMLFETEESSVRRYMGTITGINDLDPVSWPNSHWRSVKVGWDESTAGDRQPRVSLWEIEPLTTFPMYPSLFPLRLKRPWHPGPSALHDNRDQAANLLMWLRGGTGEQALPSINFQSVGMHPWMQQRLDSSLLGNDANQQYQAMFAAGMQSGDPVRQQVMQLQQPFQHLQQTGSQNPLLQLKQEQHQDAIHQSISNNLLQPHSEILTQNMPRHLLQQLNNQPVEQAQQRIYNDALQSQSDQLLLRQQSNVPSPSFPKTDFMDSSTEIPASITPMQNMLGSLPEGNGSLLNFPRTGQSMHNDQLTQQQSWGQKYARSEVHAFLNSVSHPPSYNGKDAVVEPENCNSDAQTSTIFGLNMDSAGLLLPTTVSSFNTSVDPDMSSMPLGDSGFHNSLYGCMEDSSELLHDARNVDRPTPPETFVKVYKSGSVGRSLDISQL

>LcARF8B

MKLSTSGLGQQGHEGEKKCLNSELWHACAGPLVSLPTVGTRVVYFPQGHSEQVAASTNKEVDCQIPNYPSLPPQLICQLHNVTMHADVETDEVYAQMTLQPLTPQEQKDTFVPMELGIPSKQPTNYFCKTLTASDTSTHGGFSVPRRAAEKVFPSLDFTQQPPAQELIARDLHDVEWKFRHIFRGQPKRHLLTTGWSVFVSAKRLVAGDAVLFIWNEKNQLLLGIRRAVRPPTVMPSSVLSSDSMHIGLLAAAAHAAATNSCFTVFYNPRASPSEFVIPLSKYVKSVFHTRISVGMRFRMLFETEESSVRRYMGTITGINDLDPVSWPNSHWRSVKVGWDESTAGDRQPRVSLWEIEPLTTFPMYPSLFPLRLKRPWHPGPSALHDNRDQAANLLMWLRGGTGEQGLPSINFQSVGMHPWMQQRLDSSLLGNDANQQYQAMFAAGMQSGDPVRQQVMQLQQPFQHLQQTGSQNPLLQLKQEQHQDAIHQSISNNLLQPHSEILTQNMPRHLLQQLNNQPVEQAQQQRIYNDALQSQSDQLLLRQQSNVPSPSFPKADFMDSSTEIPASITPMQNMLGSLPEGNGSLLNFPRTGQSMHNDQLTQQQSWGQKYARSEVHAFLNSVSHPPSYNGKDAVVEPENCNSDAQTSTIFGLNMDSAGLLLPTTVSSFNTSVDPDMSSMPLGDSGFHNSLYGCMQDSSELLHDARNVDRPTPPETFVKVYKSGSVGRSLDISRYNELREELAQMFGIEGKLEDPLRSGWQLVFVDRENDVLLLGDDPWDAFVNNVWYIKILSPEDVQKMGEQGVESFNPSSGHSSRNGARDPVSSLEY

>LcARF5A

MKTGDLVGVGRAQTTLLEEMKLLKEMQDQSGTRKTINSELWHACAGPLVSLPQVGSLVYYFPQGHSEQVAVSTKRTATTQIPNYPNLPSQLLCQVHYVTLHADKETDEIYAQMSLQPVNSEKDVFPIPDFGLKPSKHPNEFFCKTLTASDTSTHGGFSVPRRAAEKLFPPLDYTMQPPSQELVVRDLHDNTWTFRHIYRGQPKRHLLTTGWSLFVGSKRLRAGDAVLFIRDEKSQLLVGVRRANRQQTALPSSVLSADSMHIGVLAAAAHAASNRTSFTIFYNPRACPSEFVIPLAKYRKSVYGTQISVGMRFGMMFETEESGKRRYMGTIVGISDLDPLRWPGSKWRNLQVEWDEPGCSDKQKRVSSWEIETPESLFIFPSLTSGLKRPFHPGLLGAEVEWGNLMKRPLPRPPEIGNGAIPYSSISNLCSEQLLRMMLRPQLVNHSGTFTSSLSQTSAVKGTLPIVFSFASQVEWDEPGCSDKQKRVSSWEIETPESLFIFPSLTSGLKRPFHPGLLGAEVEWGNLLKRPLPRPPEIGNGVIPYSSISNLCSEQLLRMMLRPPLVNHSGTFASSLPQTSAVKGTPLEEVKTLQATVNQKPQLIQSENTIIESQNCFQSGLDQADTINSSSSKINLPERPNPSSKCDKQTPAGTNIDSLKSEPEQSTHQLSHLTSTAECSEEKLVSSPLNPQTILNQLMLQNQNQGLMQLQSSMWPIQSPLESSVFQAQQVNLPQSDSANLNGLLPFSDAEEWIYNKVSGPLSMYGLQDPSTAFPEVINPPLPSTGQDMWDHQLNNLKFLSQVDQFTPFAQQDMSNLNSNGLRDLSDESNNQSGIYSCLNVDVSNGGGTVIDHSVSSAILDDFCTLKDANFQNPTDCLMNNFSSSQDVQSQITSVSLADSQAFSRQDFPDNSGGTSSSNVDFDENSLLQKTSWQQVAPPVRTYTKVQKAGSVGRSIDVTTFKNYEELRSAIERMFGLEGLLTDPRGSEWKLVYVDYENDVLLVGDDPWEEFVGCVRCIRILSPQEVQQMSEEGMKLLNSAAMQGIDCSMAGGDRA

>LcARF5B

MKTGDLVGVGRAQTTLLEEMKLLKEMQDQSGTRKTINSELWHACAGPLVSLPQVGSLVYYFPQGHSEQVAVSTKRTATTQIPNYPNLPSQLLCQVHYVTLHADKDTDEIYAQMSLQPVNSEKDVFPIPDFGLKPSKHPNEFFCKTLTASDTSTHGGFSVPRRAAEKLFPPLDYTMQPPTQELVVRDLHDNTWTFRHIYRGQPKRHLLTTGWSLFVGSKRLRAGDAVLFIRDEKSQLLVGVRRANRQQTALPSSVLSADSMHIGVLAAAAHAASNRTSFTIFYNPRACPSEFVIPLAKYRKSVYGTQISVGMRFGMMFETEESGKRRYMGTIVGISDLDPLRWPGSKWRNLQVEWDEPGCSDKQKRVSSWEIETPESLFIFPSLTSGLKRPFHPGLLGAEVEWGNLLKRPLPRPPEIGNGVIPYSSISNLCSEQLLRMMLRPPLVNHSGTFASSLPQTSAVKGTPLEEVKTLQATVNQKPQLIQSENTIIESQNCFQSGLDQADTINSSSSKINLPERPNPSSKCDKQTPAGTNIDSLKSEPEQSTHQLSHLTSTAECSEEKLVSSPLNPQTILNQLMLQNQNQGLMQLQSSMWPIQSPLESTVFQAQQVNLPQSDSANLNGLLPFSDAEEWIYNKVSGPLSMYGLQDPSTAFPEVINPPLPSTGQDMWDHQLNNLKFLSQVDQFTPFAQQDTSNLNSNGLRDLSDESNNQSGIYSCLNVDVSNGGGAVIDHSVSSAILDDFCTLKDANFQNPTDCLMNNFSSSQDVQSQITSVSLADSQAFSRQDFPDNSGGTSSSNVDFDENSLLQKTSWQQVAPPVRTYTKVQKAGSVGRSIDVTTFKNYEELRSAIERMFGLEGLLTDPRGSEWKLVYVDYENDVLLVGDDPWEEFVGCVRCIRILSPQEVQQMSEEGMKLLNSAAMQGIDCSMAGGDRA

>LcARF7A

MNQWMTRVAGDKKIINSELWHSCAGPLVNLPAAGTHVVYFPQGHSEQVAASMRKDVDGQIPNYPNLPSKLLCLLHNVTLHADPETDEVYAQMTLQPVPSFDKDALLRSDLALKSNKPLTEFFCKTLTASDTSTHGGFSVPRRAAEKIFPQLDFSMQPPAQELVARDLHDNVWTFRHIYRGQPKRHLLTTGWSLFVSGKRLFAGDSVLFIRDEKQQLLLGIRRANRQPANLSSSVLSSDSMHIGILAAAAHAAANNSPFTVFYNPRASPSEFVVPLAKYYKAVYSSQISLGMRFRMMFETEESGTRRYMGTITGISDLDPVRWKNSQWRNLQVGWDESNAGERRNRVSIWEIEPVTAPFFICPPPFFRSKHSRSDDESDIDNLFKRTMPWLGDEFSMKDSQALPALSLVQWMNMQQNPSLANTVQSNYLHSLSGSVLQNLAGADLSRQLCLQSQIPQPNNIQFNAQRPPQQAQQVEQLQKLPSMVNQLGSIMPPQQQMGDIAQQSRQNQITKGNNLTNGRFSHPPQQPKLQQQQPGMLPEMPGHVGLPPTQIINQPSTAGSNVLTGAAGAGQSVVTDDLPSCSTSPSTNNCQNVVSPMMHNRPNRSTTMGEDMAQSTTMALCSSGLETMSYNGNLVKDFQHKSDVKPSLNISRNQNQGFLAPQTYLNGATTQTDYLDTSSSTTSVCLSQNDVHFQHNNNSLSYHPPSMLRDVSQDGEGRADPRSSLPYGASIDDCEMFFKSCILL

>LcARF7B

MKTPANGAAGASSVAGXXXXXTPEGDKKMINSELWHSCAGPMVNLPAAGTHVVYFPQGHSEQVAASMGKDVDGQIPNYPNLPSKLLCLLHNVTLHADPETDEVYAQMTLQPVPSFDKDALLRSDLALKSNKPQTEFFCKTLTASDTSTHGGFSVPRRAAEKIFPQLDFSMQPPAQELEARDLHDNVWIFRHIYRGQPKRHLLTTGWSLFVSGKRLFAGDSVLFIRDEKQQLLLGIRRANRQPANLSSSVLSSDSMHIGILAAAAHAAANNSPFTIFYNPRASPSEFVVPLAKYYKAVYSSQISLGMRFRMMFETEESGTRRYMGTITGISDLDPVRWKNSQWRNLQVGWDESNAGERRNRVSIWEIEPVTAPFFICPPPFFRSKHSRSDDESDIDNLFKRTMPWLGDEFSMKDSQALPALSLVQWMNMQQNPSLANTVQSNYLHSLSGSVLQNLAGADLSRQLGLQSQIPQPNNIQFNAQRLPQQAQQIEQLQKLPSTVNQLGSIMPPQQQMGDITQQSRQNMITQTLPSGQIQAQILQPQGLVQNNNIVHQQPSFQNPQVPVNLPQNLQQQQQQQQQHIMGQNQQQNLMQSQLPDQVNQHLQMTDKQIQLQLLQKLQQQQQSFLAQQSALQQPAQLPQIQDQHRQLLDVSQSFSRSVTSTQMLDLPQATSTSLPQSNLISQQITKGNNLTNGRFSHPPQQPKLQQQQPGMLPEMPGHVGLPPTQITNQPSTAGSNVLTGAAGAGQSVVTDDLPSCSTSPSTNNCQNVIPPMMHNRPNRSTTMGEDMAQSTTIALCSSGLETMSYNGNLVKDFQHKSDVKPSLNISRNQNQGFLAPQTYLNGATTQTDYLDTSSSTTSVCLSQNDVHFQHNNNSLSYHPPSMLRDVSQDGEVRADPRSSLPYGANIDNIVYKRGAVGRSIDITRYSGYDELKQDLARRFGIEGQLEDHQRIGWKLVYVDHENDVLLVGDDPWEEFVSCVRCIKILSPQEVQQMSLDGDFGNSVLPNQACSSSDNGNT

>LcARF19A

MKAPANGFLANSAEGDRKSINSELWHACAGPLVSLPPVGSLVVYFPQGHSEQVAASMQKENDFVPNYPNLPSKLICMLHNVTLHADPETDEVYAQMTLQPVNKYEKEALLASDMGLKQNRQPTEFFCKTLTASDTSTHGGFSVPRRAAEKIFPPLDYSMQPPAQELLAKDLHDTTWTFRHIYRGQPKRHLLTTGWSVFVSTKRLFAGDSVLFIRDEKSQLLLGIRRANRQQPALSSSVISSDSMHIGILAAAAHAAANNSPFTIFYNPRASPSEFVIPMAKYNKAMYTQVSLGMRFRMMFETEESGVRRYMGTITGISDLDAVRWKGSQWRSLQVGWDESTAGERPSRVSIWEIEPVVTPFYICPPPFFRPKFPKQPGMPDDESDIENAFKRAMPWLGDDFGMKDAPNSIFPALSLVQWMSMQQNNQFPAAQSGLFPSMVSSTAMHNNLSTDDPSKLLNFQASALSTPTIQFNKANPQNQANQLPQPPIAWPQQQQLQQLLQTPLSQQQQQHFLRDSSRNNKHNHCQLHHSHNLNHHYHDSSSRSSSNNHSHIRKYSFQLL

>LcARF19B

MKAPANGFLANSAEGDRKSINSELWHACAGPLVSLPPVGSLVVYFPQGHSEQVAASMQKENDFIPSYPNLPSKLICMLHNVTLHADPETDEVYAQMTLQPVNKYEKEALLASDMGLKQNRQPTEFFCKTLTASDTSTHGGFSVPRRAAEKIFPPLDFSMQPPAQEIVAKDLHDTTWTFRHIYRGQPKRHLLTTGWSVFVSTKRLFAGDSVLFIRDEKSQLLLGIRRANRQQPALSSSVISSDSMHIGILAAAAHAAANNSPFTIFYNPRASPSEFVIPMAKYNKAMYTQVSLGMRFRMMFETEESGVRRYMGTITGISDLDALRWKGSQWRSLQVGWDESTAGERPSRVSIWEIEPVVTPFYICPPPFFRPKFPKQPGMPDDESDIENAFKRAMPWLGDDFGMKDAPNSIFPALSLVQWMSMQQNNQFPAAQSGLFPSMVSPTNFLTRNQQGPAILAGDSVVEPASNLVQELHNKSDIRMKHELLSSKGPDHLKYKGGVTDQLEASSSGTSYCLDPSNIQQNFSLPTYCLDGDVQSNSRNNVPFAANMDGMAPDTLLSRGYDSQKDLQNLLSNYGGAPRDIETELSTAGISSQSFGVPNISFKPSCSNDVAINEAGVLSGGLWANQTQRMRTYTKVQKRGSVGRSIDVTRYKGYDELRHDLARMFGIEGQLEDPQSSDWKLVYVDHENDILLVGDDPWEEFVSCVQSIKILSSAEVQQMSLDGDLGNNANLPVPNQACSGTDSGNAWRAQYDDNSATSFR

>LcARF17A

MPQQRRPSPSQPSRVDPAVWRACAGSLVQIPTVHSRVYYFPQGHLERSSPSPTVLSSLNISSAVIQCVVSQLHFLADPVTDQVFAKVLLTPVNTLEPSREQQQPQHQMEDRNVENYVHENVVAPFAKILTPSDANNGGGFSVPRFCADSIFPPLNYQADPPVQTLSITDIHGHVWDFRHIYRGTPRRHLLTTGWSKFVNRKKLIAGDSVVFMRDSMGMLFVGIRRAERYGSSGGDGARWGEHSGVKVEAFTEALERASLGLPFEVVYYPRTGWPDFIVRADVVEAAMKIFWTAGVRVKMVTETEDSSRVTWFQGTVSDASMPTTGPWCGSPWRMLEVTWDEPEVLLNVKRVSPWQLEFVSSSLPLHTPFPSAKRLKFPQNSGLVTDGEEDIFSPLRGLTNSTMGQMNPSSLNCNTFPVGMQGARQNTNSIFSLSNFISENTHQTCTENTTGINIVPKLKNVCTELNMGSSQSENLSPDSHSSAYSFGTELGNQGCNSSKIGASSFQLFGKIIHMNQPVESGFDDVACVEDDGGKEFNETEGVNKPTDLSLTYTALLNRFGVQCQGESADEETCSL

>LcARF17B

MPQQRRPSPSQPSRVDPAVWRACAGSLVQIPTVHSRVYYFPQGHLERSSPSPTVLSSLNISSAVIQCVVSQLHFLADPVTDQVFAKVLLTPVNTLEHSREQQQPRHQMEDRNVENYVHENVVAPFAKVLTPSDANNGGGFSVPRFCADQPPLSLDPPVQTLSISDIHGHVWDFRHIYRGTPRRHLLTTGWSKFVNRKKLVAGDSVVFMRDSMGMLFVGIRRAERYGSSGGDGSRWGEHSGVKVEAVTEALERASLGLPFEVVYYPRTGLPDFIVRADVVEAAMKIFWTAGVRVKMVTETEDSSRVTWFQGTVSDASMPTTGPWCGSPWRMLEVTWDEPEILLNVKRVSPWQLEFVSSSLPLHTPFPSAKRLKFPQNSGLVTDGEEDIFSPLRGLTNSTMGQMNPSSLNCNTFPVGMQGARQNTNSIFSLSNFISENTHQTCTENTTGINIVPKLKNVCTELNMGSSQSENLSPDSHSSAYSFGTELGNQGCNSSKIGASSFQLFGKIIHMNQPVESGFDDVACVEDDGGKEFNETEGANKPTDLSLTYTALLNRFGVQCQGESADEETCSL

>LcARF16A

MNRVMDSDKNSSIKKNSTESCLDAQLWHACAGGMVQIPPINSKVVYFPQGHLEHAKGNNTELSNLHIPCMIPCRVSGIKYMADTETDEVYAKIRLISLKNNGLDDFEDVGKGECGEKPPSFAKTLTQSDANNGGGFSVPRYCAETIFPRLDYSAEPPVQTILAKDVHGEVWKFRHIYRGTPRRHLLTTGWSNFVNQKKLVAGDSIVFLRADNGDLCVGIRRAKKGGIADGSDYSSVGSWNSSGFPFASGYSVYVRGDDSKLMRRNSNTDFRANVSADSVVRAATLAANAQPFEVVYYPRASTPEFVVKASAVRAAMQIQWCSGMRFKMAFETEDSSRISWFMGTISSVQVADPIRWPNSPWRILQVAWDEPDLLQNVKRVNPWLVELVSSIPAIHLSTFSPPRKRLRLPEHPEFPLINQVPMPSFSRNPVMFSSPLGCVRDSTPAGIQGARHAHYGPSLSDLQFNKLQLGQFPVGFQHHHHHPTMAPRIPSCNFISDTENNDNISSWLTMGNPTQSFKENNETKTPHILLFGQLILPQQNSSQSCSGDTLRNSSSDGNLEKTVISSDGSGSAVHQNGPLENSSDEGSPWCKDHQKSYISLETGHCKVFMESEDVGRTLNLSVLGSYEELYGKLANMFGIESSEMFSNVSYRDAAGSTKHTGDEPFSEFLKTARRLTILTDSSSDNVGS

>LcARF16B

MNRVMDSGKNSSIKKNSTENCLDAQLWHACAGGMVQIPPVNSKVVYFPQGHLEHAKGNNTELSNLHIPCMIPCRVSGIKYMADTETDEVYAKIRLISLKNNGLDDFEDVGKGECGEKPPSFAKTLTQSDANNGGGFSVPRYCAETIFPRLDYSAEPPVQTILAKDVHGEVWKFRHIYRGTPRRHLLTTGWSNFVNQKKLVAGDSVVFLRADNGDLCVGIRRAKKGGIGDGSDYSSVGSWNSSGFPFASGYSVYVRGDDSKLMRRNSNTDFRANVSADSVVQAATLAANAQPFEVVYYPRASTPEFVVKASAVRAAMQIQWCSGMRFKMAFETEDSSRISWFMGTISSVQVADPIRWPNSPWRILQVAWDEPDLLQNVKRVNPWLVELVSSIPAIHLSTFSPPRKRLRLPEHPEFPLINQVPMPSFSRNPVMFSSPLGCVRDSTPAGIQGARHAHYGPSLSDLHFNKLQSGQFPVGFQHHHHPTMPPRIPSCNFISDTENNDNISSLLTMGNTTQSFKENNETKTPHILLFGQLILPQQNSSQSCSGDTLRNSSSDGNLEKTVISSDGSGSAVHQNGPLENSSDEGSPWCKDHQKSYISLETGHCKVFMESEDVGRTLNLSVLGSYEELYRKLANMFGIERSEMFSNVSYRDAAGSTKHTGDEPFSEFLKTARRLTILTDSSSDNVGS

>LcARF16C

MDKCLDSQLWHACAGGMVQMPPVNSKVFYFPQGHAEHACGPVDFRNCPRIPPYILCRVSAIKFMADPETDEVFAKIKLVPISNNEPGFDDDVILNREQEKPPSFAKTLTQSDANNGGGFSVPRYCAETIFPRLDYSADPPVQTILAKDVHGETWKFRHIYRGTPRRHLLTTGWSTFVNHKKLVAGDSIVFLRAENGDLCVGIRRAKRGIGGGPEVSSGWNGNYVSPYGGFTAFLREDEGKLMRNGNGSSSHSGNGGLMGKGKVRPESVIEAATLAANRQPFEVVYYPRASTPEFCVKASLVKAALQIRWCSGMRFKMAFETEDSSRISWFMGTISSVLVSDALYWPDSPWRLLQVTWDEPDLLQNVKRVSPWLVELVSNMPAFHLSTFSPPRKKTRLPQHPDFPLDGQLPMPTFPGNLLGHNSPFGCLPDNTPAGMQGARHAHYGLSLSDHHLNKLQSGLFSAGFPPLDRAAPPTRASSSPVIQKPSMSENVSCLLTMAHSTQTSKKPDNAKTPQLVLFGQPILTEQQISLSCSGDTVSPVLTGNSSSEGNLDKMANFSDNSGSAVHQQGLSERSFCEGLQWYRDNRQETDNLETGHCKVFMESEDVGRTLNLSLLGSYDELYKKLADMFGIENSETLSHVLYRDVTGAVKHIGDEPFSDFMKTARRLTILMDSSSDNVGM

>LcARF16D

MDKCLDSQLWHACAGGMVQMPPVNSKVFYFPQGHAEHACGPVDFRNCPRIPPYILCRVSAIKFMADPETDEVFAKIKLVPISNNEPGFDDDVILNREQEKPPSFAKTLTQSDANNGGGFSVPRYCAETIFPRLDYSADPPVQTILAKDVHGETWKFRHIYRGTPRRHLLTTGWSTFVNHKKLVAGDSIVFLRAENGDLCVGIRRAKRGIGGGPEVSSGWNGNCVSPYGGFTAFLREDEGKLMRNGNGNVNGSSSHSGNGGLMGKGKVRPESVIEAATLAANRQPFEVVYYPRASTPEFCVKASLVKAALQIRWCSGMRFKMAFETEDSSRISWFMGTISSVLVSDALYWPDSPWRLLQVTWDEPDLLQNVKRVNPWLVELVSNMPAFHLSTFSPPRKKTRLPQHPDFPLDGQLPMPTFPGNLLGHNSPFGCLPDNTPAGMQGARHAHYGLSLSDHHLNKLQSGLFSAGFPPLDRAAPPMRASSSPVIQKPSMSENVSCLLTMAHSTQTSKKPDNAKTPQLVLFGQPILTEQQISLSCSGDTVSPVLTGNSSSEGNLDKMANFSDNSGSAVHQQGLSERSFCEGLQWYRDNRQETDNLETGHCKVFMESEDVGRTLNLSLLGSYDELYKKLADMFGIENSETLSHVLYRDVTGAVKHIGDEPFSDFMKTARRLTILMDSSSDNVGM

>LcARF10A

METEKSIDPQLWHACAGSMVQIPPMNSTVFYFPQGHAEHCFGSVNFPSSTTPIPPLLLCRVTCLKFLADSETDEVYAKIRLVPLPATSDVDFEDSTNSMSLNSDTDSEKPASFAKTLTQSDANNGGGFSVPRYCAETIFPRLDYTADPPVQTVVAKDVHGEIWKFRHIYRGTPRRHLLTTGWSTFVNQKKLVAGDSIVFLRAQNGDLCVGIRRAKKGIDSPSSGWNSNSGSGVGVSPYGGFSCFLREEENKMMRNGAMSLNSNGSCNSSGNLRGSGGGSKVKPDAVVEAVALAVGGQPFEVVYYPRASTPEFCVKASAVRAAMRVHWCSGLRFKMAFETEDSSRISWFMGTISSVQVADPIRWPNSPWRLLQVTWDEPDLLQNVKRVSPWLVELVSNMPVLHLSPFSPPRKKLRLPQQLDFPLDGQFSMPSFSGNPLGPSSPLCCLSDNTPAGIQGARHAQFGTSLSDFHVYNKLQSGLFMSSFQRFNPQSRISDSIMANHTNSNENLSCLLTVGNSSQNLEKCNNVKKHQFVLFGQPILTEQQISHSCSSEAVTQVLGKSLSDEISGKAKASSDGSGITRELQISPQKSSSPEYFWNRGLQTTDPGLDTGHCKVFMESEDVGRTLDLSVFGSYEELYRRLANMFGIERSEMLNHVLYQDAAGAVKRIGDQPFSDFIRKAKRLTILMGSGSDNVGRTWITGMRNADNGLDTPNKTGPLSIFA

>LcARF10B

METEKSIDPQLWHACAGSMVQIPPMNSTVFYFPQGHAEHCFGSVNFPSSTTPIPPLLLCRVTCLKFLADSETDEVYAKIRLVPLPATSDVDFEESTNSMSLNSDTDSEKPASFAKTLTQSDANNGGGFSVPRYCAETIFPRLDYTADPPVQTVVAKDVHGEIWKFRHIYRGTPRRHLLTTGWSTFVNQKKLVAGDSIVFLRAQNGDLCVGIRRAKKGIDSPSSGWNSNSGSGVGVSPYGGFSCFLREEENKMMRNGAMSLNSNGSCNSSGNLRGSGGGSKVKPEAVVEAVALAVGGQPFEVVYYPRASTPEFCVKESAVRAAMRVHWCSGLRFKMAFETEDSSRISWFMGTISSVQVADPIRWPNSPWRLLQVTWDEPDLLQNVKRVSPWLVELVSNMPVLHLSPFSPPRKKLRLPQQLDFPLDGQFSMPSFSGNPLGPSSPLCCLSDNTPAGIQGARHAQFGTSLSDFHVYNKLQSGLFMSSFQRFNPQSRISDSIMANHTNSNENLSCLLTVGNSSQNLEKCNNVKKHQFVLFGQPILTEQQISHSCSSEAVAQVLGKSLSDEISGKAKASSDGSGITRELQISPQKSSSPEYFWNRGLQTTDPGLDTGHCKVFMESEDVGRTLDLSVFGSYEELYRRLANMFGIERSEMLNHVLYQDAAGAVKRIGDQPFSDFIRKAKRLTILMGSGSDNVGRIWITGMRNADNGLDTPSKTGPLSIFA

>LcARF4A

MEINPELTTTQSKKKDAETEVENKDFTAVFMENKEDFTAVFDEYSGGVYAETEAKNKGFTAVSMENKEDLTTFFDEDSDYNLDLWRALVGNRPSLPKKDDLVVYFPQDHLEYAALITSSSFSSSEVPTFNLQPEIICSVQHVQLIANKMTDETCAHVTLLPEQKLEGTNPKGEEVMKSGVSKAKDRRRPTKAALHVFPVTLSKPEDLQTISIPVDAYEVFRLRNFRFSTQVVSAEDVQGTVWIFYIEYADSPRKYQITRGWTEFLRRKRLSLKDTVAFVSCGDRKLRLVIKRFGCECGVPMSILAKYYQCSSVLSPVADAILKGSPFNVFYCGRVGSADFIIPYQKYMESITRLGMIGAKFETMDALSQRCSGVVTGISELDPFNWPNSKWKCLMVSWDNAANKKPLKEAVSPWEIVLKVPSSSDSSIDISPKLEKPRTVRLLNISLPLHDPAEREKQASVMGLEGFQRKINHVIDIIEQAPAPQTAFPKALSSDKLQRNSSPKAWEKLNTSLYDPAEREKQASAMVQEEFQRKEFPLKRIVDIIKQGPAPQTAFLEALSSDKLQRNSRSRAWNKLNTSFYDPAGTSFVLFDDKLEERGKQASVMVQEEFQQKEFPLKRIVDIIKQGPAPQTAFSEALSSDKLQRNSRSRAWNKLNTSFYDPAGTSFVLFDDKLEERGKQASVMVQEEFQRKEFPLKRIVDIIKQGRAPQIAFPKALSSDELQRNSRPRARKKRSGFDTDRDSSLASSKKRKSATIVTPTHFKISKRPDQELMICFERGVSKATHDKVSVDRLKPLQMQFSHAQQICESTAPFPDKGDVSSSIMTQPSSRLKRQRKLDSSPVDIETEGFLDELKPLEMQSPESKQVLPSTTPFPGKGDVSSSIMAQPSSRLKKELENSPLDSFRFTPLTGNCCLASLSESKSTTDVKQLKISNCSGHDVWFHLKGDHCFEYLEFDSCSFPSSTSLHIPIILKELKITNCLVTECVFTSLMDPKVSVESLEIDSCSSLHSISIDNIPASLRQLKIVNCMNLKSLSESIFESSKHHSVSLGPLNYSDLSKIDSIGTLELRKLVIQNCLELEYLPEDLHKLCNLDVLSIKNCCSLEYFPEGGLPKTCLRSLKISECENLKRLPNQLEEVTSLQKLSICGCSSLVSFQEGGLPPNLVSLRIINCENFFLDLSQWDLDKLKHLKLCSFIGGCPDS

>LcARF4B

MEIDLNHAFCNGDCEKNSGCVYCLSSSSSSSCSSNSGSSPLSSSIYLELWHACAGPLTSLPKKGNVVVYFPQGHLEQLASSSPYPPLEGSNFDLQPQIFCKVVDVQLLANKENDEVYTQVTLLPQPELVGPNSEGKDLEELSVDEDGNGGSPTKSTPHMFCKTLTASDTSTHGGFSVPRRAAEDCFPPLDYKQQRPSQELVAKDLHGVEWRFRHIYRGQPRRHLLTTGWSIFVSQKNLVSGDAVLFLRGDNGELRLGIRRAVRPRNGLPESVLQNSYPNALSLVANAISTKSMFHVFYSPRASHAEFVVPYQKYVKSITNPVCVGTRFKMRFEVDDSPDRRCNGVVTGMSDLDPYRWPNSKWRCFMVRWDEDIGSDHQERVSPWEIDRSVSLPPLNIQSSPRLKKLRTSLQATPPDYPVAAGGGGFLDFEDSIRTSKVLQGQENVGFVSSLYGRDTANRPLDFEMRAPIHQNLALPRLEKGNVTKFVARPTTYTGFMESDRFPKVLQGQEICPLRSLAGKVDLNLGAWGKPNLGCNSFNMYPAAKPSFYPLPSENLRTMYFPYNDMYKNGQDPTMRAYATSFQRENAQFNSPSIQKRVVGDEVRKPILLNEHKPADSIPTPTFKTTLRNQKEDTFSGTVAGCKLFGFSLTGETPSPSSQNSGKRSCTKVHKQGSLVGRAIDLSRLNGYNDLLTELEQLFSMEGLLRDPDKGWRILYTDSENDVMVVGDDPWHEFCNVVSKIHIYTKEEVEKMTIGMITDDTQSCLDQAPVIMEASKSSSVGQPDSSPTVIRI

>LcARF4C

MEIDLNHAFCNGDCEKNSGCVYCLSSSSSSSCSSNSGSSPLSSSIYLELWHACAGPLTSLPKKGNVVVYFPQGHLEQLASSSPYPPLEGSNFDLQPQIFCKVVDVQLLANKENDEVYTQVTLLPQPELVGPNSEGKELEELSVDEDGNGGSPTKSTPHMFCKTLTASDTSTHGGFSVPRRAAEDCFPPLDYKQQRPSQELVAKDLHGVEWRFRHIYRGQPRRHLLTTGWSIFVSQKNLVSGDAVLFLRGDNGELRLGIRRAVRPRNGLPESVLQNSYPNALSLVANAISTKSMFHVFYSPRASHAEFVVPYQKYVKSITNPVCVGTRFKMRFEVDDSPDRRCNGVVTGMSDLDPYRWPNSKWRCFMVRWDEDIGSDHQERVSPWEIDRSVSLPPLNIQSSPRLKKLRTSLQATPPDYPVAAGGGGFLDFEDSIRTSKVLQGQENVGFVSPLYGRDTANRPLDFEMRAPIHQNLALPGLEKGNVTKFVAHPTTYTGFMESDRFPKVLQGQEICPLRSLAGKVDLNLGAWGKPNLGCNSFNMYPAAKPSFYPLPSENLRTMYFPYNDMYKNGQDPTMRAYATSFHRENAQFNSPSIQKRVVGDEVRKPILLNEHKPADSIPTPTFKTTMRNQKEDTFSGTVAGCKLFGFSLTGETPSPSSQNSGKRSCTKVHKQGSLVGRAIDLSRLNGYNDLLTELEQLFSMEGLLRDPDKGWRILYTDSENDVMVVGDDPWHEFCNVVSKIHIYTKEEVEKMTIGMITDDTQSCLDQAPVIMEASKSSSVGQPDSSPTVIRI

>LcARF3A

MVGLIDLNTTEDDVDETQTQAQTPCSGSLSPSSSTASGAPASASGVSLELWHACAGPLISLPKRSSLVVYFPQGHLEHVSDFSASSSAYDLPPHLFCRVLDVKLHAEAASDEVYAQVSLLPENEQTEQKLREGNIDVEGEEEDVEVTGKASTPHMFCKTLTASDTSTHGGFSVPRRAAEDCFPPLDYTQQRPSQELVAKDLHGLEWRFRHIYRGQPRRHLLTTGWSAFVNKKKLVSGDAVLFLRGEDGELRLGIRRAAQVKGAATFPPVSSQQLSHTTVSDVANAMSMRRAFSICYNPRASASEFIIPVHKFLKSLDHTFQVGMRFRMRFETEDAAERRYTGLITGIADMDPVRWPGSKWRCLLVRWDDVEANRHSRVSPWEIEASGSVASSNSLISPGLKRTRIGLPPGKPEFPVPDGIGVSDFGESLRFQKVLQGQEILGFNTLYDGVDSRNLHPSDMRRCLPGSNRSGIAAIGDGMRNPHVNSDVSYKSIGFGESIRFHKVLQGQEIYPKSPYGRAPTTDEARGNGGLGTSDGVQVPGSRNGWSAMMQGYNTHTSPPAPSVQVSSPSSVLMFQLASNPVSNYNPVYSLNDQEKGQQISNHSLFHTAGIYGGKHASSSLGGHSFMGEDQGCMNSLGHSNELIQLGMSRPLVVQPAFRGSQDLVSSCKSSCRLFGFSLTEERHVAANIDNSRAPVPSPLTPGAAFVPRVGEQFRPKPPAMSTPVGNNCSNVSHFYAVKDMLSDIAL

>LcARF3B

MVGLIDLNTTEDDVDETQTQTPSSGSLSPSSSTASGAPASASGVSLELWHACAGPLISLPKRSSLVVYFPQGHLEHVSDFSASSSAYDLPPHLFCRVLDVKLHAEAASDEVYAQVSLLPENEQTEQKLREGNIDVEGEEEDVEVTGKASTPHMFCKTLTASDTSTHGGFSVPRRAAEDCFPPLDYTQQRPSQELVAKDLHGLEWRFRHIYRGQPRRHLLTTGWSAFVNKKKLVSGDAVLFLRGEDGELRLGIRRAAQVKGAATFPPVSSQQLSNTTVSDVANAISMRRAFSICYNPRASASEFIIPVHKFLKSLDHTFQVGMRFRMRFETEDAAERRYTGLITGIADMDPVRWPGSKWRCLLVRWDDVEANRHSRVSPWEIEASGSVASSNSLISPGLKRTRIGLPPGKPEFPVPDGIGVSDFGESLRFQKVLQGQEILGFNTLYDGIDSRNLHPSDMRRCLPRSNSSGIAAIGDGMRNPHVNSDVSYKSIGFGESIRFHKVLQGQEIYPKSPYGRAPTTNEARGNGGLGISDGVQVPGSRNGWSAMMQGYNTHTSPPAPSVQVSSPSSVLMFQLASNPVSNYNPVYSLNDQEKGQQISKHSLFHTAGIYGGKHASSSLGGHSFMGEDQGCMNSLGHSNELIQLGMSRPLVVQPAFRGSQDLVSSCKSSCRLFGFSLTEERHVAANIDNSRAPVPSPLTPGAAFVPRVGEQFCPKPPAMSTPVGNNCSNVSHFYAVKDMLSDIAL

>LcARF2E

MAGAGGNGKGRAGGGDQKGKAPSTDPHVHDDQSRKRTNINDALPTNNTDQCRQDGLYKALWRACAGPRVYVPCVGDYVYYFLQGHLEQIEAYANHEIRAEMPVYNVPSKVLCEVVHVQLMAETGTDEVFAQITLQPKTKQDMQNLEDGSSAPLPPKTKVCSFSKKLTQSDTSTHGGFSVPKRKADECLPQLDMSKEPPAQELIATDLHGYEWRFRHIFRGTTFSVYYHPWTSPSEFIIPVDQCDKLTAIDYSVGTRIRMAFETEDFTEKRIAGTVVGIEDVDRIRWPGSEWRCLRVKWDALSDRIRCPERVSPWNVELIEGANRKPASFLHRQKRPRPDNLSFPGCSNGVLQGQKNNVTGVKESGPLEPLSFPYLVPQKSSDWGHVLMHDPFYPCHNGRVSIASGIIVRSSGLPNYWPATYATNSVRNNAVLSRNISAPSGNSLNSSSKERRSLESKNENESPLSQSHGGKKYMLFGVNLANTSPPELPLLQQATSNELESRCSGPPMSQSSISGPFQVSEPSNRVSDIPSPKQCKNCRLLRSCTKVIKYGTALGRSVDLSRFDGYEELVYELDQMFDFNGTLIDGSSGWEITFIDDEGDMLLTGDHPWREFLCAVRRMFICPKEEIDKLNPSSPNPTCSLFVTITTTLPSSSLKIRDPPPLDVPVSSLPMSLLKNRPLHLPTITRRQFSSNSTTNDKEPPPEDTVEDLSVPEHWLLPSTAFEESEWLRVTLHKWLDDEYCPEPTNVEISKVAARSYYESLLEKQRDLGEILLKMARALESISYQDSFHGAFSSANAAVNLIVQRMGQP

>LcARF2F

MAGVGGNGKGRVGGGDQKGKAPITDPHVHDDQSRQRTNINDALPTNNTDQCRQDGLYKALWRACAGPRVYVPCVGDYVDYFLQGHLEQIEAYANHEIRAEMPVYNVPSKVLCEVVHVQLMAETGTDEVFAQITLQPKTKQDMQNLKDGNSAPLPPKTKVCSFSKKLTQSDTSTHGGCSVPKRKADECLPQLDMSKEPPAQELIATDLHGYEWRFRHIFRGQPKRHLLTSGWSIFVASKMLNSGDLCIFLRGEHGELRIGIRRLMKKQNCASTTIISSHSMQHGILAGAFHAISTGTTFSVYYHPWTSPSEFIIPVDQCDKLTAIDYSVGTRIRMAFETEDFTEKRIAGTVVGIEDVDRIRWPGSEWRCLRVKWDTLSDRIRCPERVSPWNVELIEGANRKPASFLHRQKRPRPDNLSFPGCSNGVLQGQQNNVTGVKESGPLEPLSFPYLVPQKSSDWGHVLMHDPFYPCHNGRVSIPSGIIVRSSGLPTYWPATYATNSVRNNAVLSRNISAPSGKSLNSSSKERRSLESKNENESPLSQSHGGKKYMLFGVNLANTSPPELPLLQQATSNELESRCSVPPMSQSGISETFQVSEPSNSVSDIPSGKQCKNCRLLKSCTKVIKYGTALGRSVDLSRFDGYEELVYELDQMFDFNGTLIDGSSGWEITFIDDEGDMLLTGDHPWKEFLCAVRRMFICPKEDIDKLNPSSPNPTSV

>LcARF1A

MAFPAAAVAPLNHSSGGPLSGGSTDALYRELWHACAGPLVSLPREGERVYYFPQGQPRRHLLTTGWSVFVSSKKLLAGDAFIFLRGENGELRVGVRRHMRQQTNMPSSVISSHSMHLGVLATASHAIATGTLFSIFYKPRFSGTIVGVEDNKTSAWPNSEWRSLKVQWDEPSSIVRPERVSAWELEPLVANTTTPPPPNSQPAQRNKRARPPVLPTPAPDLSVLGMWKSPVESQAFSYIDSQHGRDLYLSPKFSSATKANPLGFGGNSSLATVTGNSMYWPNRGENVMESFAPVVSKESSEKRQGTGNTYKLFGIQLVDNSNIEESLAAVTMSATGGDDRPVPSLDADSEQHSEPSNIPSVSCDAEKSCLRSPQESQSRQIRSCTKVHMQGIAVGRAVDLTRFDCYDDLLKRLEEMFDIEGELSGATEKWQVVYTDHEDDMMMVGDDPWHEFCSIVGKIFIYTAEEVKRLSPKIKLPSNEEVKPAKPDVDTAVNTEDRLSVVGHGC

>LcARF1B

MHQGLEQPMHSLNLPSKILCRVINVQHRAEPETDEVYAQITLFAESDQSEVTRPDPPLPEPEEYTVQSFCRTLVASDVSIHGGLSVPKKHAIECLPPLDITQQPPSHKLVATDLHGNEWEQNGELRVGVRRHKREEVNRPSLVISIGNINLDELATASQAIASGMMFSIVYKPRISRSGFIVSVDKYLEAMRYNFSVGMRFKMRYEGGEVLEQSGTVVGIEDYDRSTWPNSEWRCLKVQWDAHLSIPCPTRVSQWELEPF

>LcARF1C

MNLYNVRLPAPVVEDAKLEKDSFASVVSSIKLAFRHDDPESFFSSLKWISVLDLVSSTPQLQDSAICSGFGLRDSASAGSWKKVPETAHRILLQEMNFILPIAFEKSLFSIHVSAIAAPPAFRSSVRSSLGQLTSYLSRIGGGLVVAFPAAAAVPLNHSSGGPLSGVLSNDALYRELWHACAGPLVTLPREGECVNYFPQGHTEQLEASMHQGLEQPMHSLNLPSKILCRVINVQHRAEPETDEVYAQITLFAESDQSEVTRPDPPLPEPEEYTVQSFCRTLVASDVSIHGGLSVPKKHAIECLPPLDMTQQPPSHKLVATDLHGNEWCFRHILRGYIIQVNLDVICLQLDGVHSSLPKNWEQNGELRVGVRRHKREEVNRPSLVISIGNINLDELATASQAIASGMMFSIVYKPRISRSGFIVSVNKYLEAMRYNFSVGMRFMMRFEDGEVVEQSGTVVGIEDYDHSTWPNSEWRCLKVQWVEHSSISCLTRVSPWELEPF

>LcARF1D

MAGDLDTDVALYRELWHACAGPLASVPRAGEHVYYFPQGHLEQLQALTHDWTEQRVHYFELSSKILCRVVSVQLRAEPETEDVYAQITLLPAPVPSEDARQDPPAPEPRCTVLSLCRTLAASDVGTNGVFSISGMDADVCLPPLDMSQQPPCQELVAMDLHGNEWHFQHIFEDEPRCHLLTAGWSEYVISKKLVAGDAFILQRQVQGENGKLHVGVRRHVEKSRHSSIISNQSMWLGLLATASDAILCGTMFTVFYKPRTIRSNFIVNVEKYHEAMRYDFSVGMRFKMRFEGEEVPVQSFIGKIVAFENYCSSMWSNSKWRSLKVVVTHILGGNKAAKILANMGGMSLLQLSGESSFSINVTESTTLTTCLTTVAIAESNSRGLPFQNENLSII

>LcARF2A

MARSLRDSVVAIDGDDDWSERNGGGAVGGKALSRELWRACAGPLVTVPSKGDVVYYFPQGHMEQVEALMNQAADQQMPAYDISEKIVCRVFNVVLMAEPDTDEVFSQVTLLPLTKFQEEEAVAAQRERGPPPPRPRVYSFCKTLTASDTSTHGGFSVLRRHAEECLPPLDMSMQPPAQDLAAKDLHGNEWQFRHIFRGQPRRHLLQSGWSNFVGSKKLVAGDAFIFLRGENGELRVGVRRGLMQPNTVSSSVISSHSMHIGVLATAWHAVATGTMFTVYYKPRTSPADFMVPYDKYNEAARNNYTVGMRFKMRFEGEEGPEQRHSGTVVGVEDVDPIKWPGSMWRCLKVRWDETSSRNHPERISPWEAEFSVTPDADPLPVYRLKRPRPNMVSLSNGSSVHSTDGPTGMAGSRHSQQSCKFSERFVKQNPEVADQMKRCDANQESIYKLLPESRMHANSQFKMAESNMELQANDKEMHSQNPANIHSTVVRGYDRPHGLEFRRQAENWLSPLSSPSHAKKSSHPVELDAQPPAEKQQEVAKSKAVASCKLFGISLVNNYEPTAPAMPNANSVFGPQQRINFASDPVQDLASGILPDQLKCTKSAETALGGDKLGEPFQASDQLSRDVTSKLLVGSTRTGVKVHKQGSAVGRSVDLTKLNGYDELVTELDRIFEFNGELTAPNKKWLIVFTDDEGDMMLVGDDPWLEFCSMVRRIYVYTREEVNQMIPRPLNPKIKKTLFMADKKTGSTEAKSLAHA

>LcARF2B

MARSLRDSVVAIDGDDDWSERNGGGAVGGKALSRELWRACAGPLVTVPSKGDVVYYFPQGHMEQVEALMNQAADQQMPAYDISEKIVCRVFNVVLMAEPDTDEVFSQVTLLPLTKFQEEEAVAAQRERGPPPPRPRVYSFCKTLTASDTSTHGGFSVLRRHAEECLPPLDMSMQPPAQDLVGKDLHGNEWQFRHIFRGQPRRHLLQSGWSNFVSSKKLVAGDAFIFLRGENGELRVGVRRGLMQPNTVSSSVISSHSMHIGVLATAWHAVATGTMFTVYYKPRTSPADFMVPYDKYNEAARNNYTVGMRFKMRFEGEEGPEQRHSGTVVGVEDVDPIKWPGSMWRCLKVRWDETSSRNHPERISPWEAEFSVTPDADPLPVYRLKRPRPNMVSLSNGSSVHSTDRPTGMAGSRHSQQSCKFSERFVKQNPEVADQMKRCDANQESIYKLLPESRMHANSQFKMAESNMELQANDKEMHSQNPANIHSTVVRGYDRPHGLEFRRQAENWLSPLSSPSHAKKSSHPVELDAQPPAEKQQEVAKSKAVASCKLFGISLVNNYEPTAPAMPNANSVFGPQQRINFASDPVQDLASGILPDQLKCTKSAETALGGDKLGEPFQASDQLSRDVTSKLLVGSTRTGVKVHKQGSAVGRSVDLTKLNGYDELVTELDRIFEFNGELTAPNKKWLIVFTDDEGDMMLVGDDPWLEFCSMVRRIYVYTREEVNQMIPRPLNPKIKKTLFMADKKTGSTEAKSLAHA

>LcARF2C

MAAATSSEVSIKSSNETSRSPLEGQKGNSTNSGARRVIDPEMALYTELWHACAGPLVTVPREGERVYYFPQGHIEQVEASTNQVADQQMPVYDLPSKILCRVINVQLKAEPDTDEVFAQVTLLPDSNDEKVVEKEPPPQPPPRFHVHSFCKTLTASDTSTHGGFSVLRRHADECLPPLDMSRQPPTQELVAKDLHGNEWRFRHIFRGQPRRHLLQSGWSVFVSSKRLVAGDAFIFLRGENGELRVGVRRAMRQQNNVPSSVISSHSMHLGVLATAWHAISTGTMFTVYYKPRTSPAEFIVPFDQYMESVKNNYSIGMRFKMRFEGEEAPEQRFTGTIVGIEEADPQRWRDSKWRCLKVRWDETSTIPRPERVSPWKIEPALAPPALNPLPMPRPKRPRTNMLPSSPDSSVLTREGSSKVNVDPSSASGFSRVLQGQETLRGNFAERESNESENAEKSVAWPPQLEDEKIDVVSGSRRYGPESWVPSGRHEPTYTDLLSGFGPNADSSHGYGASFVDQSLPSACSARKSLLDQDGKFSLPARPWSLMPTPLSLKLAESNAKIPGQGGDVTYQVRGNVRYGGFGDYPMLHPHRIEQSHGNWLMPPPPQSQFENPTHSRELMPKPVLVQDREAGKSKDGNCKLFGIPLFSNPVVPELTVSQRNTMNDPGGHAHHQYCAFESDQKSEQTKGSKLADDGPAVNEQEKPSQPCQSHTKDTRNKPHSVSSRSCTKVHKQGIALGRSVDLSKFNNYEELIAELDRLFEFAGELMASKNWLIVYTDDEDDMMLVGDDPWQEEVQKMNPGSLSSKCEENPLSAEGLDAKEVKHLPLPSALNAEHC

>LcARF2D

MAAATSSEVSIKSSNETSRSPLEGQKGNSTNSGARRVIDPEMALYTELWHACAGPLVTVPREGERVYYFPQGHIEQVEASTNQVADQQMPVYDLPSKILCRVINVQLKAEPDTDEVFAQVTLLPDSNDEKVVEKEPPPQPPPRFHVHSFCKTLTASDTSTHGGFSVLRRHADECLPPLDMSRQPPTQELVAKDLHGNEWRFRHIFRGQPRRHLLQSGWSVFVSSKRLVAGDAFIFLRGENGELRVGVRRAMRQQNNVPSSVISSHSMHLGVLATAWHAISTGTMFTVYYKPRTSPAEFIVPFDQYMESVKNNYSIGMRFKMRFEGEEAPEQRFTGTIVGIEEADPQRWRDSKWRCLKVRWDETSTIPRPERVSPWKIEPALAPPALNPLPMPRPKRPRTNMLPSSPDSSVLTREGSSKVNVDPSSASGFSRVLQGQETLRGNFAERESNESENAEKSVAWPPQLEDEKIDVVSGSRRYGPESWVPSGRHEPTYTDLLSGFGANADSSHGYGASFVDQSLPYASSARKSLLDQDGKFSLPARPWSLMPTPLSLKLAESNAKIPGQGGDVTYQVRGNVRYGGFGDYPMLHPHRIEQSHGNWLMPPPPQSQFENPTHSRELMPKPVLVQDREAGKSKDGNCKLFGIPLFSNPVVPELTVSQRNTMNDPGGHAHHQYCAFESDQKSEQTKGSKLADDGSAVNEQEKPSQPCQPQTKDTRNKTHSVSARSCTKVHKQGIALGRSVDLSKFNNYEELIAELDRLFEFGGELMASKNWLIVYTDDEDDMMLVGDDPWQYGFFSQALIIYYI

>LcARF18A

MAHVEGNPRSSSISHVGTGTSCDDLYRELWKACAGPLVEVPRFGERVFYFPQGHVEQLEASTNQELQHQKPLFSLASKILCRVVNIMLLAEPESDEVYAQITLHPEQEQSEPTNPDQSPPESPKQTLHSFCKILTASDTSTHGGFSVLRKHATECLPPLDMNQATPTQELIAKDLHGYEWRFKHIFRGQPRRHLLTTGWSTFVTSKRLVAGDAFVYLRGENGELRIGVRRLARQQSPMPSSVISSQSMHLGVLATASHAVMTNTLFVVYYKPRTSQFIIGVNKYLESVKYGFSVGMRFKMRFEGEDSPERRFTGTIVGGGDSSPQWPGSEWRSLKIQWDEPATISRPERVSPWEIEPFVTSAPPLNLAQPAVKNKRPRSVDIQASEITTNSATSAFWYQGSNHSHDLTQLGSAAEVQSGERQVWPMRQKETDCNLVSSTNGCNSRFPPEGMWPSSPHFNVSLNLFPDSSEDGKIAVPRSVLSSYASSVPSRLSNDLTHDQVEKRKQSETSTSCRLFGIVLKNNSNAAAPLGKEVISSTMDSSGTKGSVSAACTADKDPNFDSSKSFMELNQVTMEMPLKELQNKQASSTSMRTRTKVQMQGIAVGRAVDLTVLKGYNDLIDELEKMFEIQGQLRPHDKWAVVFTDDEGDMMLVGDDPWPEFCKMVKKIYIYSNEEVKKLTARSKNAASSMEGEGTVISLDSEHRSEA

>LcARF18B

MAHVEGNPRSSSISHVGTGTSCDDLYRELWKACAGPLVEVPRFGERVFYFPQGHVEQLEASTNQELQHQKPLFSLASKMLCRVVNIMLLQSEPTNPDQSPPESPKQTVHSFCKILTASDTSTHGGFSVLRKHATECLPPLDMNQATPTQELIAKDLHGGENGELRVGVRRLARQQSPMPSSVISSQSMHLGVLATASHAVMTNTLFVVYYKPRTSQFIIGVNKYLESVKYGFSVGMRFKMRFEGEDSPERRFTGTIVGVGDISPQWPGSEWRSLKIQWDEPATIPRPERVSPWEIEPFVTSAPPLNLAQPAVKNKRPRSVDIQASAFWYQGSNHSHDLTQLGSAAEVQSGERQVWPMMQKETDCNLVSSTNGCNSRFPPEGMWPSSPHFNVSLNLFPDSSEDGKIAVPRSVLSSYASSVPSRLSNDLTHDQVEKRKQSETSRSCRLFGIVLKNNSNAAAPLGKEVTSSTMDSSGTKGSVPAACTADKDPNFDSSKSFMELNQVTLEMPLKELQNKQASTTSMRTRTKVQMQGIAVGRAVDLTVLKGYNDLIDELEKMFEIQGQLRPHDKWAVVFTDDEGDMMLVGDDPWPEFCKMVKKIYIYSNEEVKKLTARSKNAASSMEGEGTVISLDSEHRSET

>LcARF9A

MTGRVGSFSQPSSNSSDDLYEELWKACAGPLVDVPKSGEKVFYFPQGHMEQLEASTNQELNQRIPLFQLRPKILCRVMNIQLMAEQETDEVYAQITLLPEANQDEPTLPDPCPPESARPTVHSFCKVLTASDTSTHGGFSVLRKHATECLPPLDMNQSTPTQELVAKDLHGYEWRFKHIFRGQPRRHLLTTGWSTFVTSKRLVAGDTFVFLRGENGDLRVGVRRLGHQQSSMPSSVISSQSMHLGVLATASHAVATRTRFVVYYKPRTSQFIVGVNKYLEAINNKFAVGMRFKMRFEGEESPERRFSGTIVGVEDFSPHWNDSKWRSLKVHWDEPASISRPDRVSSWEIEPFVASVPSNVAQPVSAKNKRPRPPIEIPALDMSPTASASWNSRFTQSQDLTQLSVTAEGKRSDNHIAWHHKQTDFNNNSNSMSRTDGDWLTSPHVSFSQHLFQDTMDDNKSVSAWPAVSGYLTPQSSKVNNESMLDGPETGRKTEMATSCRLFGFDLINHSMASNTTEKAPVSSITTEGHILGTPPAADSDQKSDLSKAFKEMKEQLQVSPKETQSRQSCSTSTRSRTKVQMQGVAVGRAVDLTMLEGYDQLIDELEEMFDIKGQLHSRNKWEIVYTDDEGDMMLVGDDPWVEFCNMVKRIFICSSQDVKKMTPGSKLPMSSMEGEGILLSSDSAEN

>LcARF9B

MMTGRVGSFSQPSSNSSDDLYEELWKACAGPLVDVPKSGEKVFYFPQGHMEQLEASTNQELNQRIPLFHLRPKILCRVMNIQLMAEQETDEVYAQITLLPEANQDEPTLPDPCPPESARPTVHSFCKVLTASDTSTHGGFSVLRKHATECLPPLDMNQSTPTQELVAKDLHGYEWRFKHIFRGQPRRHLLTTGWSTFVTSKRLVAGDTFVFLRGENGDLRVGVRRLGHQQSSMPSSVISSQSMHLGVLATASHAVATRTRFVVYYKPRTSQFIIGVNKYLEAINNKFAVGMRFKMRFEGEESPERRFSGTIVGVEDFSPHWNDSKWRSLKVHWDEPASIPRPDRVSSWEIEPFVASVPSNVAQPVSAKNKRPRPPIEIPALDMSPTASASWNSRFTQSQDLTQLSVTAEGKRSDNHIAWHHKQTDFNNNSNSMSRTDGDWLTSPHVSFSQHLFQDTMDDNKSVSAWPAVSGYSTPQSSKVNNESMLDGPETGRKTEMATSCRLFGFDLINHSMASTTTEKAPVSSITTEGHILGTPPAADSDQKSDLSKAFKEMKEQLQVSPKETQSRQSCSTSTRSRTKVQMQGVAVGRAVDLTMLEGYDQLIDELEEMFDIKGQLRSRNKWEIVYTDDEGDMMLVGDDPWVEFCNMVKRIFICSSQDVKKMTPGSKLPMSSMEGEGILLSSDSAEN
