# Supplementary material for: Genome-wide characterization of the auxin response factor (ARF) gene family of litchi (Litchi chinensis Sonn.): phylogenetic analysis, miRNA regulation and expression changes during fruit abscission
Source: PeerJ. 2019 Apr 4;7:e6677. doi: 10.7717/peerj.6677 (PMC6451834; doi:10.7717/peerj.6677)
Supplement: Supplemental Information 5 [file peerj-07-6677-s005.docx]

Supplemental Data S2_Coding sequences of 39 *LcARFs*

>LcARF6A

ATGAGGCTCTCTTCAGCTGGTTTTAGTCCCCAAACCCAAGAAGGGGAGAAGAGGGTTTTGAACTCCGAACTTTGGCATGCTTGCGCTGGACCTCTTGTTTCTCTACCTGCAGTTGGGAGCCGCGTCGTTTACTTCCCGCAAGGCCATAGTGAGCAGGTTGCTGCATCAACGAACAAGGAAGTGGATGCTCATATACCTAACTACCCGAGTTTACCCCCACAACTTATCTGTCAGCTGCACAATGTTACCATGCATGCAGACATTGAAACAGATGAGGTATACGCACAGATGACTTTGCAACCTCTGAATCCGCAAGAGCAAAAGGATGCATACCTTCCAGCAGAGTTGGGCAACCCCAGCAAACAGCCAACAAATTACTTTTGTAAAACATTGACAGCCAGTGATACTAGTACACATGGAGGATTCTCCGTTCCTCGCCGAGCAGCCGAGAAAGTGTTTCCTCCATTGGACTTCACCCAGCAGCCTCCAGCTCAAGAGTTGATTGCTAGGGATCTGCATGATAATGAATGGAAATTTAGACATATATTTCGGGGCCAGCCCAAAAGGCACCTCCTTACAACAGGATGGAGTGTGTTTGTAAGTGCTAAGAGACTTATTGCTGGTGACTCGGTGCTTTTTATCTGGAATGAGAAAAATCAATTACTTCTTGGTATCCGCCGTGCAAATCGACCACCAACAGTGATGCCTTCATCAGTTTTATCAAGTGACAGCATGCACTTGGGGCTTCTAGCTGCTGCTGCTCATGCAGCTGCAACGAATAGCCGTTTTACCATATTTTATAACCCAAGGGCTAGTCCTTCAGAGTTTGTCATACCGCTGGCCAAGTACGTCAAAGCAGTTTATCACACTCGTGTTTCGGTTGGAATGCGCTTTAGGATGCTGTTTGAAACTGAAGAATCAAGTGTGCGTCGCTACATGGGCACCATAACTGGCATAAGTGACTTAGATCCTGTTAGGTGGCCTAACTCGCATTGGCGCTCAGTCAAGGTTGGCTGGGATGAATCAACAGCAGGAGAAAGGCAACCAAGAGTTTCATTGTGGGAGATTGAACCTTTAACAACATTCCCAATGTATCCTTCTCCTTTTCCACTGAGGCTTAAGCGACCATGGCCTCCTGGACTACCTGCTTTTCATGGTATAAAAGAAGAAGATATGGGATTGAATTCTCAACTTATGTGGCTCCGAGGAGATGGTGATCGTGGAATCCAGTCTCTGAACTTTCAGGGACTTGGAGTTACACCTTGGATGCAGCCAAGATTAGATGCCTCCATGCTTGGTTTGCAAACTGACATGTATCAAGCTATGGCTGCCGCCGCACTTCGAGAGATGAGGGCTGTAGATACCACCAAACCTGCTCATACTTCCCTTTTGCAATTCCAGCAACCTCAAAATCTCCCTAGTAGGTCTGCTGCTTTAATGCAACAACAGATGTTACAGCAGTCTCATCCTCAGCAGACATTTCTTCAAGGTGTTCAAGAGAACCAGCATCAACCTCATTCTCAGGCACAAACTCAGTCTCACCTACTTCAGCCACAGTTGCAGCACCCTCAGTCATTCCCTAACCAGCAGCATCAGTTGCCGCAGCCGCAGCAGCAGCAAGTTGATCATCAGCAGGTTCCAACTGCTGTCTCTGCTATGCCACAGTTTACTTCAGTCTCCCAATCCCAGTCACCTCCAATGCAAGCTGTCTCTTCACTCTGCCACCAGCAGAGTTTCTCTGATTCAAATGGGAACACTGTCACGAGCCCTATTGTTTCGCCGCTCCACAGTCTCTTGGGTTCATTTTCCCAGGACGAATCTTCCCATCTGATTAATTTGCCTAGAAGCAATCCCTTGATACCTTCTCCTACATGGCCTTCAAAGCGAGTTGCAGTTGAATCTCTTTTTCCTTCTGGAGGTCCTCAATGTGTTCTGCCCCAGGTGGAACAATTGGGGCCACCTCAGACAAATGTTTCTCAGAATTCTATATCATTGCCACCTTTTCCTGGTAGGGAGTGTTCAATAGACCAGGAAGGGAATGCTGATCCACACAGCCATCTTTTGTTTGGTGTTAATATCGAACCCCCATCACTTCTAATGCAGAATGAGATGTCAAGCCTCGGGGGAGTTGGGAGCAACAGTGACTCCACAACCATACCCTTTGCTTCTAATTATATGAGCACTGCAGGGACTGATTTTTCAGTTAATCCAGCTATTACAGCTACCAGTTGCATTGATGAATCAGGTTTTCTGCAGTCTCCTGAAAATGTGGGCCAAGGAAACCCACCAACAAGAACCTTTGTTAAGGTTCACAAGTCAGGGTCCTTCGGTAGATCATTGGATATTACAAAATTCAGCAGCTACCATGAGTTGCGCGGTGAACTTGCTCGCATGTTTGGCCTCGAAGGCCAGTTAGAGGACCCTCTGAGATCAGGCTGGCAGCTTGTATTTGTTGACCGGGAGAATGATGTTCTTCTCCTCGGTGATGACCCCTGGCCGGAGTTCGTAAATAGTGTGTGGTGTATCAAAATACTCTCACCGCCAGAAGTGCAGCAAATGGGAAAAAGAGGCATAGAGCTTCTGAACAAAGTACCAATTCCGAGGCTCTCCAACAATGGCTGTGATGATTATGCAAGCCGGCAGGACTCAAGAAATTTGAGCTCGGGTATAACTTCAGTGGGGTCACTGGATTACTGA

>LcARF6B

ATGAGGCCCTTTTCAGCTGGTTTTAGTCCCCAAACCCAAGAAGGGGAGAAGAGGGTTTTGAACTCCGAACTTTGGCATGCTTGCGCTGGACCTCTTGTTTCTCTACCTGCAGTTGGGAGCCGTGTCGTTTACTTCCCGCAAGGCCATAGTGAGCAGGTTGCTGCATCAACGAACAAGGAAGTGGATGCTCATATACCTAACTACCCGAGTTTACCCCCACAACTTATCTGTCAGCTGCACAATGTTACCATGCATGCAGACATTGAAACAGATGAGGTATACGCACAGATGACTTTGCAACCTCTGAATCCGCAAGAGCAAAAGGATGCATACCTTCCAGCAGAGTTGGGCAACCCCAGCAAACAGCCAACAAATTACTTTTGTAAAACATTGACAGCCAGTGATACTAGTACACATGGAGGATTCTCCGTTCCTCGCCGAGCAGCCGAGAAAGTGTTTCCTCCATTGGACTTCACCCAGCAGCCTCCAGCTCAAGAGTTGATTGCTAGGGATCTGCATGATAATGAATGGAAATTTAGACATATATTTCGGGGTCAGCCCAAAAGGCACCTCCTTACAACAGGATGGAGTGTGTTTGTAAGTGCTAAGAGACTTATTGCTGGTGACTCAGTGCTTTTTATCTGGAATGAGAAAAATCAATTACTTCTTGGTATCCGCCGTGCAAATCGACCACCAACAGTGATGCCTTCATCAGTTTTATCAAGTGACAGCATGCACTTGGGGCTTCTAGCTGCTGCTGCTCATGCAGCTGCAACGAATAGCCGTTTTACCATATTTTATAACCCAAGGGCTAGTCCTTCAGAGTTTGTCATACCGCTGGCCAAGTACGTCAAAGCAGTTTATCACACTCGTGTTTCGGTTGGAATGCGCTTTAGGATGCTGTTTGAAACTGAAGAATCAAGTGTGCGTCGCTACATGGGCACAATAACTGGCATAAGTGACTTAGATCCTGTTAGGTGGCCTAACTCGCATTGGCGCTCAGTCAAGGTTGGCTGGGATGAATCAACAGCAGGAGAAAGGCAACCAAGAGTTTCATTGTGGGAGATTGAGCCTTTAACAACATTCCCAATGTATCCTTCTCCTTTTCCACTGAGGCTTAAGCGACCATGGCCTCCTGGACTACCTGCTTTTCATGGTATAAAAGAAGAAGATATGGGATTGAATTCTCAACTTATGTGGCTCCGAGGAGATGGTGATCGTGGAATCCAGTCTCTGAACTTTCAGGGACTTGGAGTTACACCTTGGATGCAGCCAAGATTAGATGCCTCCATGCTTGGTTTGCAAACTGACATGTATCAAGCTATGGCTGCCGCCGCACTTCGAGAGATGAGGGCTGTAGATACCACCAAACCTGCTCATACTTCCCTTTTGCAATTCCAGCAACCTCAAAATCTCCCTAGTAGGTCTGCTGCTTTAATGCAACAACAGATGTTACAGCAGTCTCATCCTCAGCAGACATTTCTTCAAGGTGTTCAAGAGAACCAGCATCAACCTCATTCTCAGGCACAAACTCAGTCTCACCTACTTCAGGCACAGTTGCAGCACCCTCAGTCATTCCCTAACCAGCAGCATCAGTTGCCGCAGCAGCAGCAGCAAGTTGATCATCAGCAGGTTCCAACTGCTGTCTCTGCTATGCCACAGTTTACTTCAGTCTCCCAATCCCAGTCACCTCCAATGCAAGCTGTCTCTTCACTCTGCCACCAGCAGAGTTTCTCTGATTCAAATGGGAACACTGTCACGAGCCCTATTGTTTCGCCGCTCCACAGTCTCTTGGGTTCATTTTCCCAGGACGAATCTTCCCATCTGATTAATTTGCCTAGAAGCAATCCCTTGATACCTTCTCCTACATGGCCTTCAAAGCGAGTTGCAGTTGAATCTCTTTTTCCTTCTGGAGGTCCTCAATGTGTTCTGCCCCAGGTGGAACAATTGGGGCCACCTCAGACAAATGTTTCTCAGAATTCTATATCATTGCCACCTTTTCCTGGTAGGGAGTGTTCAATAGACCAGGAGGGGAACGCTGATCCACACAGCCATCTTTTGTTTGGTGTTAATATCGACCCCCCATCAATTCTAATGCAGAATGAGATGTCAAGCCTCGGGGGAGTTGGGAACAACAGTGACTCCACAACCATGCCCTTTGCTTCTAATTATATGAGCACTGCAGGGACTGATTTTTCAGTTAATCCAGCTATTACACCTACCAGTTGCATTGACGAATCATGTTTCCTGCAGTCTCCTGAAAATGTGGGCCAAGGAAACCCACCAACAAGAACCTTTGTTAAGGTTCACAAGTCAGGGTCCTTTGGTAGATCATTGGATATTACAAAATTCAGCAGCTACCATGAGTTGCGCAGTGAACTTGCTCGCATGTTTGGCCTCGAAGGCCAGTTAGAGGACCCTCTGAGATCAGGCTGGCAGCTTGTATTTGTTGACCGGGAGAATGATGTTCTTCTCCTCGGTGATGACCCCTGGCCGGAGTTCGTAAATAGTGTGTGGTGTATCAAAATACTCTCACCGCCAGAAGTGCAGCAAATGGGAAAAAGAGGCATAGAGCTTCTGAACAAAGTACCAATTCCGAGGCTCTCCAACAACGGCTGTGATGATTATGCAAGCCGGCAGGACTCAAGAAATTTGAGCTCGGGTATAACTTCAGTGGGGTCACTGGATTACTGA

>LcARF6C

ATGCGTTTGAAAACATGTGTGCCCCCAGCCCCCAAACCCAAGGTAAAAACATATGATAAGTTGATGACTTGTGAATTGACTTCTTTTGAATTGGCACCTCCTCAATTAGTGAAAACGACGGTAGACAATTTGGGGGAGAAGAAGTGTTTGAATTCTGAGCTTTGGCATGCATGTGCGGGTCCACTTGTGTCTTTGCCTCCTGTTGGGAGCCGTGTTGTCTACTTTCCTCAGGGTCACAGTGAGCAGGTTGCTGCCTCAACCAACAAGGAAGTAGATGCTCATATACCTAATTACCCTAGCTTACCCCCACAACTCATCTGTCAGCTTCACAATGTCACCATGCACGCAGATGTGGAAACAGATGAAGTATATGCACAGATGACCTTGCAACCACTGAGTCCGCAAGAGCAAAAAGATGTATACCTGCTGCCTGCGGAATTGGGTACTGGCAACAAACAGCCAACCAACTATTTTTGTAAAACTTTAACTGCAAGTGATACCAGCACTCATGGTGGCTTCTCTGTTCCTCGCCGAGCAGCTGAAAAAGTTTTTCCTCCCCTTGATTACTCACTAACACCTCCTGCTCAAGAACTGATTGCAAGGGATCTTCACGATAATGAATGGAAGTTCAGACATATATTTAGAGGCCAGCCCAAGAGGCATCTTCTTACGACAGGATGGAGTGTTTTTGTTAGTGCAAAAAGACTTGTTGCTGGTGATTCTGTCCTGTTTATCTGGAATGATAAAAATCAATTGCTTTTGGGTATCCGACGAGCAAATCGTCCTCAGACAGTCATGCCTTCATCAGTTCTGTCAAGTGATAGTATGCACATTGGTCTTCTTGCTGCAGCTGCACATGCAGCAGCCACAAATAGCCGTTTTACTATTTTTTATAATCCAAGGGCAAGTCCTTCAGAATTTGTCATTCCCCTGGCTAAGTATGTTAAAGCAGTCTATCATACGCGGGTCTCTGTGGGCATGCGATTTAGGATGCTGTTTGAGACAGAAGAGTCAAGTGTCCGTCGATACATGGGTACAATTACTGGTATTAGCGATTTAGATCCTGTCCGCTGGCCAAACTCCCATTGGCGCTCTGTTAAGGTAGGCTGGGATGAATCAACTGCTGGGGAGAGACAGCCAAGAGTATCATTGTGGGAGATTGAACCATTAACAACATTTCCTATGTATCCATCCCCTTTCCCTTTGAGACTGAAGCGACCATGGCCATCAGGATTGCCCTCCTTCCCTGTTGTGAAAGATGGTGATATGAGCATCAATTCTTCATTAATGTGGCTTCAAGGAGGGGTTGGAGATCAGGGAATTCAATCTTTGAACTTTCAAGGATTTGGGGTTTCACCCTGGATGCAGCCAAGGCTTGATACTTCTATGCCAGGTTTACAACCAGATGTATACCAAGCAATGGCTGCTGCTGCACTTCAGGAAATGAGAACAGTGGATTCTTCCAAATTAGCTTCCCAGTCTCTTCTGCAGTTCCAACAGTCACAAAGTGCATCCAATGGGCCCGCTTCTTTGATTCCAAGGCAGATGTTACAGCAGTCCCATCCTCAAAATGCTTATGTGCAGAGTTTTCAAGACAATCAGGCCTCTGCTCAGGCTCAGCTTCTTCAACAGCAGTTGCAGCGTCAGCACTCTTATAATGACCAACGACAGCAGCAGCAGCAAGTACAGCAACCTCAGCAAATGCCCCAGTTATCAGTTCAGCCACAGATCCCAAATATCATATCCGCTTTACCTCATCTTGCATCAACCAGCCAATCCCAGCCGCCAACGCTTCAGGCCATTGCTCCACAATGTCAGCAGCCGAGCTTTTCTGATTCTCTTGGTAACCCAATAGCTACATCTGATGTTTCCAGTGTGCACAGTATCTTAGGTTCATTATCTCAGGATGGCTCATCTCACTTACTCAGCTCAAATGTGTCCAACCCTATCATTACTTCCTCTTCCATCATAACCAAGCAAGTCTCAGTGGATCCACATCTTTCTTCTGGAGTGTCTCACTGCGTACTGCCCCAGGTGGAGCAGTTTGGGACACAACAATCAAATGTTTCTGAACTTGCTAATTTGCTACCACCATTTCCTGGGAGAGAGTACTCTTCTTACCAAGGTTCTGCTGATCCACAAAACAACCTCTTATTTGGGGTTTCCATTGATTCCTCATCTCTCATGGCACAACATGGGCTGCAAAATCTGAAAAACATTGGCAGTGAAAATGATTCGTTGTCCCTTCCATTTGCTGCTTCAAATTTTACTAGTGCCGTGGGAACTGATTTTCCACTTAACTCAGAGATGACAACTTCAAGTTGTGTTGATGAATCAGGCTTCTTGCCGTCCTCTGAGAATGTGGAGCAAGTGAATACATCAAACAGAACCTTTGTGAAGATCCCAAATATCATATCCGCTTTACCTCATCTTGCATCAACCAGCCAATCCCAGCCGGCAACGCTTCAGGCCATTGCTCCACAATGTCAGCAGCCGAGCTTTTCTGATTCTCTTGGTAACCCAATGGTTACATCTGATGTTTCCAGTGTGCACAGTATCTTAGGTTCATTATCTCAGGATGGCTCATCTCACTTACTCAGCTCAAATGTGTCCAACCCTATCATTACTTCCTCTTCCATCATAACCAAGCAAGTCTCAGTGGATCCACATCTTTCTTCTGGAGTGTCTCACTGCGTACTGCCCCAGGTGGAGCAGTTTGGGACACAACAATCAAATGTTTCTGAACTTGCTAATTTGCTACCACCATTTCCTGGGAGAGAGTACTCTTCTTACCAAGGTACTGCTGATCCACAAAACAACCTCTTATTTGGGGTTTCCATTGATTCCTCATCTCTCATGGCACAACATGGGCTGCAAAATCTGAAAAACATTGGCAGTGAAAATGATTCGTTGTCCCTTCCATTTGCTGCTTCAAATTTTACTAGTGCCGTGGGAACTGATTTTCCACTTAACTCAGAGATGACAACTTCAAGTTGTGTTGATGAATCAGGCTTCTTGCCGTCCTCTGAGAATGTGGAGCAAGTGAATACATCAAACAGAACCTTTGTGAAGGTTCACAAGTCAGGGTCCTTTGGGCGGTCACTGGACATTTCCAAGTTCAGCAGCTACGATGAGCTGCGCAGTGAGCTCGCTCGCTTGTTTGGCCTGGAAGGCCAACTAGAGGACCCTCAGAGATCAGGCTGGCAGCTTGTATTTGTAGACCGGGAGAATGATGTTCTTCTCCTTGGTGATGACCCTTGGCAGGAGTTCGTAAACAATGTGTGGTACATTAAGATACTATCTCCACTGGAAGTGCAACAAATGGGCAAAGAAGGCCTGAGTTCCATGTCTTCCGTCCCAGGCCAAAGGCTCTCCAACAACAACTGTGATGACTACCTGAGCAGACAGGAATTAAGAAGTTCCAGCAATGGGGTTGCATCCATGGGGTCTCTTGATTACTGA

>LcARF6D

ATGCGTATTAAATGGAGAGACGGCGCACACCACAAGGAGAAGGAAGGCGCGAGTTGCGACTTGCGAGGAAGGGAGAAGAAGTGTTTGAATTCTGAGCTTTGGCATGCATGTGCGGGTCCACTTGTGTCTTTGCCTCCTGTTGGGAGCCGTGTTGTCTACTTTCCTCAGGGTCACAGTGAGCAGGTTGCTGCCTCAACCAACAAGGAAGTAGATGCTCATATACCTAATTACCCTAGCTTACCCCCACAACTTATCTGTCAGCTTCACAATGTCACCATGCACGCAGATGTGGAAACAGATGAAGTATATGCACAGATGACCTTGCAACCACTGAGTCCGCAAGAGCAAAAAGATGTATACCTGCTGCCTGCGGAATTGGGTACTGGCAACAAACAGCCAACCAACTATTTTTGTAAAACGTTAACTGCAAGTGATACCAGCACTCATGGTGGCTTCTCTGTTCCTCGCCGAGCAGCTGAAAAAGTTTTTCCTCCCCTTGATTACTCACTAACACCTCCTGCTCAAGAACTGATTGCAAGGGATCTTCATGATAATGAATGGAAGTTCAGACATATATTTAGAGGCCAGCCCAAGAGGCATCTTCTTACGACAGGATGGAGTGTTTTTGTTAGTGCAAAAAGACTTGTTGCTGGTGATTCTGTCCTGTTTATCTGGAATGATAAAAATCAATTGCTTTTGGGTATCCGACGAGCAAATCGTCCTCAGACAGTCATGCCTTCATCAGTTCTGTCAAGTGATAGTATGCACATTGGTCTTCTTGCTGCAGCTGCACATGCAGCAGCCACAAATAGCCGTTTTACTATTTTTTATAATCCAAGGGCAAGTCCTTCAGAATTTGTCATTCCCCTGGCTAAGTATGTTAAAGCAGTCTATCATACGCGGGTCTCTGTGGGCATGCGATTTAGGATGCTGTTTGAGACAGAAGAGTCAAGTGTCCGTCGATACATGGGTACAATTACTGGTATTAGCGATTTAGATCCTGTCCGCTGGCCAAACTCACATTGGCGCTCTGTTAAGGTAGGCTGGGATGAATCTACTGCTGGGGAGAGGCAGCCAAGAGTATCATTGTGGGAGATTGAACCATTAACAACATTTCCTATGTATCCATCCCCTTTCCCTTTGAGACTGAAGCGACCATGGCCATCAGGATTGCCCTCCTTCCCTGGTCTGAAAGATGGTGATATGAGCATCAATCCTTCGTTAATGTGGCTTCAAGGAGGGGTTGGAGATCAGGGAATTCAATCTTTGAACTTTCAAGGATTTGGGGTTTCACCCTGGATGCAGCCAAGGCTTGATACTTCTATGCCAGGTTTACAACCAGATGTATACCAAGCAATGGCTGCTGCTGCACTTCAGGAAATGAGAACAGTGGATTCTTCCAAATTAGCTTCCCAGTCTCTTCTGCAGTTCCAACAGTCACAAAGTGCATCCAATGGGCCCGCTTCTTTGATTCCAAGGCAGATGTTACAGCAGTCCCATCCTCAAAATGCTTATGTGCAGAGTTTTCAAGACAATCAGGCCTCTGCTCAGGCTCAGCTTCTTCAACAGCAGTTGCAGCGTCAGCACTCTTATAATGACCAACGACAGCAGCAGCAGCAAGTACAGCAACCTCAGCAAATGCCCCAGTTATCAGTTCAACCACAGATCCCAAATATCATATCCGCTTTACCTCATCTTGCATCAACCAGCCAATCCCAGCCGCCAACGCTTCAGGCCATTGCTCCACAATGTCAGCAGCCGAGCTTTTCTGATTCTCTTGGTAACCCAATAGCTACATCTGATGTTTCCAGTGTGCACAGTATCTTAGGTTCATTATCTCAGGATGGCTCATCTCACTTACTCAGCTCAAATGTGTCCAACCCTATCATTACTTCCTCTTCCATCATAACCAAGCAAGTCTCAGTGGATCCACATCTTTCTTCTGGAGTGTCTCACTGCGTACTGCCCCAGGTGGAGCAGTTTGGGACACAACAATCAAATGTTTCTGAACTTGCTAATTTGCTACCACCATTTCCTGGGAGAGAGTACTCTTCTTACCAAGGTACTGCTGATCCACAAAACAACCTCTTATTTGGGGTTTCCATTGATTCCTCATCTCTCATGGCACAACATGGGCTGCAAAATCTGAAAAACATTGGCAGTGAAAATGATTCGTTGTCCCTTCCATTTGCTGCTTCAAATTTTACTAGTGCCGTGGGAACTGATTTTCCACTTAACTCAGAGATGACAACTTCAAGTTGTGTTGATGAATCAGGCTTCTTGCCGTCCTCTGAGAATGTGGAGCAAGTGAATACATCAAACAGAACCTTTGTGAAGGTTCACAAGTCAGGGTCCTTTGGGCGGTCACTGGACATTTCCAAGTTCAGCAGCTACGATGAGCTGCGCAGTGAGCTCGCTCGCTTGTTTGGCCTGGAAGGCCAACTAGAGGACCCTCAGAGATCAGGCTGGCAGCTTGTATTTGTAGACCGGGAGAATGATGTTCTTCTCCTTGGTGATGACCCTTGGCAGGAGTTCGTAAACAATGTGTGGTACATTAAGATACTATCTCCACTGGAAGTGCAACAAATGGGCAAAGAAGGCCTGAGTTCCATGTCTTCCGTCCCAGGCCAAAGGCTCTCCAACAACAACTGTGATGACTACCTGAGCAGACAGGAATTAAGAAGTTCCAGCAATGGGGTTGCATCCATGGGGTCTCTTGATTACTGA

>LcARF8A

ATGCATGCAGATGTTGAAACAGATGAAGTGTATGCTCAAATGACATTGCAGCCCTTAACACCGCAAGAACAGAAGGATACATTTGTTCCCATGGAATTGGGTATTCCCAGCAAGCAGCCTACCAATTATTTTTGCAAGACACTGACTGCAAGTGATACTAGTACACATGGAGGGTTTTCTGTTCCCCGTCGTGCTGCTGAGAAAGTCTTCCCTTCATTGGATTTTACCCAACAGCCGCCAGCTCAGGAACTCATTGCGAGGGATCTCCATGATGTCGAGTGGAAATTCAGGCACATCTTTCGTGGACAGCCCAAAAGGCATCTTCTTACAACAGGTTGGAGTGTGTTTGTCAGTGCCAAAAGATTAGTTGCTGGAGATGCTGTTCTTTTTATTTGGAATGAAAAGAATCAGCTTCTTTTGGGAATCCGCCGTGCTGTTCGACCACCAACTGTGATGCCATCTTCTGTTTTATCTAGTGATAGCATGCACATTGGACTTCTTGCTGCTGCGGCTCATGCTGCTGCAACTAATAGTTGTTTTACAGTTTTCTATAACCCAAGGGCTAGTCCATCTGAGTTTGTCATACCACTTTCAAAGTATGTGAAATCGGTATTTCACACCCGTATTTCAGTGGGGATGCGGTTTCGGATGCTTTTTGAGACTGAAGAATCAAGTGTTCGTAGGTACATGGGTACAATAACGGGCATAAATGATCTAGATCCTGTAAGCTGGCCGAATTCTCACTGGCGTTCTGTCAAGGTTGGTTGGGATGAGTCAACAGCAGGTGACAGGCAGCCAAGGGTATCTTTGTGGGAGATCGAGCCTCTAACAACTTTCCCAATGTATCCATCATTGTTTCCTCTTAGACTGAAGAGACCCTGGCATCCCGGGCCTTCAGCTTTGCATGATAACAGAGATCAGGCAGCTAATCTTTTAATGTGGTTAAGGGGTGGAACTGGAGAGCAGGCTCTGCCATCCATCAATTTCCAGTCTGTTGGTATGCATCCCTGGATGCAGCAGAGATTGGATTCGTCATTGCTAGGAAATGATGCCAATCAGCAGTACCAGGCAATGTTTGCGGCTGGCATGCAGAGTGGAGATCCTGTCAGACAGCAAGTTATGCAGTTGCAGCAGCCATTCCAACATCTGCAACAAACTGGCAGCCAGAATCCATTGTTGCAATTAAAGCAAGAACAGCATCAGGATGCAATTCATCAGTCTATCTCTAATAATCTACTGCAGCCACATTCTGAAATTTTAACACAGAACATGCCACGACATCTACTACAGCAACTTAACAATCAACCAGTGGAACAGGCACAACAACGCATTTATAATGATGCGCTTCAGAGTCAAAGTGATCAGCTCCTGCTGAGGCAGCAGTCTAATGTGCCTTCACCATCATTTCCTAAAACTGACTTCATGGACTCAAGCACGGAAATTCCAGCTTCTATCACCCCTATGCAGAACATGCTGGGTTCCTTGCCTGAAGGGAACGGCAGTCTTTTGAACTTTCCAAGAACTGGTCAGTCTATGCACAATGATCAGTTAACCCAACAACAGTCATGGGGACAGAAGTATGCACGTTCTGAGGTTCATGCTTTTCTAAACTCAGTGTCACATCCACCATCATATAATGGGAAAGATGCTGTGGTGGAACCTGAAAATTGTAACTCAGATGCCCAGACCTCCACTATATTTGGTCTTAATATGGATTCGGCTGGGCTCCTACTTCCAACCACAGTTTCCAGTTTTAATACTTCAGTTGATCCTGATATGTCCTCGATGCCATTAGGAGATTCTGGGTTTCACAATTCTCTGTATGGTTGCATGGAAGACTCCTCTGAGTTGCTGCACGATGCGAGGAATGTTGACCGACCAACTCCACCAGAGACATTTGTCAAGGTTTATAAATCGGGGTCGGTTGGACGTTCACTAGACATCTCCCAGCTATAA

>LcARF8B

ATGAAGCTTTCAACATCAGGGTTGGGTCAACAGGGTCATGAAGGGGAAAAGAAATGCTTGAATTCGGAGCTATGGCACGCCTGCGCTGGGCCATTGGTGTCCCTACCTACTGTGGGCACCCGTGTAGTCTACTTCCCTCAGGGTCATAGTGAGCAGGTAGCTGCCTCTACTAATAAAGAAGTTGATTGCCAGATTCCTAATTACCCAAGCTTGCCGCCCCAATTGATATGCCAACTCCACAATGTCACCATGCATGCAGATGTTGAAACAGATGAAGTGTATGCTCAAATGACATTGCAGCCCTTAACACCGCAAGAACAGAAGGATACATTTGTTCCCATGGAATTGGGTATTCCCAGCAAGCAGCCTACCAATTATTTTTGCAAGACACTGACTGCAAGTGATACTAGTACACATGGAGGGTTTTCTGTTCCCCGTCGTGCTGCTGAGAAAGTCTTCCCTTCATTGGATTTTACCCAACAGCCGCCAGCTCAGGAACTCATTGCGAGGGATCTCCATGATGTCGAGTGGAAATTCAGGCACATCTTTCGTGGACAGCCCAAAAGGCATCTTCTTACAACAGGTTGGAGTGTGTTTGTCAGTGCCAAAAGATTAGTTGCTGGAGATGCTGTTCTTTTTATTTGGAATGAAAAGAATCAGCTTCTTTTGGGAATCCGCCGTGCTGTTCGACCACCAACTGTGATGCCATCTTCTGTTTTATCTAGTGATAGCATGCACATTGGACTTCTTGCTGCTGCAGCTCATGCTGCTGCAACTAATAGTTGTTTTACAGTTTTCTATAACCCAAGGGCTAGTCCATCTGAGTTTGTCATACCACTTTCAAAGTATGTGAAATCAGTATTTCACACCCGTATTTCAGTGGGGATGCGGTTTCGGATGCTTTTTGAGACTGAAGAATCAAGTGTTCGTAGGTACATGGGTACAATAACGGGCATAAATGATCTAGATCCTGTAAGCTGGCCGAATTCTCACTGGCGTTCTGTCAAGGTTGGTTGGGATGAGTCAACAGCAGGTGACAGGCAGCCAAGGGTATCTTTGTGGGAGATCGAGCCTCTAACAACTTTCCCAATGTATCCATCATTGTTTCCTCTTAGACTGAAAAGACCCTGGCATCCCGGGCCCTCAGCTTTGCATGATAACAGAGATCAGGCAGCTAATCTTTTAATGTGGTTAAGGGGTGGAACTGGAGAGCAGGGTCTGCCATCCATCAATTTCCAGTCTGTTGGTATGCATCCCTGGATGCAGCAGAGATTGGATTCGTCATTGCTAGGAAATGATGCCAATCAGCAGTACCAGGCAATGTTTGCGGCTGGCATGCAGAGTGGAGATCCTGTCAGACAGCAAGTTATGCAGTTGCAGCAGCCATTCCAACATCTGCAACAAACTGGCAGCCAGAATCCATTGTTGCAATTAAAGCAAGAACAGCATCAGGATGCAATTCATCAGTCTATCTCTAATAATCTACTGCAGCCACATTCTGAAATTTTAACACAGAACATGCCACGACATCTACTACAGCAACTTAACAATCAACCAGTGGAACAGGCACAACAACAACGCATTTATAATGATGCGCTTCAGAGTCAAAGTGATCAGCTCCTGCTGAGGCAGCAGTCTAATGTGCCTTCACCGTCATTTCCTAAAGCTGACTTCATGGACTCAAGCACGGAAATTCCAGCTTCTATCACCCCTATGCAGAACATGCTGGGTTCCTTGCCTGAAGGGAACGGCAGTCTTTTGAACTTTCCAAGAACTGGTCAGTCTATGCACAATGATCAGTTAACCCAACAACAGTCATGGGGACAGAAGTATGCACGTTCTGAGGTTCATGCTTTTCTAAACTCAGTGTCACATCCACCATCATATAATGGGAAAGATGCTGTGGTGGAACCTGAAAATTGTAACTCAGATGCCCAGACCTCCACTATATTTGGTCTTAATATGGATTCGGCTGGGCTCCTACTTCCAACCACAGTTTCCAGTTTTAATACTTCAGTTGATCCTGATATGTCCTCGATGCCATTAGGAGATTCTGGGTTTCACAATTCTCTGTATGGTTGCATGCAAGACTCCTCTGAGTTGCTGCACGATGCGAGGAATGTTGACCGACCAACTCCACCCGAGACATTTGTCAAGGTTTATAAATCGGGGTCGGTTGGACGTTCACTAGACATCTCCCGCTATAATGAGCTGCGAGAAGAGCTGGCTCAGATGTTTGGAATTGAGGGGAAATTAGAAGACCCTCTTAGATCAGGCTGGCAGCTTGTATTTGTCGACAGGGAGAATGATGTGCTTCTCCTTGGAGACGACCCGTGGGATGCTTTTGTGAACAATGTTTGGTACATCAAAATTCTTTCACCAGAAGATGTGCAGAAAATGGGGGAGCAAGGGGTTGAATCCTTCAATCCAAGCTCCGGCCACAGCAGTCGAAATGGTGCTCGAGACCCTGTCAGCTCACTTGAATATTGA

>LcARF7A

ATGAATCAATGGATGACACGTGTTGCAGGAGACAAGAAGATAATAAACTCGGAGCTATGGCACTCCTGCGCTGGTCCTCTGGTCAATCTGCCTGCTGCTGGCACCCACGTCGTCTACTTCCCTCAAGGCCACAGCGAACAGGTTGCAGCATCTATGCGAAAGGATGTGGATGGTCAAATACCAAACTATCCAAACCTTCCATCAAAGCTACTATGTCTGCTTCACAATGTCACCTTGCATGCGGACCCGGAAACAGATGAAGTCTATGCTCAGATGACACTCCAACCTGTTCCTTCTTTTGACAAGGATGCATTGCTGAGATCGGATCTGGCTCTCAAGTCAAATAAACCACTAACGGAGTTCTTTTGTAAAACATTGACAGCAAGTGATACCAGCACTCATGGAGGTTTCTCTGTACCCCGCCGTGCAGCTGAGAAGATCTTCCCTCAACTTGATTTTTCTATGCAACCACCTGCTCAAGAACTTGTAGCCAGGGATTTGCATGATAATGTATGGACCTTCCGTCATATCTATCGTGGGCAACCTAAAAGACACTTGCTTACTACTGGTTGGAGTCTATTTGTTAGTGGAAAGAGGCTTTTTGCCGGTGACTCAGTCTTATTTATAAGAGATGAAAAGCAACAGCTTCTCTTGGGCATTAGACGAGCTAACAGGCAACCTGCAAATCTCTCATCATCAGTACTATCAAGTGATAGTATGCATATTGGAATTCTAGCAGCTGCTGCACATGCAGCTGCAAACAATAGTCCCTTCACTGTTTTTTATAATCCAAGGGCTAGTCCATCAGAATTTGTCGTCCCATTGGCCAAGTACTACAAGGCAGTGTATAGCAGCCAAATCTCACTAGGCATGCGGTTTAGGATGATGTTTGAAACTGAAGAGTCAGGAACAAGAAGGTACATGGGAACAATCACAGGTATCAGTGATCTGGATCCTGTTAGATGGAAGAACTCACAATGGCGCAACTTGCAGGTTGGCTGGGATGAGTCTAATGCTGGGGAAAGGCGCAATCGTGTCTCAATTTGGGAGATAGAACCAGTGACTGCTCCATTTTTCATCTGTCCCCCTCCATTCTTCAGATCCAAGCATTCGAGATCAGATGATGAGTCAGATATAGATAACCTATTCAAGAGGACCATGCCTTGGCTTGGTGATGAATTCTCTATGAAAGATTCCCAGGCTCTGCCTGCCCTGAGCTTGGTACAATGGATGAACATGCAGCAAAACCCATCCCTGGCAAACACAGTACAATCCAATTACCTCCATTCCTTGTCTGGGTCTGTTCTGCAAAACCTCGCTGGAGCTGATCTTTCTCGTCAATTATGTTTGCAATCACAAATACCTCAGCCAAATAACATTCAATTTAATGCCCAGAGGCCACCACAGCAAGCCCAACAAGTTGAACAGTTACAAAAGCTGCCGTCCATGGTGAATCAATTAGGCTCCATTATGCCACCTCAGCAACAGATGGGTGATATTGCTCAACAGTCAAGGCAAAATCAGATAACAAAGGGTAACAACCTGACAAATGGTCGATTCTCCCATCCTCCCCAGCAGCCAAAGCTTCAGCAGCAGCAACCTGGCATGCTGCCTGAAATGCCTGGACATGTTGGGCTTCCCCCCACCCAAATCATCAATCAACCTTCCACAGCCGGTAGTAATGTTTTGACAGGAGCTGCTGGAGCGGGGCAATCTGTAGTTACTGATGATCTTCCATCTTGTTCCACGTCACCTTCCACAAACAACTGTCAAAATGTTGTTTCACCAATGATGCACAATAGGCCCAACCGAAGCACAACAATGGGAGAGGACATGGCTCAGTCTACAACCATGGCCTTGTGCTCAAGTGGTTTAGAAACCATGTCATATAATGGTAACTTAGTTAAAGATTTTCAGCACAAATCTGATGTTAAGCCGTCATTGAACATATCCAGGAATCAAAACCAAGGTTTTTTGGCCCCTCAGACATACTTGAATGGTGCAACCACCCAGACAGATTATTTGGATACATCATCTTCAACAACGTCAGTTTGCCTTTCTCAGAATGATGTGCATTTCCAGCATAATAACAATTCACTGTCGTACCATCCACCGTCGATGTTGAGGGATGTGAGCCAAGATGGGGAAGGTCGGGCAGATCCCAGGAGCAGTCTTCCATATGGGGCTAGCATTGATGATTGTGAGATGTTCTTCAAATCCTGCATACTTTTGTAG

>LcARF7B

ATGAAGACACCGGCAAACGGTGCTGCCGGAGCTTCCTCTGTGGCAGGGCCNNNNNNNNNNNNNACCCCCGAAGGAGACAAGAAGATGATAAACTCGGAGCTATGGCACTCCTGCGCTGGTCCTATGGTCAATCTGCCTGCTGCTGGCACCCACGTCGTCTACTTCCCTCAAGGCCACAGCGAACAGGTTGCAGCATCTATGGGAAAGGATGTGGATGGTCAAATACCAAACTATCCAAACCTTCCATCAAAGCTACTATGTCTGCTTCACAATGTCACCTTGCATGCGGACCCGGAAACAGATGAAGTCTATGCTCAGATGACACTCCAACCTGTTCCTTCTTTTGACAAGGATGCATTGCTGAGATCGGATCTTGCTCTCAAATCAAATAAACCACAAACGGAGTTCTTTTGTAAAACATTGACAGCAAGTGATACCAGCACCCATGGAGGTTTCTCTGTGCCCCGCCGTGCAGCTGAGAAGATCTTCCCTCAACTTGATTTTTCTATGCAACCACCTGCTCAAGAACTTGAAGCCAGGGATTTGCATGATAATGTATGGATCTTCCGTCATATCTATCGTGGGCAACCTAAAAGACACTTGCTTACTACTGGTTGGAGTCTATTTGTTAGTGGAAAGAGGCTTTTTGCCGGTGACTCAGTCTTGTTTATAAGAGATGAAAAGCAACAGCTTCTCTTGGGCATTAGACGAGCTAACAGGCAACCTGCAAATCTCTCATCATCAGTGCTATCAAGTGATAGTATGCATATTGGAATTCTAGCAGCTGCTGCACATGCAGCTGCAAACAATAGTCCCTTCACTATTTTTTATAATCCAAGGGCTAGTCCATCAGAATTTGTCGTCCCATTGGCCAAGTACTACAAGGCAGTGTATAGCAGCCAAATCTCACTAGGCATGCGGTTTAGGATGATGTTTGAAACTGAAGAGTCAGGAACAAGAAGGTACATGGGAACAATTACAGGTATCAGTGATCTGGATCCTGTTAGATGGAAGAACTCACAATGGCGCAACTTGCAGGTTGGCTGGGATGAGTCTAATGCTGGGGAAAGGCGCAATCGTGTCTCAATTTGGGAGATAGAACCAGTGACTGCTCCATTTTTCATCTGTCCCCCTCCTTTCTTCAGATCCAAGCATTCGAGATCAGATGATGAGTCAGATATAGATAACCTATTCAAGAGGACCATGCCTTGGCTTGGTGATGAATTCTCCATGAAAGATTCCCAGGCTCTGCCTGCCCTGAGCTTGGTACAGTGGATGAACATGCAGCAAAACCCATCCCTGGCAAACACAGTACAATCCAATTACCTCCATTCCTTGTCTGGGTCTGTTCTGCAAAACCTCGCTGGAGCTGATCTTTCTCGTCAGTTAGGTTTGCAATCGCAAATACCTCAGCCAAATAACATTCAATTTAATGCCCAGAGGCTACCACAGCAAGCCCAACAAATTGAACAGTTACAAAAGCTGCCGTCCACGGTGAATCAATTAGGCTCCATTATGCCACCCCAGCAACAGATGGGCGATATTACTCAACAATCAAGGCAAAATATGATTACTCAAACTTTACCCTCTGGTCAAATTCAGGCCCAAATTCTTCAGCCACAAGGTCTTGTCCAAAATAATAATATTGTTCACCAACAGCCATCTTTTCAAAACCCTCAGGTTCCAGTTAACCTTCCTCAGAACCTGCAGCAGCAGCAGCAGCAGCAACAGCAACATATTATGGGTCAGAACCAACAGCAAAACCTTATGCAGTCCCAGCTTCCAGATCAAGTCAACCAACATTTACAAATGACTGACAAGCAGATTCAACTTCAACTGTTGCAGAAGCTTCAGCAACAGCAACAGTCATTTTTAGCACAACAGTCTGCACTACAACAACCTGCTCAACTTCCTCAAATCCAAGATCAGCATAGGCAACTATTAGATGTGTCCCAGAGCTTCTCTAGGTCTGTGACATCCACCCAAATGCTGGATTTGCCTCAAGCAACATCTACTTCACTCCCTCAGTCAAACCTTATCTCGCAGCAGATAACAAAGGGTAACAACCTGACAAATGGTCGATTCTCCCATCCTCCCCAGCAGCCAAAGCTTCAGCAGCAGCAACCTGGCATGCTGCCTGAAATGCCTGGACATGTTGGGCTTCCCCCCACCCAAATTACCAATCAACCTTCCACAGCTGGTAGTAATGTTTTGACAGGAGCTGCTGGAGCAGGGCAATCTGTAGTTACTGATGATCTTCCATCTTGTTCCACGTCACCTTCCACAAACAACTGTCAAAATGTTATTCCACCAATGATGCACAATAGGCCCAACCGAAGCACAACAATGGGAGAGGACATGGCTCAGTCTACCACCATTGCCTTGTGCTCAAGTGGTTTAGAAACCATGTCATATAATGGTAACTTAGTTAAAGATTTTCAGCACAAATCTGATGTTAAGCCGTCATTGAACATATCCAGGAATCAAAACCAAGGTTTTTTGGCCCCTCAGACATACTTGAATGGTGCAACCACCCAGACAGATTATTTGGATACATCATCTTCAACAACGTCAGTTTGCCTTTCTCAGAATGATGTCCATTTCCAGCATAATAACAATTCACTGTCGTACCATCCACCATCGATGTTGAGGGATGTGAGCCAAGATGGGGAAGTTCGGGCAGATCCCAGGAGCAGTCTTCCATATGGGGCTAACATTGACAATATTGTGTACAAACGTGGAGCTGTTGGAAGATCAATCGATATTACACGTTATTCAGGTTACGATGAGCTCAAACAAGATCTGGCTCGTAGGTTTGGTATAGAGGGCCAATTGGAAGACCACCAGAGGATAGGCTGGAAACTTGTGTATGTGGATCATGAAAATGATGTCCTTCTAGTTGGGGATGACCCGTGGGAGGAGTTTGTGAGCTGTGTCCGCTGCATCAAGATCTTGTCCCCGCAAGAAGTACAGCAAATGAGCTTGGATGGCGATTTTGGGAATTCTGTCCTTCCAAATCAGGCATGTAGCAGCTCTGATAACGGGAACACGTGA

>LcARF19A

ATGAAGGCTCCAGCAAATGGGTTTTTGGCAAATTCTGCAGAAGGAGATAGGAAAAGTATTAATTCAGAGTTATGGCATGCTTGTGCCGGACCACTGGTTTCTTTGCCTCCGGTTGGAAGTCTGGTGGTTTACTTCCCTCAAGGCCACAGCGAGCAAGTTGCAGCATCGATGCAAAAGGAGAACGACTTCGTACCCAACTACCCCAACCTTCCTTCCAAGTTAATTTGCATGCTTCATAATGTCACATTGCATGCTGATCCAGAAACGGATGAGGTCTACGCGCAGATGACACTTCAGCCTGTAAATAAATATGAAAAGGAAGCGTTACTGGCATCTGATATGGGCCTTAAGCAAAACAGGCAGCCAACGGAGTTTTTCTGCAAAACTCTTACAGCAAGCGACACAAGCACACATGGTGGATTTTCTGTACCTCGTCGAGCAGCAGAGAAGATCTTTCCTCCTCTGGATTATTCGATGCAACCACCTGCTCAAGAGCTATTAGCTAAGGATTTACATGACACTACATGGACATTTAGACATATTTATCGAGGCCAACCAAAAAGGCACCTGCTGACTACTGGATGGAGCGTCTTTGTTAGCACTAAAAGACTATTTGCTGGTGATTCTGTTCTTTTTATAAGGGATGAGAAGTCTCAGCTTCTTTTGGGAATAAGACGTGCTAATAGACAGCAACCAGCTCTCTCTTCATCAGTCATATCCAGTGATAGCATGCATATTGGAATTCTTGCTGCTGCAGCTCATGCTGCTGCAAACAATAGTCCATTTACTATATTTTACAATCCAAGGGCAAGCCCTTCAGAGTTTGTGATTCCCATGGCCAAGTACAATAAAGCAATGTACACACAAGTTTCCCTTGGCATGCGATTTCGAATGATGTTTGAGACTGAGGAGTCAGGAGTGCGCAGATACATGGGTACAATCACTGGCATCAGTGATTTAGATGCTGTGAGATGGAAAGGTTCGCAGTGGCGCAGTCTTCAGGTTGGATGGGATGAATCAACAGCTGGTGAACGGCCAAGTCGAGTTTCTATCTGGGAAATTGAGCCTGTTGTAACTCCTTTCTACATATGCCCTCCCCCTTTTTTCAGACCCAAGTTTCCTAAACAACCAGGGATGCCAGATGATGAATCCGACATAGAGAATGCTTTTAAGCGAGCGATGCCCTGGCTTGGAGATGATTTTGGTATGAAGGATGCACCCAACTCAATCTTCCCTGCTTTGAGTTTAGTTCAGTGGATGAGCATGCAACAGAATAATCAGTTTCCAGCTGCTCAGTCAGGTCTCTTCCCCTCCATGGTTTCTTCTACTGCCATGCATAATAACCTTAGCACTGATGATCCATCTAAGTTGTTGAATTTTCAAGCTTCAGCACTATCTACGCCTACCATCCAGTTTAACAAAGCAAACCCCCAAAACCAAGCCAATCAGTTGCCACAGCCACCTATTGCATGGCCCCAACAACAGCAGCTTCAGCAGTTGCTGCAGACTCCTCTAAGCCAACAGCAGCAGCAACACTTTTTACGTGATTCGAGCCGCAACAACAAACACAACCATTGCCAACTCCACCACAGTCACAATCTCAACCACCATTATCACGACAGCAGCAGCCGCAGCAGCAGCAACAACCACAGCCACATTCGCAAGTATTCCTTCCAGCTACTGTAA

>LcARF19B

ATGAAGGCTCCAGCAAATGGGTTTTTGGCAAATTCTGCAGAAGGAGATAGGAAAAGTATTAATTCAGAGTTATGGCATGCTTGTGCCGGACCACTGGTTTCTTTGCCTCCGGTTGGAAGTCTGGTGGTTTACTTCCCTCAAGGCCACAGCGAGCAAGTTGCAGCATCAATGCAAAAGGAGAACGACTTCATACCCAGCTACCCCAACCTTCCTTCCAAGTTAATTTGCATGCTTCATAATGTCACATTGCATGCTGATCCAGAAACGGATGAGGTCTACGCGCAGATGACACTTCAGCCTGTAAATAAATATGAAAAGGAAGCGTTACTGGCATCTGATATGGGCCTTAAGCAAAACAGGCAACCAACTGAGTTTTTCTGCAAAACTCTTACAGCAAGCGACACAAGCACACATGGTGGATTTTCTGTGCCTCGTCGAGCAGCAGAGAAGATCTTTCCTCCTCTGGATTTTTCGATGCAACCACCTGCTCAAGAGATAGTAGCTAAGGATTTACATGACACTACATGGACATTTAGACATATTTATCGAGGCCAACCAAAAAGGCACCTGCTGACTACTGGATGGAGCGTCTTTGTTAGCACTAAAAGACTATTTGCTGGTGATTCTGTTCTTTTTATAAGGGATGAGAAGTCTCAGCTTCTTTTGGGAATAAGACGTGCTAATAGACAGCAACCAGCTCTCTCTTCATCAGTCATATCCAGTGATAGCATGCATATTGGAATTCTTGCTGCTGCAGCTCATGCTGCTGCAAACAATAGTCCATTTACTATATTTTACAATCCAAGGGCAAGCCCTTCAGAGTTTGTGATTCCCATGGCCAAGTACAATAAAGCAATGTACACACAAGTTTCCCTTGGCATGCGATTTCGAATGATGTTTGAGACTGAGGAGTCAGGAGTGCGCAGATACATGGGTACAATCACCGGCATCAGTGATTTAGATGCTTTGAGATGGAAAGGTTCGCAGTGGCGCAGTCTTCAGGTTGGATGGGATGAATCAACAGCTGGTGAACGGCCAAGTCGAGTTTCTATCTGGGAAATTGAACCTGTTGTAACTCCTTTCTACATATGCCCTCCCCCTTTTTTCAGACCCAAGTTTCCCAAACAACCAGGGATGCCAGATGATGAATCCGACATAGAGAATGCTTTTAAGCGAGCGATGCCCTGGCTTGGAGATGATTTTGGTATGAAGGATGCACCCAACTCAATCTTCCCTGCTTTGAGTTTAGTTCAGTGGATGAGCATGCAACAGAATAATCAGTTTCCAGCTGCTCAGTCAGGTCTCTTCCCCTCCATGGTTTCACCAACGAACTTTCTGACCAGAAACCAGCAAGGACCAGCCATTCTGGCCGGAGATTCAGTGGTTGAACCTGCAAGTAATTTGGTCCAAGAGCTTCACAACAAGTCTGATATCAGGATGAAACATGAGTTGCTTAGCTCGAAGGGACCAGATCATCTGAAATACAAGGGTGGTGTGACTGATCAATTGGAAGCTTCCTCTTCGGGAACATCATATTGCCTTGATCCGAGCAACATCCAGCAGAATTTTTCACTCCCCACCTACTGTTTGGATGGTGATGTCCAATCAAATTCTCGAAACAACGTTCCTTTTGCAGCAAATATGGACGGAATGGCACCTGATACATTATTGTCAAGGGGATATGATTCTCAGAAGGATCTTCAAAACTTGCTCTCTAACTATGGTGGTGCTCCAAGAGATATTGAGACAGAGTTGTCCACTGCTGGAATAAGTTCTCAGTCATTTGGTGTGCCAAACATATCTTTCAAGCCCAGTTGTTCAAATGATGTTGCCATTAATGAAGCTGGGGTTTTGAGCGGTGGATTGTGGGCCAACCAGACGCAACGTATGCGAACATATACAAAGGTTCAAAAGCGCGGGTCTGTGGGAAGATCAATTGATGTTACACGTTACAAAGGTTATGATGAGCTAAGGCATGATCTAGCTCGCATGTTTGGTATTGAGGGACAGTTGGAAGATCCGCAAAGCTCCGACTGGAAGCTGGTTTATGTGGACCATGAAAATGACATATTACTCGTTGGTGATGATCCTTGGGAGGAGTTTGTCAGCTGCGTGCAGAGCATAAAGATACTTTCATCAGCTGAAGTACAGCAGATGAGCTTAGACGGAGACCTTGGTAATAATGCAAATCTGCCGGTTCCAAATCAAGCTTGTAGTGGGACTGACAGCGGGAATGCATGGAGAGCACAGTATGATGATAACTCAGCTACCTCATTTCGATGA

>LcARF5A

ATGAAAACAGGAGACTTGGTTGGGGTTGGTCGAGCACAAACAACTTTGCTCGAGGAAATGAAGCTGTTGAAAGAAATGCAGGATCAGTCTGGAACCCGGAAAACCATAAATTCCGAGCTATGGCATGCCTGTGCTGGTCCTCTTGTTTCGTTGCCACAGGTGGGAAGTCTTGTGTACTACTTCCCTCAAGGACATAGTGAACAGGTGGCAGTTTCCACTAAAAGGACAGCGACGACCCAAATTCCAAACTATCCTAATCTTCCATCTCAGCTATTGTGCCAAGTTCACTATGTTACATTACATGCAGACAAAGAGACTGATGAAATTTATGCCCAAATGAGTCTTCAGCCGGTGAACTCTGAGAAGGATGTCTTCCCCATACCAGACTTTGGACTCAAGCCAAGCAAACACCCAAATGAGTTTTTCTGCAAAACCCTGACTGCAAGCGATACAAGCACACATGGTGGTTTCTCAGTGCCACGCAGGGCAGCAGAAAAGCTCTTTCCTCCATTGGATTACACAATGCAACCACCCTCTCAAGAGCTTGTTGTCCGGGACTTGCACGATAATACCTGGACATTTCGCCATATATATCGTGGGCAGCCCAAGAGACACCTTCTCACAACAGGGTGGAGTTTGTTTGTTGGGTCAAAAAGGCTTAGAGCAGGTGATGCTGTTTTGTTTATCAGGGATGAGAAGTCACAGTTGTTGGTGGGTGTAAGGCGCGCAAATCGTCAACAAACAGCATTACCCTCGTCAGTTTTATCTGCTGATAGCATGCACATTGGTGTCCTTGCTGCTGCAGCTCATGCTGCCTCCAATAGAACCTCATTTACAATTTTCTACAATCCTAGGGCATGTCCGTCAGAATTTGTTATTCCTTTGGCAAAATATCGTAAGTCTGTTTATGGGACTCAAATCTCAGTTGGTATGCGGTTTGGGATGATGTTTGAAACAGAGGAGTCTGGTAAACGTAGATATATGGGTACGATAGTTGGTATTAGTGACTTGGATCCATTGAGGTGGCCTGGTTCCAAGTGGCGAAATCTTCAGGTGGAATGGGATGAACCGGGGTGTAGTGATAAGCAGAAAAGGGTTAGTTCATGGGAAATTGAGACTCCTGAAAGTCTCTTCATTTTTCCTTCTCTGACTTCTGGACTCAAACGACCATTTCATCCTGGATTATTAGGAGCAGAAGTTGAATGGGGAAATTTGATGAAGAGGCCCCTTCCCCGTCCTCCTGAAATTGGAAATGGGGCTATTCCCTACTCTTCAATTTCAAACTTGTGTTCTGAGCAATTGCTTAGGATGATGCTAAGACCTCAACTCGTTAACCATTCTGGGACCTTCACATCTTCCCTTTCGCAAACCTCTGCTGTTAAAGGAACTTTACCTATTGTATTTTCATTTGCAAGTCAGGTGGAATGGGATGAACCGGGGTGTAGTGATAAGCAGAAAAGGGTTAGTTCATGGGAAATTGAGACTCCTGAAAGTCTCTTCATTTTCCCTTCTCTGACTTCTGGACTCAAACGACCATTTCATCCTGGATTATTAGGAGCAGAAGTTGAATGGGGAAATTTGTTGAAGAGGCCCCTTCCCCGTCCTCCTGAAATTGGAAACGGGGTTATTCCCTACTCTTCAATTTCAAACTTGTGTTCTGAGCAATTGCTTAGGATGATGCTAAGACCTCCACTCGTTAACCATTCTGGGACCTTTGCATCTTCCCTTCCGCAAACCTCTGCTGTTAAAGGAACTCCACTGGAAGAGGTTAAGACCTTGCAGGCTACGGTCAATCAGAAACCCCAACTTATCCAGTCAGAAAATACAATCATAGAAAGCCAAAATTGTTTCCAGTCCGGCCTCGACCAAGCTGATACCATAAATTCAAGTTCATCAAAAATAAATTTACCTGAAAGGCCAAATCCTTCAAGCAAATGTGATAAGCAAACACCAGCTGGAACTAATATTGATAGTTTAAAATCAGAGCCTGAGCAATCAACACATCAGTTGAGCCATTTAACTTCAACGGCAGAGTGCAGTGAGGAGAAGCTGGTCTCTAGTCCTCTAAATCCACAGACCATTTTAAACCAGCTTATGTTACAAAACCAGAACCAGGGTCTAATGCAATTGCAATCTAGTATGTGGCCTATTCAGTCACCGTTGGAGTCATCAGTATTTCAGGCACAACAGGTTAACTTACCACAATCTGATTCTGCTAATTTAAACGGCTTACTTCCTTTCTCAGATGCTGAGGAATGGATATATAATAAAGTATCTGGGCCTCTATCAATGTATGGTTTGCAAGACCCTTCAACAGCATTCCCTGAAGTGATTAATCCGCCTCTACCTTCCACAGGTCAGGATATGTGGGATCATCAACTGAATAATTTAAAATTCTTATCCCAAGTTGACCAGTTCACTCCATTTGCTCAGCAAGATATGTCTAACCTCAATTCTAATGGATTGAGAGATTTGTCAGACGAAAGCAATAATCAAAGTGGGATCTACAGCTGTCTTAATGTTGATGTTAGTAATGGAGGGGGTACTGTGATTGATCATTCAGTTTCTAGTGCCATTCTGGATGACTTTTGTACATTGAAGGATGCCAATTTCCAGAATCCTACAGATTGTTTGATGAACAACTTTAGTTCTAGCCAGGATGTTCAGTCTCAGATTACTTCTGTAAGTTTAGCTGACTCGCAGGCTTTCTCTCGTCAAGATTTCCCTGACAACTCAGGTGGCACATCATCAAGCAATGTGGATTTTGATGAAAACAGTCTATTGCAGAAGACCTCGTGGCAGCAAGTAGCACCACCTGTGCGGACGTATACAAAGGTTCAAAAAGCAGGATCTGTTGGGAGGTCGATTGATGTCACAACTTTTAAAAATTATGAAGAGCTACGCTCTGCAATTGAACGCATGTTCGGACTTGAGGGGTTGCTTACGGACCCCAGAGGTTCTGAGTGGAAACTGGTGTATGTGGATTATGAAAATGATGTTCTGCTTGTTGGGGATGACCCTTGGGAGGAATTTGTCGGCTGCGTCCGCTGCATCAGGATTCTGTCGCCTCAAGAAGTTCAGCAGATGAGTGAAGAGGGAATGAAGCTTCTTAACAGTGCTGCAATGCAAGGCATTGATTGTTCCATGGCAGGAGGTGACCGCGCCTGA

>LcARF5B

ATGAAAACAGGAGACTTGGTTGGGGTTGGTCGAGCACAAACAACTTTGCTCGAGGAAATGAAGCTGTTGAAAGAAATGCAGGATCAGTCTGGAACCCGGAAAACCATAAATTCCGAGCTATGGCATGCCTGTGCTGGTCCTCTTGTTTCGTTGCCACAGGTGGGAAGTCTTGTGTACTACTTCCCTCAAGGACATAGTGAACAGGTGGCAGTTTCCACTAAAAGGACAGCGACGACCCAAATTCCAAACTATCCTAATCTTCCATCTCAGCTATTGTGCCAAGTTCACTATGTTACATTACATGCAGACAAAGACACTGATGAAATTTATGCCCAAATGAGTCTCCAGCCGGTGAACTCTGAGAAGGATGTCTTCCCCATACCAGACTTTGGACTCAAGCCAAGCAAACACCCAAATGAGTTTTTCTGCAAAACCCTGACTGCAAGCGATACAAGCACACATGGTGGTTTCTCAGTGCCACGCAGGGCAGCAGAAAAGCTCTTTCCTCCATTGGATTACACAATGCAACCACCCACTCAAGAGCTTGTTGTTCGGGATTTGCACGATAATACCTGGACATTTCGCCATATATATCGTGGGCAGCCCAAGAGACACCTTCTCACAACAGGGTGGAGTTTGTTTGTTGGGTCAAAAAGGCTTAGAGCAGGTGATGCTGTTTTGTTTATCAGGGATGAGAAGTCACAGTTGTTGGTGGGTGTAAGGCGCGCAAATCGTCAACAAACAGCATTACCCTCGTCAGTTTTATCTGCTGATAGCATGCACATTGGTGTCCTTGCTGCTGCAGCTCATGCTGCCTCCAATAGAACCTCATTTACAATTTTCTACAATCCTAGGGCATGTCCGTCAGAATTTGTTATTCCTTTGGCAAAATATCGTAAGTCTGTTTATGGGACTCAAATCTCAGTTGGTATGCGGTTTGGGATGATGTTTGAAACAGAGGAGTCTGGTAAACGTAGATATATGGGTACGATAGTTGGTATTAGTGACTTGGATCCATTGAGGTGGCCTGGTTCCAAGTGGCGAAATCTTCAGGTGGAATGGGATGAACCGGGGTGTAGTGATAAGCAGAAAAGGGTTAGTTCATGGGAAATTGAGACTCCTGAAAGTCTCTTCATTTTCCCTTCTCTGACTTCTGGACTCAAACGACCATTTCATCCTGGATTATTAGGAGCAGAAGTTGAATGGGGAAATTTGTTGAAGAGGCCCCTTCCCCGTCCTCCTGAAATTGGAAACGGGGTTATTCCCTACTCTTCAATTTCAAACTTGTGTTCTGAGCAATTGCTTAGGATGATGCTAAGACCTCCACTCGTTAACCATTCTGGGACCTTTGCATCTTCCCTTCCGCAAACCTCTGCTGTTAAAGGAACTCCACTGGAAGAGGTTAAGACCTTGCAGGCTACGGTCAATCAGAAACCCCAACTTATCCAGTCAGAAAATACAATCATAGAAAGCCAAAATTGTTTCCAGTCCGGCCTCGACCAAGCTGATACCATAAATTCAAGTTCATCAAAAATAAATTTACCTGAAAGGCCAAATCCTTCAAGCAAATGTGATAAGCAAACACCAGCTGGAACTAATATTGATAGTTTAAAATCAGAGCCTGAGCAATCAACACATCAGTTGAGCCATTTAACTTCAACGGCAGAGTGCAGTGAGGAGAAGCTGGTCTCTAGTCCTCTAAATCCACAGACCATTTTAAACCAGCTTATGTTACAAAACCAGAACCAGGGTCTAATGCAATTGCAATCTAGTATGTGGCCTATTCAGTCACCATTGGAATCAACAGTATTTCAGGCACAACAGGTTAACTTACCACAATCTGATTCTGCTAATCTAAACGGCTTACTTCCTTTCTCAGATGCTGAGGAATGGATTTATAATAAAGTATCTGGGCCTCTATCAATGTATGGTTTGCAAGACCCTTCAACAGCATTCCCCGAAGTGATTAATCCACCTCTACCTTCCACGGGTCAGGATATGTGGGATCATCAACTGAATAATTTAAAATTCTTATCCCAAGTTGACCAGTTCACTCCATTTGCCCAGCAAGATACATCTAACCTCAATTCTAATGGATTGAGAGATTTGTCAGACGAAAGCAATAATCAAAGTGGGATCTACAGCTGTCTTAATGTTGATGTTAGTAATGGAGGGGGAGCTGTGATTGATCATTCAGTTTCAAGTGCCATTCTGGATGACTTTTGTACATTGAAGGATGCCAATTTCCAGAATCCTACAGATTGTTTGATGAACAACTTTAGTTCTAGCCAGGATGTTCAGTCTCAGATTACTTCTGTAAGTTTAGCTGACTCGCAGGCTTTCTCTCGTCAAGATTTCCCTGACAACTCAGGTGGCACATCATCAAGCAATGTGGATTTTGATGAAAACAGTCTATTGCAGAAGACCTCGTGGCAGCAAGTAGCACCACCTGTGCGGACGTATACAAAGGTTCAAAAAGCAGGATCTGTTGGGAGGTCGATTGATGTCACAACTTTTAAAAATTATGAAGAACTACGCTCTGCAATTGAACGCATGTTCGGACTTGAGGGGTTGCTTACGGACCCCAGAGGTTCTGAGTGGAAACTGGTGTATGTGGATTATGAAAATGATGTTCTGCTTGTTGGGGATGACCCTTGGGAGGAATTTGTCGGCTGTGTCCGCTGCATCAGGATTCTGTCGCCTCAAGAAGTTCAGCAGATGAGTGAAGAGGGAATGAAGCTTCTTAACAGCGCTGCAATGCAAGGCATTGATTGTTCCATGGCAGGAGGTGACCGCGCCTGA

>LcARF3A

ATGGTGGGTCTAATCGATCTCAACACGACAGAGGACGACGTCGACGAGACTCAGACTCAGGCTCAGACTCCCTGCTCTGGGTCTCTCTCTCCTTCCTCTTCCACTGCTTCTGGTGCTCCGGCTTCGGCTTCTGGGGTGTCTTTGGAGCTCTGGCACGCGTGTGCAGGTCCACTCATATCTCTGCCCAAGAGAAGCAGCCTGGTCGTGTACTTTCCACAGGGACATTTGGAACATGTCTCCGATTTTTCCGCCTCTTCTTCCGCATATGATCTCCCTCCCCACTTGTTTTGTCGGGTTCTCGATGTCAAGCTTCACGCTGAGGCGGCAAGTGATGAGGTCTATGCTCAGGTCTCGCTGCTTCCTGAAAATGAGCAAACTGAGCAGAAGCTGCGAGAAGGGAACATTGATGTGGAAGGTGAAGAGGAGGATGTTGAGGTCACTGGTAAGGCTTCAACACCCCATATGTTTTGCAAGACCCTTACTGCTTCTGATACCAGCACTCATGGAGGTTTCTCCGTACCTCGTCGAGCTGCGGAGGACTGCTTCCCTCCCCTGGATTATACTCAACAAAGGCCTTCACAGGAACTTGTGGCAAAGGATCTGCATGGCCTGGAATGGAGATTTCGACACATCTACCGGGGGCAACCACGGAGGCACTTGCTAACTACTGGATGGAGTGCATTTGTTAATAAGAAGAAGCTTGTATCTGGAGATGCTGTGCTTTTCCTGAGGGGTGAAGATGGTGAGCTGAGACTGGGAATCCGAAGAGCAGCTCAAGTAAAAGGTGCTGCTACATTTCCACCTGTCTCTAGCCAGCAGTTGAGTCACACTACCGTCTCAGATGTGGCTAATGCTATGTCTATGAGACGTGCATTCAGCATTTGCTACAATCCAAGGGCCAGTGCATCAGAGTTCATAATACCTGTTCATAAGTTCTTGAAGAGCCTAGACCATACTTTTCAAGTTGGAATGAGGTTCAGAATGCGATTTGAAACTGAAGATGCAGCAGAACGAAGATACACAGGACTGATTACGGGCATTGCCGACATGGATCCTGTTAGATGGCCTGGTTCAAAATGGAGATGTCTATTGGTGAGGTGGGATGATGTTGAGGCTAACAGGCACAGCAGGGTTTCTCCATGGGAAATTGAGGCATCTGGTTCAGTTGCTAGTTCCAATAGCCTTATCTCACCTGGTTTGAAGAGGACCAGGATTGGATTGCCTCCAGGGAAACCAGAATTTCCAGTTCCTGATGGGATTGGAGTATCAGACTTTGGGGAATCTTTGAGGTTCCAGAAGGTCTTGCAAGGTCAAGAAATTTTGGGTTTTAATACTCTTTATGATGGTGTTGATAGTCGAAATCTACATCCATCTGACATGAGGAGGTGCCTTCCTGGTTCAAATAGATCTGGGATTGCTGCAATAGGAGATGGCATGAGAAACCCTCATGTGAATTCTGATGTTTCCTATAAAAGCATAGGCTTTGGTGAATCCATCCGATTCCATAAGGTCTTGCAAGGTCAAGAAATATATCCAAAGTCCCCATATGGAAGGGCCCCAACTACTGATGAGGCTCGTGGAAATGGTGGCCTTGGAACCTCTGATGGTGTTCAGGTGCCAGGGTCGAGAAACGGATGGTCTGCCATGATGCAGGGATATAATACTCATACCAGTCCTCCTGCACCATCCGTTCAAGTCTCATCACCATCTTCAGTTTTAATGTTCCAGCTAGCAAGCAATCCAGTTTCAAACTATAATCCTGTATATAGCTTGAATGATCAGGAAAAGGGGCAACAAATTAGCAACCATAGTTTGTTTCATACTGCAGGAATATATGGGGGAAAGCATGCCTCATCGTCACTTGGTGGGCATAGTTTCATGGGGGAGGATCAGGGATGCATGAATTCTTTGGGCCATTCAAATGAGCTTATCCAACTCGGTATGTCACGACCTCTTGTAGTTCAACCGGCATTTAGGGGCAGTCAAGATTTAGTTTCTTCATGTAAAAGTAGCTGCAGACTATTTGGTTTTTCTTTGACTGAGGAGAGACATGTTGCTGCCAATATAGACAACAGCAGAGCTCCAGTTCCATCTCCTCTAACTCCTGGAGCTGCTTTCGTGCCTCGTGTTGGAGAGCAGTTCCGTCCAAAGCCTCCAGCAATGTCCACACCCGTTGGAAACAACTGCTCCAATGTAAGCCACTTCTATGCCGTAAAAGATATGCTTTCTGATATTGCATTGTAG

>LcARF3B

ATGGTGGGTCTAATCGATCTCAACACGACAGAGGACGACGTCGACGAGACTCAGACTCAGACTCCCTCCTCTGGGTCTCTCTCTCCTTCCTCTTCCACTGCTTCTGGTGCTCCGGCTTCGGCTTCTGGGGTGTCTTTGGAGCTCTGGCACGCGTGTGCAGGTCCACTCATATCTCTGCCCAAGAGAAGTAGCCTGGTCGTGTACTTTCCACAGGGTCATTTGGAACATGTCTCCGATTTTTCCGCCTCTTCTTCCGCATATGATCTCCCTCCCCACTTGTTTTGTCGGGTTCTCGATGTCAAGCTTCACGCTGAGGCGGCAAGTGATGAGGTCTATGCTCAGGTCTCGCTGCTTCCTGAAAATGAGCAAACTGAGCAGAAGCTGCGAGAAGGGAACATTGATGTGGAAGGTGAAGAGGAGGATGTTGAGGTCACTGGTAAGGCTTCAACACCCCATATGTTTTGCAAGACCCTTACTGCTTCTGATACCAGCACTCATGGAGGTTTCTCCGTACCTCGTCGAGCTGCGGAGGACTGCTTCCCTCCCCTGGATTATACTCAACAAAGGCCTTCACAGGAACTTGTGGCAAAGGATCTGCATGGCCTGGAATGGAGATTCCGACACATCTACCGGGGGCAACCACGGAGGCACTTGCTAACTACTGGATGGAGTGCATTTGTTAATAAGAAGAAGCTTGTATCTGGAGATGCTGTGCTTTTCCTGAGGGGTGAAGATGGTGAGCTGAGACTGGGAATCCGAAGAGCAGCTCAAGTAAAAGGTGCTGCTACATTTCCACCTGTCTCTAGCCAGCAGTTGAGTAACACTACCGTCTCAGATGTGGCTAATGCTATATCTATGAGACGTGCATTCAGCATTTGCTACAATCCAAGGGCCAGTGCATCAGAGTTCATAATACCTGTTCATAAGTTCTTGAAGAGCCTAGACCATACTTTTCAAGTTGGAATGAGGTTTAGAATGCGATTTGAAACTGAAGATGCAGCAGAACGAAGATACACAGGACTAATTACGGGCATTGCCGACATGGATCCTGTTAGATGGCCTGGTTCAAAATGGAGATGTCTATTGGTGAGGTGGGATGATGTTGAGGCTAACAGGCACAGCAGGGTTTCTCCATGGGAAATTGAGGCATCTGGTTCAGTTGCTAGTTCCAATAGCCTTATCTCACCTGGTTTGAAGAGGACCAGGATTGGATTGCCTCCAGGGAAACCAGAATTTCCAGTTCCTGATGGGATTGGAGTATCAGACTTTGGGGAATCTTTGAGGTTCCAGAAGGTCTTGCAAGGTCAAGAAATTTTGGGTTTTAATACTCTTTATGATGGTATTGATAGTCGAAATCTACATCCATCTGACATGAGGAGGTGCCTTCCTCGTTCAAATAGTTCTGGGATTGCTGCAATAGGAGATGGCATGAGAAACCCTCATGTGAATTCTGATGTTTCCTATAAAAGCATAGGCTTTGGTGAATCCATCCGATTCCATAAGGTCTTGCAAGGTCAAGAAATATATCCAAAGTCCCCATATGGAAGGGCCCCAACTACTAATGAGGCTCGTGGAAATGGTGGCCTTGGAATCTCTGATGGTGTTCAGGTGCCAGGGTCGAGAAACGGATGGTCTGCCATGATGCAGGGATATAATACTCATACCAGTCCTCCTGCACCATCCGTTCAAGTCTCATCACCATCTTCAGTTTTAATGTTCCAGCTAGCAAGCAATCCAGTTTCAAACTATAATCCTGTATATAGCTTGAATGATCAGGAAAAGGGGCAACAAATTAGCAAACATAGTTTGTTTCATACTGCAGGAATATATGGGGGAAAGCATGCCTCATCGTCACTTGGTGGGCATAGTTTCATGGGGGAGGATCAGGGATGCATGAATTCTTTGGGCCATTCAAATGAGCTTATCCAACTCGGTATGTCACGACCTCTTGTAGTTCAACCGGCATTTAGGGGCAGTCAAGATTTAGTTTCTTCATGTAAAAGTAGCTGCAGACTATTTGGTTTTTCTTTGACTGAGGAGAGACATGTTGCTGCCAATATAGACAACAGCAGAGCTCCAGTTCCATCTCCTCTAACTCCTGGAGCTGCTTTCGTGCCTCGTGTTGGAGAGCAGTTCTGTCCAAAGCCTCCAGCAATGTCCACACCAGTTGGAAACAACTGCTCCAATGTAAGCCACTTCTATGCTGTAAAAGATATGCTTTCTGATATTGCATTGTAG

>LcARF4A

ATGGAGATCAATCCTGAACTTACAACAACCCAGTCAAAGAAGAAAGATGCAGAGACTGAGGTCGAAAACAAAGATTTCACTGCCGTCTTCATGGAGAATAAGGAAGATTTCACAGCAGTCTTCGACGAGTATAGTGGTGGTGTCTATGCAGAGACCGAGGCCAAAAACAAAGGTTTCACTGCAGTCTCTATGGAGAATAAGGAAGATCTGACAACATTCTTTGACGAGGATAGCGATTATAACTTGGACTTATGGCGTGCTCTAGTCGGCAATCGGCCTTCACTGCCCAAGAAAGACGACTTGGTTGTCTACTTCCCACAAGATCATTTGGAATATGCTGCCTTAATTACTTCCTCCTCATTCTCATCCTCAGAAGTCCCTACCTTCAATCTCCAACCAGAGATCATCTGCAGCGTTCAACATGTCCAATTAATTGCCAATAAGATGACTGATGAAACATGTGCACATGTTACTTTGCTTCCAGAACAAAAGTTGGAAGGAACAAATCCAAAGGGCGAAGAGGTTATGAAGTCAGGTGTAAGTAAGGCAAAAGACAGAAGAAGACCTACAAAAGCAGCCTTGCATGTGTTCCCTGTCACACTCTCTAAACCTGAGGATTTACAAACTATATCGATACCCGTGGATGCTTATGAAGTCTTTCGTTTACGGAACTTTAGGTTTAGTACCCAGGTGGTTTCCGCTGAGGATGTTCAAGGGACAGTGTGGATATTTTATATTGAATATGCAGACTCGCCAAGAAAGTACCAGATTACGAGGGGATGGACCGAATTTTTGAGAAGAAAGAGGCTTAGTTTAAAAGATACAGTGGCATTTGTTAGCTGTGGAGATAGGAAATTGAGGTTGGTAATAAAAAGATTTGGCTGTGAATGTGGTGTTCCTATGTCCATCCTTGCAAAGTACTATCAATGTTCCAGTGTCCTTTCTCCAGTGGCTGATGCAATACTCAAAGGGAGCCCGTTTAACGTTTTTTATTGTGGAAGGGTCGGTAGTGCAGATTTTATAATACCTTACCAAAAGTACATGGAAAGCATTACAAGACTGGGGATGATTGGGGCCAAATTCGAGACCATGGATGCTCTTTCACAGAGATGTAGTGGTGTAGTGACAGGAATTTCGGAGTTGGATCCCTTTAATTGGCCTAACTCAAAATGGAAATGCTTGATGGTGAGCTGGGATAATGCTGCTAATAAGAAACCTCTAAAAGAAGCAGTTTCCCCGTGGGAAATAGTTCTTAAAGTTCCCTCATCCTCAGATTCAAGTATTGATATTTCACCCAAGTTGGAGAAGCCACGGACTGTTAGACTGCTTAACATATCTCTTCCTCTTCATGATCCTGCAGAGAGGGAGAAACAGGCCAGTGTGATGGGACTAGAAGGGTTTCAACGGAAAATAAACCACGTCATTGATATTATAGAACAAGCTCCAGCACCCCAAACTGCCTTCCCAAAAGCACTCTCTTCTGATAAACTCCAGAGGAATTCAAGTCCTAAAGCCTGGGAAAAACTTAACACATCTCTTTATGATCCTGCAGAAAGGGAGAAACAGGCCAGTGCAATGGTACAAGAAGAGTTTCAACGAAAAGAGTTTCCATTAAAGCGCATCGTGGATATTATTAAGCAAGGTCCAGCACCCCAAACAGCGTTCTTAGAAGCACTCTCTTCTGATAAACTCCAGCGGAATTCAAGATCTAGAGCCTGGAACAAGCTTAACACATCTTTTTATGATCCTGCAGGAACCTCGTTTGTGTTATTTGACGACAAATTAGAAGAAAGGGGGAAACAGGCCAGTGTGATGGTACAAGAAGAGTTTCAACAAAAAGAGTTTCCATTAAAGCGCATCGTGGATATTATAAAGCAAGGTCCAGCACCCCAAACAGCGTTCTCAGAAGCACTCTCTTCTGATAAACTCCAGCGGAATTCAAGATCTAGAGCCTGGAACAAGCTTAACACATCTTTTTATGATCCTGCAGGAACCTCGTTTGTGTTATTTGACGACAAATTAGAAGAAAGGGGGAAACAGGCCAGTGTGATGGTACAAGAAGAGTTTCAACGAAAAGAGTTTCCATTAAAGCGCATCGTGGATATTATAAAGCAAGGTCGAGCACCCCAAATAGCCTTCCCAAAAGCACTCTCTTCTGATGAACTCCAGAGGAATTCAAGACCTAGAGCCCGGAAAAAGCGTAGTGGTTTTGATACTGATAGAGACTCTTCTTTGGCATCTTCGAAAAAACGGAAGTCAGCCACAATTGTTACGCCTACACATTTTAAGATCAGTAAACGTCCAGATCAAGAGCTAATGATTTGTTTTGAAAGAGGTGTATCTAAGGCCACTCATGATAAAGTTTCTGTAGATAGACTCAAGCCTCTTCAAATGCAATTCTCGCATGCCCAACAAATCTGCGAATCTACTGCTCCCTTTCCTGATAAAGGTGATGTCTCCTCATCTATTATGACTCAGCCGTCATCAAGGTTGAAGAGACAGCGGAAATTAGACAGTTCACCTGTTGACATTGAAACTGAAGGTTTTCTAGATGAACTCAAGCCTCTTGAAATGCAATCCCCGGAATCCAAACAAGTCCTGCCATCTACCACTCCCTTTCCTGGTAAGGGTGATGTCTCCTCATCCATTATGGCCCAGCCGTCATCAAGGTTGAAGAAGGAACTGGAAAACTCACCTCTTGACTCCTTCCGATTCACACCCCTTACTGGTAACTGTTGTTTGGCATCTTTGTCAGAAAGCAAGTCAACCACTGATGTTAAACAGCTGAAGATCAGTAACTGTTCAGGGCATGACGTCTGGTTTCACCTGAAAGGAGACCATTGTTTTGAATACTTGGAGTTCGATTCTTGTTCTTTTCCCTCGTCCACAAGTCTACATATTCCTATCATTCTTAAAGAATTGAAGATCACTAACTGCCTAGTTACGGAATGTGTATTTACGTCGTTGATGGACCCAAAAGTGTCAGTCGAATCCTTGGAGATAGATAGTTGTTCTTCTCTCCATTCCATTTCAATAGACAATATTCCTGCCAGTCTTAGACAACTGAAAATAGTAAATTGCATGAATTTGAAGTCACTTAGTGAATCAATATTTGAGAGCAGCAAGCATCATTCTGTCAGTTTAGGTCCTCTAAATTATTCAGACTTATCAAAGATTGACTCGATTGGCACTCTGGAGCTTAGAAAGTTAGTGATTCAGAATTGTCTGGAGCTTGAGTACTTGCCTGAGGACCTCCACAAACTCTGCAATCTGGATGTACTGTCAATAAAGAATTGTTGTAGTCTAGAGTATTTTCCAGAAGGTGGTTTGCCCAAAACCTGTTTAAGATCACTTAAGATTTCTGAATGTGAAAATCTTAAGCGTCTTCCAAATCAATTAGAGGAGGTCACATCGTTGCAGAAATTAAGCATTTGTGGTTGCTCATCTCTTGTGTCATTTCAAGAGGGAGGTTTGCCCCCTAATCTAGTTTCTCTCCGCATCATCAATTGTGAAAACTTTTTTCTAGATCTATCTCAATGGGATCTCGATAAACTCAAGCATCTTAAGCTATGCTCCTTTATTGGTGGATGTCCAGATTCTTAG

>LcARF4B

ATGGAAATTGATCTGAACCATGCATTTTGCAATGGGGATTGTGAGAAGAACAGTGGCTGTGTTTATTGCTTGTCCTCCTCCTCCTCTTCTTCTTGTTCTTCAAATTCAGGCTCTTCTCCTTTGTCTTCTTCTATCTACTTAGAGCTTTGGCATGCCTGTGCTGGTCCTCTCACTTCACTCCCCAAGAAAGGAAATGTAGTTGTCTACTTCCCACAAGGTCACCTGGAGCAACTGGCCTCTTCCTCTCCTTATCCACCTCTTGAAGGTTCCAACTTCGATCTTCAACCTCAAATCTTTTGCAAAGTTGTGGATGTCCAACTACTTGCTAATAAGGAGAATGACGAGGTCTATACACAGGTCACTTTGCTTCCTCAACCAGAGTTGGTAGGACCAAATTCAGAGGGCAAAGATCTTGAGGAGTTGAGTGTGGATGAGGATGGGAATGGAGGATCACCAACAAAATCAACCCCTCATATGTTCTGCAAAACGCTTACAGCTTCTGATACCAGCACACATGGAGGGTTCTCTGTGCCTCGTAGAGCTGCCGAAGATTGTTTCCCTCCGCTGGACTATAAACAGCAGAGGCCTTCACAAGAGCTTGTTGCCAAGGATCTGCATGGAGTGGAGTGGAGATTTCGGCATATTTATAGAGGCCAGCCAAGGCGACATCTGCTTACTACAGGATGGAGTATCTTTGTGAGCCAAAAGAATCTTGTTTCAGGAGATGCAGTGCTGTTTTTGAGGGGTGACAATGGAGAGCTGAGGTTGGGAATTAGACGAGCTGTTCGACCTAGAAACGGTCTTCCTGAATCTGTTCTTCAGAATTCCTATCCAAATGCTCTTTCACTAGTGGCTAATGCTATATCAACCAAGAGCATGTTTCATGTTTTCTACAGTCCAAGGGCCAGTCATGCAGAGTTTGTCGTACCATACCAAAAGTATGTGAAAAGCATCACGAATCCAGTATGCGTTGGCACCAGATTCAAAATGAGATTTGAAGTGGATGATTCACCAGACAGGAGGTGTAATGGTGTAGTAACTGGAATGAGTGACTTGGATCCCTATCGATGGCCAAACTCAAAATGGAGGTGCTTCATGGTCAGGTGGGATGAAGATATCGGGAGTGACCATCAAGAAAGAGTATCCCCATGGGAAATCGACCGGTCTGTTTCTCTCCCACCCTTGAACATTCAGTCCTCCCCAAGGCTGAAGAAACTGCGGACAAGTCTGCAGGCTACCCCACCTGATTACCCTGTCGCTGCAGGAGGCGGTGGGTTTTTGGACTTTGAGGATTCTATAAGAACCTCTAAGGTCTTGCAAGGTCAAGAAAATGTAGGTTTTGTATCATCCCTTTATGGACGTGATACGGCTAACCGCCCGCTGGATTTTGAGATGCGAGCCCCCATACATCAAAATCTTGCATTACCTAGATTAGAGAAAGGTAATGTTACTAAGTTTGTGGCTCGCCCCACTACTTACACAGGCTTTATGGAATCCGATAGGTTTCCAAAGGTCTTGCAAGGTCAAGAAATATGCCCATTGAGATCCCTGGCCGGAAAAGTTGATCTCAATCTAGGTGCTTGGGGTAAACCCAATCTTGGTTGCAACTCTTTCAACATGTATCCAGCAGCCAAGCCTAGTTTCTACCCACTACCATCAGAAAACCTTCGAACTATGTACTTTCCATATAATGACATGTACAAAAATGGTCAAGATCCCACAATGCGTGCTTATGCTACTAGTTTCCAGAGAGAAAATGCCCAATTTAACTCACCTTCTATTCAGAAGAGGGTTGTTGGGGATGAAGTTAGAAAGCCTATTCTACTGAACGAGCATAAACCAGCTGACAGTATTCCTACTCCCACTTTTAAGACAACTTTGAGAAACCAAAAAGAGGACACCTTTAGTGGAACAGTGGCTGGATGTAAACTTTTTGGATTCTCATTGACTGGGGAAACACCAAGTCCAAGCTCGCAAAACTCTGGTAAGAGAAGTTGTACGAAGGTTCACAAGCAAGGCAGCTTGGTTGGAAGAGCTATTGATCTCTCAAGACTGAATGGTTACAATGACTTGCTGACTGAACTGGAACAACTATTTAGCATGGAAGGCCTTCTACGAGATCCCGATAAAGGATGGCGGATCTTGTACACTGACAGCGAGAATGATGTGATGGTTGTTGGAGATGACCCTTGGCATGAATTCTGCAATGTGGTGTCCAAGATTCATATATACACCAAAGAAGAAGTGGAGAAGATGACCATTGGAATGATCACTGATGACACTCAAAGCTGTTTGGATCAAGCACCGGTGATAATGGAAGCTTCCAAGTCATCCTCAGTAGGCCAGCCGGATTCTTCTCCAACGGTAATAAGGATTTGA

>LcARF4C

ATGGAAATTGATCTGAACCATGCATTTTGCAATGGGGATTGTGAGAAGAACAGTGGCTGTGTTTATTGCTTGTCCTCCTCCTCCTCTTCTTCTTGTTCTTCAAATTCAGGCTCTTCTCCTTTGTCTTCTTCTATCTACTTGGAACTTTGGCATGCCTGTGCTGGTCCTCTCACTTCACTCCCCAAGAAAGGAAATGTAGTTGTCTACTTCCCACAAGGTCACCTGGAGCAACTGGCCTCTTCCTCTCCTTATCCACCTCTTGAAGGTTCCAACTTCGATCTTCAACCTCAAATCTTTTGCAAAGTTGTGGATGTCCAACTACTTGCTAATAAAGAGAATGATGAGGTCTATACACAGGTCACTTTGCTTCCTCAACCAGAGTTGGTAGGACCAAATTCAGAGGGCAAAGAGCTTGAGGAGTTGAGTGTGGATGAGGATGGGAATGGAGGATCACCAACAAAATCAACCCCTCATATGTTCTGCAAAACGCTTACGGCTTCTGATACCAGCACACATGGAGGGTTCTCTGTGCCTCGTAGAGCTGCCGAAGATTGTTTCCCTCCGCTGGACTATAAACAGCAGAGGCCTTCACAAGAGCTTGTTGCCAAGGATCTGCATGGAGTGGAGTGGAGATTTCGGCATATTTATAGAGGCCAGCCAAGGCGACATCTGCTTACTACAGGATGGAGTATCTTTGTGAGCCAAAAGAATCTTGTTTCAGGAGATGCAGTGCTGTTTTTGAGGGGCGACAATGGAGAGCTGAGGTTGGGAATTAGACGAGCTGTTCGACCTAGAAACGGTCTTCCTGAATCTGTTCTTCAGAATTCCTATCCAAATGCTCTTTCACTAGTGGCTAATGCTATATCAACCAAGAGCATGTTTCATGTTTTCTACAGTCCAAGGGCCAGTCATGCAGAGTTTGTCGTACCATACCAAAAGTATGTGAAAAGCATCACGAATCCAGTATGCGTTGGCACCAGATTCAAAATGAGATTTGAAGTGGATGATTCACCAGACAGGAGGTGTAATGGTGTAGTAACTGGAATGAGTGACTTGGATCCCTATCGATGGCCAAACTCAAAATGGAGGTGCTTCATGGTCAGATGGGATGAAGATATCGGGAGTGACCATCAAGAAAGAGTATCCCCATGGGAAATCGATCGGTCTGTTTCTCTCCCACCCTTGAACATTCAGTCCTCCCCAAGGCTGAAGAAACTGCGGACAAGTCTGCAGGCTACCCCACCTGATTACCCTGTCGCTGCAGGAGGCGGTGGGTTTTTGGACTTTGAGGATTCTATAAGAACCTCTAAGGTCTTGCAAGGTCAAGAAAATGTAGGTTTTGTATCACCCCTTTATGGACGTGATACGGCTAACCGCCCGCTGGATTTTGAGATGCGAGCCCCCATACATCAAAATCTTGCATTACCTGGATTAGAGAAAGGTAATGTTACTAAGTTTGTGGCTCACCCCACTACTTACACAGGCTTTATGGAATCCGATAGGTTTCCAAAGGTCTTGCAAGGTCAAGAAATATGCCCATTGAGATCCCTGGCCGGAAAAGTTGATCTCAATCTAGGTGCTTGGGGTAAACCCAATCTTGGTTGCAACTCTTTCAACATGTATCCAGCAGCCAAGCCTAGTTTCTACCCACTACCGTCAGAAAACCTTCGAACTATGTACTTTCCATATAATGACATGTACAAAAATGGTCAGGATCCCACGATGCGTGCTTATGCTACTAGTTTCCATAGAGAAAATGCCCAATTTAACTCACCTTCTATTCAGAAGAGGGTTGTTGGGGATGAAGTTAGAAAGCCTATTCTACTGAACGAGCATAAACCAGCCGACAGTATTCCTACTCCCACTTTTAAGACTACTATGAGAAACCAAAAAGAGGACACCTTTAGTGGAACAGTGGCTGGATGTAAACTTTTTGGATTCTCATTGACTGGGGAAACACCAAGTCCAAGCTCGCAAAACTCTGGTAAGAGAAGTTGTACGAAGGTTCACAAGCAAGGCAGCTTGGTTGGAAGAGCTATTGATCTCTCAAGACTGAATGGTTACAATGACTTGCTGACTGAACTGGAACAACTATTTAGCATGGAAGGCCTTCTACGAGATCCCGATAAAGGATGGCGGATCTTGTACACTGACAGCGAGAATGATGTGATGGTTGTTGGAGATGACCCTTGGCATGAATTCTGCAATGTGGTGTCCAAGATTCATATATACACCAAAGAAGAAGTGGAGAAGATGACCATTGGAATGATCACTGATGACACTCAAAGCTGTTTGGATCAAGCACCGGTGATAATGGAAGCTTCCAAGTCATCCTCAGTAGGCCAGCCGGATTCTTCTCCAACGGTAATAAGGATTTGA

>LcARF2A

ATGGCGAGGTCATTGAGAGACAGTGTGGTGGCGATCGACGGAGATGATGATTGGTCGGAGAGGAACGGCGGAGGAGCTGTAGGAGGGAAGGCTCTTTCCAGAGAACTGTGGCGTGCGTGTGCTGGTCCACTGGTGACGGTGCCGAGTAAAGGAGACGTTGTGTATTATTTTCCTCAGGGACATATGGAGCAGGTGGAGGCGTTGATGAATCAGGCAGCTGATCAGCAAATGCCGGCTTATGATATTTCGGAGAAGATAGTGTGTCGCGTCTTCAATGTTGTGTTGATGGCTGAACCGGACACTGATGAAGTTTTTTCTCAAGTGACTCTGCTTCCATTAACTAAATTCCAAGAGGAGGAGGCGGTTGCGGCGCAGAGGGAGAGAGGTCCACCGCCTCCGCGTCCTCGAGTTTATTCCTTCTGCAAGACTTTGACTGCTTCGGATACCAGCACTCACGGTGGATTCTCGGTGTTGAGACGCCATGCCGAAGAATGCCTGCCTCCTCTGGACATGTCCATGCAACCTCCAGCTCAGGATTTGGCGGCCAAGGACTTGCATGGAAATGAGTGGCAGTTTCGCCACATCTTTCGTGGTCAACCAAGGAGGCACCTTCTTCAAAGTGGCTGGAGTAATTTCGTCGGTTCCAAGAAGCTTGTTGCAGGAGATGCGTTTATTTTCCTCAGGGGTGAAAATGGGGAACTTCGGGTTGGGGTAAGGCGTGGTCTGATGCAGCCAAATACTGTTTCATCTTCAGTCATATCGAGCCATAGTATGCACATTGGTGTTCTTGCAACAGCCTGGCATGCAGTTGCAACGGGAACAATGTTCACCGTCTATTACAAACCAAGGACTAGCCCTGCCGACTTTATGGTTCCATATGACAAATATAACGAGGCTGCGAGGAACAACTATACTGTAGGAATGAGATTCAAAATGAGGTTTGAAGGTGAAGAGGGTCCAGAACAGAGACACTCAGGCACTGTTGTTGGCGTTGAAGATGTCGATCCAATTAAGTGGCCTGGTTCTATGTGGAGGTGCCTCAAGGTGCGGTGGGATGAAACTTCTTCTCGTAATCATCCAGAGAGAATTTCTCCATGGGAAGCAGAATTTTCTGTGACTCCTGATGCAGATCCCCTTCCAGTATATAGACTGAAAAGGCCTCGTCCAAATATGGTGTCATTGTCTAATGGTTCATCTGTTCACTCAACAGATGGTCCCACTGGAATGGCAGGTTCTAGGCACTCACAACAGTCCTGCAAATTCTCTGAGCGCTTTGTCAAGCAGAATCCTGAAGTTGCTGACCAAATGAAAAGGTGTGATGCAAACCAGGAAAGCATATACAAGTTGCTTCCAGAGTCCAGGATGCATGCGAATTCCCAATTTAAGATGGCAGAATCCAACATGGAGCTTCAAGCAAATGATAAAGAAATGCATTCTCAGAATCCTGCAAATATCCATTCTACTGTTGTCAGAGGGTATGATAGACCTCACGGTCTTGAATTCAGGAGGCAGGCTGAAAACTGGTTGTCGCCTTTGTCATCACCATCACATGCCAAAAAATCCTCCCATCCAGTGGAGTTGGATGCCCAGCCTCCAGCTGAGAAACAACAGGAGGTGGCGAAATCTAAAGCAGTTGCTAGTTGCAAACTCTTTGGTATCTCCCTTGTGAATAATTATGAGCCAACTGCACCGGCCATGCCAAATGCAAATTCTGTGTTTGGTCCACAACAGCGAATCAATTTTGCATCAGACCCTGTGCAAGATTTGGCTTCTGGCATACTTCCTGACCAATTGAAATGTACAAAGTCAGCTGAGACTGCACTTGGAGGCGATAAACTGGGCGAACCTTTTCAAGCTTCGGATCAGCTTTCTAGAGATGTCACGAGCAAGCTCCTGGTTGGTTCAACAAGAACTGGTGTTAAGGTTCACAAGCAAGGAAGCGCTGTTGGGAGGTCAGTGGATCTGACCAAGTTAAATGGCTATGATGAATTGGTCACAGAGCTAGATCGCATCTTTGAATTTAATGGTGAATTAACTGCTCCGAACAAAAAGTGGCTGATCGTGTTTACTGATGATGAAGGTGATATGATGCTAGTAGGAGACGACCCCTGGCTGGAATTCTGTAGCATGGTCCGTAGGATCTATGTTTACACTCGAGAGGAGGTGAATCAGATGATACCCCGTCCCCTGAATCCAAAAATCAAGAAAACTTTGTTCATGGCAGATAAAAAGACCGGATCAACAGAGGCTAAGAGTTTGGCTCATGCTTAA

>LcARF2B

ATGGCGAGGTCATTGAGAGACAGTGTGGTGGCGATCGACGGAGATGATGATTGGTCGGAGAGGAACGGCGGAGGAGCTGTAGGAGGGAAGGCTCTTTCCAGAGAACTGTGGCGTGCGTGTGCTGGTCCACTGGTGACGGTGCCGAGTAAAGGAGACGTTGTGTATTATTTTCCTCAGGGACATATGGAGCAGGTGGAGGCGTTGATGAATCAGGCAGCTGATCAGCAAATGCCGGCTTATGATATTTCGGAGAAGATAGTGTGTCGCGTCTTCAATGTTGTGTTGATGGCTGAACCGGACACTGATGAAGTTTTTTCTCAAGTGACTCTGCTTCCATTAACTAAATTCCAAGAGGAGGAGGCGGTTGCGGCGCAGAGGGAGAGAGGTCCACCGCCTCCGCGTCCTCGAGTTTATTCCTTCTGCAAGACTTTGACTGCTTCGGATACCAGCACTCACGGTGGATTCTCGGTGTTGAGACGCCATGCCGAAGAATGCCTGCCTCCTCTGGACATGTCCATGCAACCTCCAGCTCAGGATTTGGTGGGCAAGGACTTGCATGGAAATGAGTGGCAGTTTCGCCACATCTTTCGCGGTCAACCAAGGAGGCACCTTCTTCAAAGTGGCTGGAGTAATTTCGTCAGTTCCAAGAAGCTTGTTGCAGGAGATGCGTTTATTTTCCTCAGGGGTGAAAATGGGGAACTTCGGGTTGGGGTAAGGCGTGGTCTGATGCAGCCAAATACTGTTTCATCTTCAGTCATATCGAGCCATAGTATGCACATTGGTGTTCTTGCAACAGCCTGGCATGCAGTTGCAACGGGAACAATGTTCACCGTCTATTACAAACCAAGGACTAGCCCTGCCGACTTTATGGTTCCATATGACAAATATAACGAGGCTGCGAGGAACAACTATACTGTAGGAATGAGATTCAAAATGAGGTTTGAAGGTGAAGAGGGTCCAGAACAGAGACACTCAGGCACTGTTGTTGGCGTTGAAGATGTCGATCCAATTAAGTGGCCTGGTTCTATGTGGAGGTGCCTCAAGGTGCGGTGGGATGAAACTTCTTCTCGTAATCATCCAGAGAGAATTTCTCCATGGGAAGCAGAATTTTCTGTGACTCCTGATGCAGATCCCCTTCCAGTATATAGACTGAAAAGGCCTCGTCCAAATATGGTGTCATTGTCTAATGGTTCATCTGTTCACTCAACAGATCGTCCCACTGGAATGGCAGGTTCTAGGCACTCACAACAGTCCTGCAAATTCTCTGAGCGCTTTGTCAAGCAGAATCCTGAAGTTGCTGACCAAATGAAAAGGTGTGATGCAAACCAGGAAAGCATATACAAGTTGCTTCCAGAGTCCAGGATGCATGCGAATTCCCAATTTAAGATGGCAGAATCCAACATGGAGCTTCAAGCAAATGATAAAGAAATGCATTCTCAGAATCCTGCAAATATCCATTCTACTGTTGTCAGAGGGTATGATAGACCTCACGGTCTTGAATTCAGGAGGCAGGCTGAAAACTGGTTGTCGCCTTTGTCATCACCATCACATGCCAAAAAATCCTCCCATCCAGTGGAGTTGGATGCCCAGCCTCCAGCTGAGAAACAACAGGAGGTGGCAAAATCTAAAGCAGTTGCTAGTTGCAAACTCTTTGGTATCTCCCTTGTGAATAATTATGAGCCAACTGCACCGGCCATGCCAAATGCAAATTCTGTGTTTGGTCCACAACAGCGAATCAATTTTGCATCAGACCCTGTGCAAGATTTGGCTTCTGGCATACTTCCTGACCAATTGAAATGTACAAAGTCAGCTGAGACTGCACTTGGAGGTGATAAACTGGGCGAACCTTTTCAAGCTTCGGATCAGCTTTCTAGAGATGTCACGAGCAAGCTCCTGGTTGGTTCAACAAGAACTGGTGTTAAGGTTCACAAGCAAGGAAGCGCTGTTGGGAGGTCAGTGGATCTGACCAAGTTAAATGGCTATGATGAATTGGTCACAGAGCTGGATCGCATCTTTGAATTTAATGGTGAATTAACTGCTCCGAACAAAAAGTGGCTGATCGTGTTTACTGATGATGAAGGTGATATGATGCTAGTAGGAGACGACCCCTGGCTGGAATTCTGTAGCATGGTCCGTAGGATCTATGTTTACACTCGAGAGGAGGTGAATCAGATGATACCCCGTCCCCTGAATCCAAAAATCAAGAAAACTTTGTTCATGGCAGATAAAAAGACCGGATCAACAGAGGCTAAGAGTTTGGCTCATGCTTAA

>LcARF2C

ATGGCCGCTGCTACTTCTTCAGAGGTATCAATAAAGTCTTCTAACGAGACTAGTAGGAGTCCTTTGGAAGGGCAGAAAGGTAATTCCACAAATTCCGGAGCCAGAAGAGTTATAGACCCTGAAATGGCCCTCTACACGGAGCTTTGGCACGCGTGTGCTGGTCCTTTAGTCACCGTCCCTCGTGAAGGCGAGCGCGTGTACTACTTTCCTCAAGGTCACATAGAGCAGGTGGAGGCGTCAACTAATCAGGTCGCTGACCAGCAAATGCCAGTTTACGATCTTCCATCGAAGATACTCTGTCGTGTGATTAACGTCCAATTAAAGGCTGAACCTGATACTGATGAGGTTTTCGCTCAAGTGACTTTGCTTCCTGATTCAAACGACGAAAAGGTGGTGGAGAAGGAACCTCCTCCGCAGCCTCCGCCTAGATTTCATGTGCATTCGTTCTGTAAGACGTTGACTGCCTCGGATACTAGTACTCATGGAGGGTTTTCGGTGCTAAGGCGGCATGCTGATGAATGTCTTCCTCCATTGGACATGTCGCGACAGCCTCCGACACAGGAGTTGGTTGCCAAGGATTTGCATGGAAATGAGTGGCGGTTCCGGCATATTTTTAGAGGTCAACCTCGTAGACACCTGCTACAAAGCGGATGGAGTGTTTTTGTCAGCTCAAAGAGGCTGGTGGCAGGGGATGCTTTTATATTCCTGAGGGGTGAGAATGGGGAACTTCGTGTAGGCGTAAGACGTGCTATGAGGCAGCAGAATAATGTTCCGTCCTCAGTCATATCCAGTCATAGTATGCATCTTGGTGTTCTTGCCACCGCATGGCATGCGATCTCAACAGGAACCATGTTCACTGTCTATTACAAACCTAGGACAAGCCCTGCGGAGTTTATTGTTCCATTCGATCAATACATGGAATCCGTCAAGAATAATTATTCTATTGGAATGAGATTCAAAATGAGATTTGAAGGCGAAGAGGCCCCTGAGCAGAGGTTTACTGGCACCATTGTTGGGATTGAAGAGGCTGATCCGCAAAGGTGGCGAGATTCCAAATGGAGATGCCTCAAGGTGAGATGGGATGAAACTTCCACCATACCTCGTCCAGAAAGAGTTTCACCTTGGAAGATAGAACCTGCACTGGCTCCTCCAGCACTCAATCCTCTTCCAATGCCTCGGCCTAAAAGGCCAAGAACAAACATGTTACCTTCATCCCCTGACTCCTCTGTTCTTACCAGGGAAGGTTCGTCAAAAGTAAATGTAGACCCTTCATCAGCTAGTGGTTTTTCAAGGGTCTTGCAAGGTCAAGAAACCTTGAGAGGCAATTTTGCTGAGAGAGAGAGTAATGAGTCTGAGAATGCTGAGAAGTCTGTGGCATGGCCACCTCAACTTGAGGATGAGAAGATTGATGTGGTTTCTGGCTCAAGAAGATATGGACCTGAGAGCTGGGTGCCATCGGGGAGGCATGAACCAACTTACACAGATTTGCTCTCAGGCTTTGGGCCAAATGCTGATTCCTCCCATGGGTATGGCGCTTCCTTTGTCGATCAGTCTCTACCATCTGCCTGTTCAGCAAGAAAAAGCTTGTTGGATCAAGATGGAAAATTTAGCTTGCCAGCCCGTCCCTGGTCCTTGATGCCCACTCCTCTTTCGCTTAAGTTAGCAGAGTCTAATGCAAAGATTCCTGGACAAGGTGGTGATGTTACTTATCAAGTACGAGGTAATGTTAGATATGGTGGGTTTGGTGACTATCCCATGCTTCATCCTCATAGAATCGAGCAGTCACATGGAAATTGGTTGATGCCCCCACCCCCTCAATCTCAGTTTGAAAATCCAACTCATTCAAGAGAGTTAATGCCGAAACCGGTGTTGGTACAAGACCGTGAGGCTGGAAAATCGAAAGATGGGAATTGCAAGCTCTTTGGTATTCCCCTTTTCAGTAACCCTGTTGTGCCTGAGCTAACTGTATCGCAGAGAAACACAATGAATGATCCTGGCGGTCATGCACATCACCAATACTGTGCATTTGAATCTGATCAAAAGTCAGAACAAACAAAGGGCTCAAAATTGGCAGATGATGGTCCTGCTGTTAATGAACAGGAGAAACCATCCCAGCCCTGTCAGTCCCACACGAAGGACACTCGTAATAAACCTCACAGTGTGTCATCAAGGAGTTGTACTAAGGTCCACAAGCAGGGAATTGCGCTTGGTAGGTCTGTGGACCTTTCTAAGTTCAACAACTACGAGGAATTGATTGCTGAGTTAGATCGGTTGTTTGAATTTGCTGGTGAATTAATGGCCTCCAAGAACTGGTTAATTGTATATACTGATGATGAAGATGACATGATGCTTGTCGGAGATGATCCTTGGCAGGAGGAGGTCCAGAAGATGAACCCAGGGTCCTTAAGTTCAAAGTGTGAGGAGAATCCATTGAGTGCAGAAGGTTTGGATGCAAAAGAAGTGAAGCATCTGCCACTCCCTTCCGCATTGAATGCAGAGCATTGTTAG

>LcARF2D

ATGGCCGCTGCTACTTCTTCAGAGGTATCAATAAAGTCTTCTAACGAGACTAGTAGGAGTCCTTTGGAAGGGCAGAAAGGTAATTCCACAAATTCCGGAGCCAGAAGAGTTATAGACCCTGAAATGGCCCTCTACACGGAGCTTTGGCACGCGTGTGCTGGTCCTCTAGTCACCGTCCCTCGTGAAGGCGAGCGCGTGTACTACTTTCCTCAAGGTCACATAGAGCAGGTGGAGGCGTCAACTAATCAGGTCGCTGACCAGCAAATGCCAGTTTACGATCTTCCATCGAAGATACTCTGTCGTGTGATTAACGTCCAATTAAAGGCTGAACCTGATACTGATGAGGTTTTCGCTCAAGTGACTTTGCTTCCTGATTCAAACGACGAAAAGGTGGTGGAGAAGGAACCTCCTCCGCAGCCTCCGCCTAGATTTCATGTGCATTCGTTCTGTAAGACGTTGACTGCCTCGGATACTAGTACTCATGGAGGGTTTTCGGTGCTAAGGCGGCATGCTGATGAATGTCTTCCTCCATTGGACATGTCGCGACAGCCTCCGACACAGGAGTTGGTTGCCAAGGATTTGCATGGAAATGAGTGGCGGTTCCGGCATATTTTTAGAGGTCAACCTCGTAGACACCTGCTACAAAGCGGATGGAGTGTTTTTGTCAGCTCAAAGAGGCTGGTGGCGGGGGATGCTTTTATATTCCTGAGGGGTGAGAATGGGGAACTTCGTGTAGGCGTAAGACGTGCGATGAGGCAACAGAATAATGTTCCGTCCTCAGTCATATCCAGTCATAGTATGCATCTTGGTGTCCTCGCCACTGCATGGCATGCGATCTCAACAGGAACCATGTTCACTGTCTATTACAAACCTAGGACAAGCCCTGCGGAGTTTATTGTTCCATTCGATCAATACATGGAATCCGTCAAGAATAATTATTCTATTGGAATGAGATTCAAAATGAGATTTGAAGGCGAAGAGGCCCCTGAGCAGAGGTTTACTGGCACCATTGTTGGGATTGAAGAGGCTGATCCGCAAAGGTGGCGAGATTCCAAATGGAGATGCCTCAAGGTGAGATGGGATGAAACTTCCACCATACCTCGTCCAGAAAGAGTTTCACCTTGGAAGATAGAACCTGCACTGGCTCCTCCAGCACTCAATCCTCTTCCAATGCCTCGGCCTAAAAGGCCAAGAACAAACATGTTGCCTTCATCCCCTGACTCCTCTGTTCTTACCAGGGAAGGTTCGTCAAAAGTAAATGTAGACCCTTCATCAGCTAGTGGTTTTTCAAGGGTCTTGCAAGGTCAAGAAACCTTGAGAGGCAATTTTGCTGAGAGAGAGAGTAATGAGTCTGAGAATGCTGAGAAGTCTGTGGCATGGCCACCTCAACTTGAGGATGAGAAGATTGATGTGGTTTCTGGCTCAAGAAGATATGGACCTGAGAGCTGGGTGCCATCGGGGAGGCATGAACCAACTTACACAGATTTGCTCTCAGGCTTTGGGGCAAATGCTGATTCCTCCCATGGGTATGGTGCTTCCTTCGTCGATCAGTCTTTACCGTATGCCAGTTCAGCAAGAAAAAGCTTGTTGGATCAAGATGGAAAGTTTAGCTTGCCGGCCCGTCCCTGGTCCTTGATGCCCACTCCTCTTTCGCTTAAGTTAGCAGAGTCTAATGCAAAGATTCCAGGACAAGGTGGTGATGTTACTTATCAAGTACGAGGTAATGTTAGGTATGGTGGGTTTGGTGACTATCCCATGCTTCATCCTCATAGAATCGAGCAGTCACATGGAAATTGGTTGATGCCCCCACCCCCTCAATCTCAGTTTGAAAATCCAACTCATTCAAGGGAGTTAATGCCGAAACCTGTATTGGTACAAGACCGTGAGGCTGGAAAATCGAAAGATGGGAATTGCAAGCTCTTTGGTATTCCCCTTTTCAGTAACCCTGTTGTGCCTGAGCTAACTGTATCGCAGAGAAACACAATGAATGATCCTGGCGGTCATGCACATCACCAATACTGTGCATTTGAATCTGATCAAAAGTCAGAACAAACAAAGGGCTCAAAATTGGCAGATGATGGTTCTGCTGTTAATGAACAGGAGAAACCATCCCAGCCCTGTCAGCCCCAGACGAAGGACACTCGTAATAAAACTCACAGTGTGTCAGCAAGGAGTTGTACTAAGGTCCACAAGCAGGGAATTGCGCTTGGTAGGTCTGTGGACCTTTCTAAGTTCAACAACTACGAGGAATTGATTGCCGAGTTAGATCGGTTGTTTGAATTTGGTGGTGAATTAATGGCTTCCAAGAACTGGTTAATTGTATATACTGACGATGAAGATGATATGATGCTTGTCGGAGATGATCCCTGGCAGTATGGATTCTTTTCTCAAGCATTGATAATATATTACATCTGA

>LcARF18A

ATGGCGCATGTGGAGGGTAATCCGAGAAGCTCTTCGATTTCTCACGTTGGAACAGGTACAAGCTGTGACGATCTATACAGGGAGCTATGGAAGGCATGTGCAGGGCCATTGGTGGAGGTTCCTCGATTTGGAGAGAGAGTTTTCTATTTCCCTCAGGGTCACGTGGAACAATTGGAAGCATCTACCAATCAGGAACTGCAACATCAAAAACCTCTGTTTAGTCTTGCTTCTAAGATACTTTGTCGTGTTGTCAACATTATGTTACTGGCTGAACCAGAGTCAGATGAGGTTTATGCCCAGATCACTTTGCACCCAGAACAAGAGCAAAGTGAACCTACAAATCCAGACCAAAGCCCACCTGAATCTCCAAAGCAAACGTTACATTCATTTTGTAAGATTTTAACAGCTTCCGATACAAGTACTCACGGCGGATTCTCCGTTCTTCGCAAGCATGCCACTGAATGCCTGCCTCCATTGGACATGAACCAAGCAACCCCAACTCAAGAACTGATTGCCAAAGACCTTCATGGGTATGAATGGCGGTTCAAACATATATTTAGAGGCCAACCACGGAGGCATTTGCTGACAACTGGGTGGAGTACATTTGTTACTTCTAAAAGATTGGTTGCAGGAGATGCTTTTGTGTATTTGAGGGGTGAGAATGGGGAATTAAGAATTGGGGTTCGGCGACTTGCTCGTCAGCAGAGCCCGATGCCTTCATCTGTGATATCCAGCCAGAGCATGCATTTAGGAGTCCTTGCGACTGCTTCCCATGCTGTTATGACTAACACTCTCTTTGTTGTATACTACAAGCCAAGGACAAGCCAGTTTATCATTGGTGTAAACAAATACCTGGAATCTGTGAAATATGGGTTTTCTGTTGGTATGCGTTTCAAAATGAGATTTGAAGGAGAAGACTCACCTGAAAGAAGGTTCACGGGTACCATAGTTGGGGGTGGAGATAGTTCTCCACAGTGGCCAGGTTCTGAGTGGCGGTCATTGAAGATACAATGGGATGAACCGGCTACAATTTCAAGGCCAGAGAGGGTTTCTCCATGGGAGATAGAGCCTTTTGTAACTTCTGCACCACCTTTAAATCTTGCTCAACCAGCAGTTAAGAACAAGAGGCCCCGGTCTGTTGACATTCAAGCTTCTGAAATTACGACAAATTCGGCAACTTCAGCTTTCTGGTATCAGGGATCAAACCATTCCCATGATTTGACACAACTGGGGAGTGCTGCTGAAGTCCAAAGCGGTGAAAGACAAGTCTGGCCAATGAGGCAGAAAGAAACTGATTGCAATCTTGTCAGCAGCACTAATGGCTGTAACTCTAGGTTTCCTCCAGAAGGCATGTGGCCTTCTTCTCCTCATTTCAATGTCTCTCTGAACCTTTTCCCAGATTCATCAGAGGATGGCAAGATTGCTGTGCCTAGGTCTGTTCTCTCTAGTTATGCCTCTTCGGTTCCTTCAAGGCTAAGCAATGACCTGACTCACGATCAGGTGGAGAAAAGGAAACAATCTGAGACTTCTACAAGTTGCCGTTTGTTTGGAATTGTCCTGAAAAACAACTCTAATGCTGCTGCTCCTTTAGGGAAGGAAGTTATAAGTTCAACTATGGATTCCAGTGGCACTAAAGGATCCGTTTCAGCTGCCTGCACTGCTGATAAGGATCCAAATTTTGATAGTTCAAAGTCCTTTATGGAGCTGAATCAAGTGACAATGGAAATGCCACTGAAAGAGTTGCAGAACAAGCAGGCTTCAAGTACTTCCATGAGAACTCGTACAAAGGTTCAAATGCAAGGAATTGCTGTTGGTCGTGCTGTTGACTTAACTGTATTGAAGGGGTACAATGATCTTATCGATGAACTAGAAAAGATGTTTGAGATCCAAGGACAGCTGCGCCCACATGATAAATGGGCAGTCGTTTTTACTGATGATGAGGGTGATATGATGCTTGTGGGGGATGATCCATGGCCGGAATTCTGTAAGATGGTGAAAAAGATCTACATATACTCTAATGAAGAAGTGAAGAAGCTGACCGCAAGAAGCAAGAATGCTGCATCATCTATGGAGGGTGAAGGAACAGTCATAAGCTTGGATTCAGAGCATAGGTCCGAAGCGTGA

>LcARF18B

ATGGCGCATGTGGAGGGTAATCCGAGGAGCTCTTCGATTTCTCACGTTGGAACAGGTACAAGCTGTGACGATCTATACAGGGAGCTATGGAAGGCATGTGCAGGGCCATTGGTGGAGGTTCCTCGATTTGGAGAGAGAGTTTTCTATTTCCCTCAGGGTCACGTGGAACAATTGGAAGCATCTACTAATCAGGAACTGCAACATCAAAAACCTCTGTTTAGTCTTGCTTCTAAGATGCTTTGTCGTGTTGTCAACATTATGTTACTGCAAAGTGAACCTACAAATCCAGACCAAAGCCCACCTGAGTCTCCAAAGCAAACGGTACATTCATTTTGTAAGATTTTAACAGCTTCCGATACAAGTACTCACGGCGGATTCTCCGTTCTTCGCAAGCATGCCACTGAATGCCTGCCTCCATTGGACATGAACCAAGCAACTCCAACTCAAGAACTGATTGCCAAAGACCTTCATGGGGGTGAGAATGGGGAATTAAGAGTTGGGGTTCGGCGACTTGCTCGTCAGCAGAGCCCGATGCCTTCATCTGTGATATCCAGCCAGAGCATGCATTTAGGAGTCCTTGCGACTGCTTCCCATGCTGTTATGACTAACACTCTCTTTGTTGTATACTACAAGCCAAGGACAAGCCAGTTTATCATTGGTGTAAACAAATACCTGGAATCTGTGAAATATGGGTTTTCTGTTGGTATGCGTTTCAAAATGAGATTTGAAGGAGAAGACTCACCTGAAAGAAGGTTCACGGGTACCATAGTTGGGGTTGGAGATATTTCTCCACAGTGGCCAGGTTCTGAGTGGCGGTCGTTGAAGATACAATGGGATGAACCGGCTACAATTCCAAGGCCAGAGAGGGTTTCTCCATGGGAGATAGAACCTTTTGTAACTTCTGCACCACCTTTAAATCTTGCTCAGCCAGCAGTTAAGAACAAGAGGCCCCGGTCTGTTGACATTCAAGCTTCTGCTTTCTGGTATCAGGGATCAAACCATTCCCATGATTTGACACAACTGGGGAGTGCTGCTGAAGTCCAAAGCGGTGAAAGACAAGTCTGGCCGATGATGCAGAAAGAAACTGATTGCAATCTTGTCAGCAGCACTAATGGCTGTAACTCTAGGTTTCCTCCAGAAGGCATGTGGCCTTCTTCTCCTCATTTCAATGTCTCTCTGAACCTTTTCCCAGATTCATCAGAGGATGGCAAGATTGCTGTGCCTAGGTCTGTTCTCTCTAGTTACGCCTCTTCAGTTCCTTCAAGGCTAAGCAATGACCTGACTCACGATCAGGTGGAGAAAAGGAAACAATCTGAGACTTCTAGGAGTTGCCGTTTGTTTGGAATTGTCCTGAAAAACAACTCTAATGCTGCTGCTCCTTTAGGGAAGGAAGTTACAAGTTCAACTATGGATTCCAGTGGCACTAAAGGATCCGTTCCAGCTGCCTGCACTGCTGATAAGGATCCAAATTTTGATAGTTCAAAGTCCTTTATGGAGCTGAATCAAGTGACACTGGAAATGCCACTGAAAGAGTTGCAGAACAAGCAGGCTTCAACTACTTCCATGAGAACTCGTACAAAGGTTCAAATGCAAGGAATTGCTGTTGGTCGTGCTGTTGACTTAACTGTATTGAAGGGGTACAATGATCTTATCGATGAACTAGAAAAGATGTTTGAGATCCAAGGACAGCTGCGCCCACATGATAAATGGGCAGTGGTTTTTACTGATGATGAGGGTGATATGATGCTTGTGGGGGATGATCCATGGCCGGAGTTCTGTAAGATGGTGAAAAAGATCTACATATACTCTAACGAAGAAGTGAAGAAGCTGACCGCAAGAAGCAAGAATGCTGCATCATCTATGGAGGGTGAAGGAACAGTCATAAGCTTGGATTCAGAACATAGGTCCGAAACGTGA

>LcARF9A

ATGACGGGGCGGGTCGGGTCGTTTTCGCAGCCAAGTAGTAACAGTTCAGATGATCTGTACGAGGAGCTATGGAAGGCTTGTGCGGGGCCACTGGTGGATGTGCCGAAGTCTGGAGAGAAAGTGTTTTATTTTCCGCAAGGTCACATGGAGCAATTGGAGGCTTCGACTAATCAGGAACTGAATCAGAGGATTCCCTTGTTTCAGCTACGCCCCAAGATTCTCTGTCGGGTTATGAACATTCAGCTGATGGCGGAACAAGAAACAGATGAGGTTTATGCTCAAATTACTTTGCTGCCAGAGGCGAATCAAGATGAGCCAACGTTGCCTGATCCATGTCCTCCTGAATCGGCAAGACCAACTGTTCACTCGTTTTGTAAGGTTTTAACGGCATCTGATACAAGCACCCATGGAGGGTTTTCTGTTTTGCGGAAACATGCTACCGAATGTCTTCCGCCGCTGGACATGAATCAGTCGACCCCAACTCAGGAATTGGTTGCCAAAGATTTGCACGGTTATGAGTGGCGATTTAAGCATATTTTCAGAGGCCAACCAAGGAGACACCTGCTTACAACCGGATGGAGTACGTTTGTTACTTCTAAGAGATTAGTTGCTGGGGATACCTTTGTATTCTTGAGGGGGGAGAATGGGGACTTGCGTGTTGGAGTCAGGCGTCTTGGTCATCAACAGAGTAGCATGCCATCTTCTGTGATTTCAAGTCAGAGCATGCACCTCGGAGTGCTTGCAACAGCATCTCATGCTGTCGCAACCCGAACACGATTTGTGGTCTATTATAAGCCACGGACAAGTCAGTTCATCGTCGGTGTAAACAAATATTTAGAGGCTATTAACAACAAGTTTGCAGTTGGGATGAGATTCAAGATGAGGTTTGAGGGAGAGGAATCTCCTGAGAGAAGGTTCTCTGGCACAATAGTTGGGGTGGAAGACTTTTCTCCTCACTGGAATGATTCAAAATGGCGATCACTGAAAGTTCACTGGGACGAACCTGCCTCTATTTCAAGGCCTGATAGGGTTTCATCATGGGAGATCGAACCTTTTGTGGCATCAGTACCATCTAATGTGGCTCAACCAGTTTCAGCTAAGAACAAAAGGCCTCGACCACCTATTGAAATTCCAGCTCTTGATATGTCGCCAACAGCATCAGCTTCTTGGAATTCGAGATTTACACAGTCCCAGGATCTGACACAACTGAGTGTTACTGCTGAAGGGAAAAGAAGTGATAATCATATTGCATGGCATCACAAACAGACTGATTTTAACAACAATAGCAACTCTATGTCAAGGACTGATGGGGACTGGCTAACTTCTCCTCATGTCAGTTTTTCACAGCATCTATTTCAGGATACAATGGACGACAATAAAAGTGTCTCTGCGTGGCCTGCTGTTTCTGGATATTTGACTCCGCAGTCATCAAAGGTGAACAATGAATCGATGCTCGACGGACCTGAAACAGGGAGGAAAACTGAGATGGCTACTAGTTGCCGATTGTTTGGTTTTGATCTCATTAATCATTCTATGGCCTCTAATACGACAGAGAAAGCGCCAGTTTCTAGTATCACGACAGAAGGGCACATTCTCGGTACCCCTCCAGCAGCTGATTCTGACCAGAAGTCTGACCTTTCAAAGGCTTTCAAAGAAATGAAAGAACAACTGCAGGTATCGCCTAAAGAGACACAGAGTCGGCAAAGTTGTTCTACATCTACCAGAAGTCGCACCAAGGTTCAAATGCAGGGAGTTGCTGTTGGTCGAGCAGTGGACCTGACTATGTTGGAAGGGTATGATCAGCTTATTGATGAACTGGAGGAGATGTTTGACATTAAGGGACAGCTTCACAGTAGGAATAAGTGGGAAATTGTCTACACTGATGATGAAGGAGATATGATGCTTGTAGGGGACGATCCATGGGTTGAATTCTGTAACATGGTGAAGCGAATTTTCATATGTTCTAGCCAAGATGTGAAGAAGATGACTCCAGGAAGCAAACTTCCTATGTCCTCCATGGAAGGTGAAGGGATTTTACTAAGCTCAGACTCAGCTGAAAACTGA

>LcARF9B

ATGATGACGGGGCGGGTCGGGTCGTTTTCGCAGCCAAGTAGTAACAGTTCAGATGATCTGTACGAGGAGCTATGGAAGGCTTGTGCGGGGCCACTGGTGGATGTGCCGAAGTCTGGAGAGAAAGTGTTTTATTTTCCGCAAGGTCACATGGAGCAATTGGAGGCTTCGACTAATCAGGAACTGAATCAGAGGATTCCCTTGTTTCATCTACGCCCCAAGATTCTCTGTCGGGTTATGAACATTCAGCTGATGGCGGAACAAGAAACAGATGAGGTTTATGCTCAAATTACTTTGCTGCCAGAGGCGAATCAAGATGAGCCAACGTTGCCTGATCCATGTCCTCCTGAATCGGCAAGACCAACTGTTCACTCGTTTTGTAAGGTTTTAACGGCATCTGATACGAGCACCCATGGAGGGTTTTCTGTTTTGCGGAAACATGCTACTGAATGTCTTCCGCCGCTGGACATGAATCAGTCGACCCCAACTCAGGAATTGGTTGCCAAAGATTTGCACGGTTATGAGTGGCGATTTAAGCATATTTTCAGAGGCCAACCAAGGAGACACCTGCTTACAACCGGATGGAGTACGTTCGTTACTTCTAAGAGATTAGTTGCTGGGGATACCTTTGTATTCTTGAGGGGGGAGAATGGGGACTTGCGTGTTGGAGTCAGGCGTCTTGGTCATCAACAGAGTAGCATGCCATCTTCTGTGATTTCAAGTCAGAGCATGCACCTCGGAGTGCTTGCAACAGCATCTCATGCTGTCGCAACCCGAACACGATTTGTGGTCTATTATAAGCCACGGACAAGTCAGTTCATCATCGGTGTAAACAAATATTTAGAGGCTATTAACAACAAGTTTGCAGTTGGGATGAGATTCAAGATGAGGTTTGAGGGAGAGGAATCTCCTGAGAGAAGGTTCTCTGGCACAATAGTTGGGGTGGAAGACTTTTCTCCTCACTGGAATGATTCAAAATGGCGGTCACTGAAAGTTCACTGGGACGAACCTGCCTCTATTCCAAGGCCTGATAGGGTTTCATCATGGGAGATTGAACCTTTTGTGGCATCAGTACCATCTAATGTGGCTCAACCAGTTTCAGCTAAGAACAAAAGGCCTCGACCGCCTATTGAAATTCCAGCTCTTGATATGTCGCCAACAGCATCAGCTTCTTGGAATTCGAGATTTACACAGTCCCAGGATCTGACACAACTGAGTGTTACTGCTGAAGGGAAAAGAAGTGATAATCATATTGCATGGCATCACAAACAGACTGATTTTAACAACAATAGCAACTCTATGTCAAGGACTGATGGGGACTGGCTAACTTCTCCTCATGTCAGTTTTTCACAGCATCTATTTCAGGATACAATGGACGACAATAAAAGTGTCTCTGCGTGGCCTGCTGTCTCTGGATATTCGACTCCGCAGTCATCAAAGGTGAACAATGAATCAATGCTCGACGGACCTGAAACAGGGAGGAAAACTGAGATGGCTACTAGTTGCCGATTGTTTGGTTTTGATCTCATTAATCATTCTATGGCCTCTACTACGACAGAGAAAGCACCAGTTTCTAGTATCACGACAGAAGGACACATTCTCGGTACCCCTCCAGCAGCTGATTCTGACCAGAAGTCTGACCTTTCAAAGGCTTTCAAAGAAATGAAAGAACAACTGCAGGTATCGCCTAAAGAGACACAGAGTCGGCAAAGTTGTTCTACATCTACCAGAAGTCGCACCAAGGTTCAAATGCAGGGAGTTGCTGTTGGTCGAGCAGTGGACCTGACCATGTTGGAAGGGTATGATCAGCTTATTGATGAACTGGAGGAGATGTTTGACATTAAGGGACAGCTTCGCAGTAGGAATAAGTGGGAAATTGTCTACACTGATGATGAAGGAGATATGATGCTTGTAGGGGACGATCCATGGGTTGAATTCTGTAACATGGTGAAGCGAATTTTCATATGTTCTAGCCAAGATGTGAAGAAGATGACTCCAGGAAGCAAACTTCCTATGTCCTCCATGGAAGGTGAAGGGATTTTACTAAGCTCAGACTCAGCTGAAAACTGA

>LcARF1B

ATGCATCAGGGGCTGGAGCAGCCAATGCATTCATTAAATCTGCCATCTAAAATTCTTTGTAGAGTGATTAATGTTCAGCATCGGGCCGAACCTGAAACAGATGAAGTTTATGCTCAAATAACTTTGTTCGCTGAATCTGATCAAAGTGAGGTTACAAGACCTGATCCTCCTCTGCCTGAACCTGAAGAGTACACCGTCCAATCATTTTGCAGAACACTTGTTGCTTCTGATGTAAGCATCCATGGTGGATTGTCTGTTCCCAAGAAGCATGCTATTGAGTGCCTGCCTCCGCTGGATATCACCCAGCAACCTCCATCTCATAAATTGGTTGCAACTGATCTTCATGGAAATGAATGGGAACAAAATGGGGAGCTGCGTGTTGGAGTTAGGAGACACAAAAGAGAGGAAGTGAATAGGCCATCTTTGGTTATATCTATCGGCAACATCAATCTTGATGAACTTGCAACTGCTTCCCAAGCCATTGCATCTGGAATGATGTTTTCTATCGTCTACAAGCCAAGAATTAGCCGGTCAGGGTTTATTGTCAGCGTCGACAAGTACCTTGAAGCAATGAGATACAATTTCTCTGTTGGGATGAGATTCAAGATGAGATATGAGGGTGGGGAGGTTCTTGAGCAAAGTGGCACAGTTGTTGGCATTGAAGATTATGATCGCTCGACTTGGCCCAACTCTGAGTGGAGGTGCTTAAAGGTTCAATGGGATGCACATTTGTCAATCCCATGCCCGACTAGAGTCTCACAATGGGAATTGGAGCCATTTTGA

>LcARF1C

ATGAATCTCTACAATGTGCGGCTGCCGGCGCCGGTAGTGGAGGACGCCAAGCTAGAGAAGGACTCGTTCGCCTCCGTCGTCTCCTCCATCAAGCTTGCCTTCCGCCACGACGATCCTGAATCCTTCTTCTCCAGTCTCAAATGGATCTCCGTCCTCGACCTGGTTTCGTCAACTCCACAACTTCAAGATTCTGCGATTTGTTCTGGATTTGGACTGAGAGACTCTGCAAGTGCTGGTAGCTGGAAGAAAGTGCCAGAAACGGCTCATAGAATCTTGCTGCAGGAGATGAATTTCATATTGCCTATTGCATTCGAAAAATCTTTATTCAGCATTCATGTATCTGCTATTGCTGCTCCACCAGCATTCAGAAGTTCAGTGAGAAGTTCCCTGGGTCAACTGACTAGTTATCTTTCCAGGATTGGTGGAGGTCTCGTTGTGGCATTCCCTGCAGCAGCTGCAGTGCCTTTGAATCATTCCTCTGGAGGACCCCTTTCAGGGGTACTCTCCAATGATGCTTTGTACAGAGAACTATGGCATGCTTGTGCTGGACCCCTTGTCACCCTCCCTCGTGAAGGCGAGTGTGTTAATTACTTTCCACAGGGGCATACAGAACAGCTAGAAGCATCAATGCATCAGGGGCTGGAGCAGCCAATGCATTCATTAAATCTGCCATCTAAAATTCTTTGTAGAGTGATTAATGTTCAGCATCGGGCCGAACCTGAAACAGATGAAGTTTATGCTCAAATAACTTTGTTCGCTGAATCTGATCAAAGTGAGGTTACAAGACCTGATCCTCCTCTGCCTGAACCTGAAGAGTACACCGTCCAATCATTTTGCAGAACACTTGTTGCTTCTGATGTAAGCATCCATGGTGGATTGTCTGTTCCCAAGAAGCATGCTATTGAGTGCCTGCCTCCGCTGGATATGACCCAGCAACCTCCATCTCATAAATTGGTTGCAACTGATCTTCATGGAAATGAATGGTGTTTTCGGCACATTCTACGAGGTTACATAATTCAGGTCAACCTAGACGTCATTTGCTTACAACTGGATGGAGTGCATTCATCACTTCCAAAAAATTGGGAACAAAATGGGGAGCTGCGTGTTGGAGTTAGGAGACACAAAAGAGAGGAAGTGAATAGGCCATCTTTGGTTATATCTATTGGCAACATCAATCTTGATGAACTTGCAACTGCTTCCCAAGCCATTGCATCTGGAATGATGTTTTCTATCGTCTACAAACCAAGAATTAGCCGGTCAGGGTTTATTGTAAGCGTCAACAAGTACCTTGAAGCGATGAGATACAATTTCTCTGTTGGGATGAGATTCATGATGAGATTTGAGGATGGGGAGGTTGTTGAGCAAAGTGGCACAGTTGTTGGCATTGAAGATTATGATCACTCGACTTGGCCCAACTCTGAGTGGAGATGCTTAAAGGTTCAATGGGTTGAACATTCCTCAATCTCATGCCTAACTAGAGTCTCACCATGGGAATTGGAGCCATTTTGA

>LcARF1D

ATGGCGGGGGACTTGGACACTGATGTTGCTCTCTACAGGGAACTGTGGCATGCCTGCGCTGGACCTCTCGCGAGCGTCCCTCGTGCAGGAGAGCATGTTTATTACTTTCCACAGGGGCACTTGGAGCAGCTGCAAGCATTGACGCATGACTGGACGGAGCAGCGTGTGCATTATTTTGAGTTGTCATCTAAAATTCTCTGTAGAGTAGTTAGTGTTCAGCTTCGGGCTGAACCTGAAACAGAAGATGTTTATGCACAAATAACTTTACTACCCGCACCTGTTCCTAGTGAGGATGCAAGGCAAGATCCTCCTGCTCCAGAACCTAGGTGTACTGTTCTGTCTCTATGCAGGACGCTTGCTGCATCTGATGTGGGAACCAATGGCGTTTTCTCCATTTCTGGGATGGATGCAGATGTTTGCCTACCCCCTCTGGATATGTCCCAGCAACCTCCATGTCAGGAATTGGTTGCGATGGATCTGCACGGGAATGAATGGCATTTTCAGCATATTTTTGAAGATGAACCGCGGTGCCACTTACTCACTGCTGGATGGAGTGAGTACGTTATTTCCAAAAAATTAGTAGCAGGTGATGCATTCATACTCCAGAGGCAAGTCCAGGGAGAAAATGGGAAGCTGCATGTTGGAGTAAGAAGGCATGTGGAAAAAAGTAGACATTCTTCGATTATATCTAATCAAAGTATGTGGCTTGGGTTACTAGCAACTGCTTCTGATGCCATTTTATGTGGAACAATGTTTACTGTCTTCTACAAGCCAAGAACCATCCGGTCTAATTTTATTGTAAATGTTGAAAAGTACCATGAAGCGATGAGATATGATTTCTCTGTGGGGATGAGGTTCAAGATGAGATTTGAGGGTGAGGAAGTTCCAGTACAGAGTTTCATTGGCAAGATTGTCGCTTTTGAAAATTACTGCTCTTCAATGTGGAGCAACTCTAAGTGGAGATCCTTGAAGGTTGTTGTTACTCACATTTTAGGCGGTAATAAGGCGGCTAAAATTTTGGCCAATATGGGAGGGATGAGCCTCCTGCAGCTATCAGGCGAGAGCTCCTTTTCAATAAATGTCACTGAATCCACAACGCTCACCACTTGCCTCACAACTGTTGCAATTGCTGAGTCCAATTCAAGAGGATTGCCATTTCAAAATGAAAATTTGAGCATAATTTAA

>LcARF1A

ATGGCATTCCCAGCGGCAGCTGTGGCGCCTTTGAATCATTCTTCGGGAGGACCCCTTTCAGGGGGCTCCACCGATGCTTTGTACAGGGAATTATGGCATGCTTGTGCTGGACCTCTTGTCTCCCTTCCTCGTGAAGGGGAGCGAGTGTATTACTTCCCTCAAGGGCAACCTAGGCGTCACTTGCTTACTACTGGGTGGAGTGTGTTTGTTAGTTCCAAAAAATTGCTAGCAGGCGATGCATTTATATTTCTGAGAGGAGAAAACGGGGAGCTGCGTGTAGGAGTAAGGAGGCACATGAGGCAGCAGACAAATATGCCATCTTCAGTAATATCTAGTCACAGCATGCATCTTGGGGTACTTGCAACTGCTTCCCATGCCATTGCAACCGGGACACTGTTTTCTATCTTCTACAAGCCAAGGTTCAGTGGCACCATTGTGGGTGTTGAGGATAACAAAACCTCGGCATGGCCCAACTCTGAATGGAGATCCTTGAAGGTTCAATGGGATGAACCTTCATCTATCGTGCGCCCTGAAAGAGTTTCAGCATGGGAATTGGAGCCACTTGTTGCCAATACTACAACTCCTCCTCCTCCAAACTCCCAACCTGCTCAAAGGAACAAGCGGGCAAGGCCACCTGTTTTGCCAACACCAGCCCCAGATCTTTCCGTTCTTGGTATGTGGAAATCCCCGGTTGAATCCCAGGCTTTCTCATATATTGACTCACAGCATGGACGAGACCTCTATCTATCTCCCAAGTTTTCTTCTGCTACGAAGGCCAACCCTCTTGGTTTTGGTGGGAATAGTTCACTAGCTACTGTTACTGGCAACTCAATGTACTGGCCTAATCGAGGAGAAAATGTAATGGAATCATTTGCACCTGTTGTCAGTAAAGAATCCAGTGAAAAGAGACAGGGAACTGGGAACACCTACAAGCTTTTTGGCATCCAGCTAGTTGACAATTCCAATATAGAAGAATCTTTAGCAGCTGTTACCATGTCTGCTACAGGGGGGGATGATCGACCAGTTCCATCCTTGGATGCAGATTCCGAGCAGCATTCGGAACCGTCAAATATTCCTTCTGTAAGTTGTGATGCTGAGAAGTCATGCTTGAGATCTCCTCAAGAGTCGCAAAGCAGACAAATTCGTAGTTGTACAAAGGTTCATATGCAAGGAATTGCCGTTGGTAGGGCTGTCGATTTGACTCGGTTTGATTGTTATGACGATCTTCTCAAAAGATTGGAAGAGATGTTTGACATCGAGGGAGAGCTCTCTGGAGCCACAGAGAAATGGCAAGTTGTTTACACTGATCATGAAGATGACATGATGATGGTTGGGGATGATCCATGGCATGAGTTCTGTAGCATAGTGGGGAAAATTTTCATTTACACAGCCGAAGAAGTGAAGAGGCTGTCACCCAAGATAAAACTTCCATCTAATGAAGAGGTCAAACCAGCCAAGCCTGATGTTGACACAGCTGTGAACACTGAAGATCGGTTGTCAGTTGTTGGACATGGATGCTGA

>LcARF23A

ATGGCTGGTGCGGGTGGAAATGGGAAAGGCCGTGCTGGTGGGGGTGATCAAAAAGGAAAAGCCCCAAGTACTGATCCTCATGTGCATGATGATCAGAGCAGAAAACGTACCAACATTAACGATGCGTTGCCTACCAACAACACTGACCAATGTAGGCAAGATGGGCTATACAAAGCACTATGGCGTGCGTGTGCCGGTCCACGTGTTTATGTTCCATGCGTTGGAGATTACGTCTACTACTTCCTTCAAGGTCACTTGGAACAGATTGAGGCATATGCAAACCATGAAATTAGAGCGGAAATGCCTGTTTATAATGTACCTTCTAAGGTTCTATGTGAGGTTGTCCATGTTCAACTAATGGCGGAAACTGGTACTGATGAGGTATTTGCACAGATTACTTTGCAACCGAAAACAAAGCAAGACATGCAAAATTTGGAAGATGGGAGTTCTGCACCTTTGCCACCAAAAACAAAGGTGTGCTCCTTTAGCAAGAAACTCACTCAATCTGACACCAGCACACATGGTGGATTCTCTGTTCCCAAACGAAAAGCCGATGAGTGTCTACCCCAACTGGACATGTCCAAGGAACCCCCAGCTCAGGAACTGATTGCAACTGATTTGCATGGATATGAATGGCGTTTTCGGCATATATTTCGTGGGACCACGTTTTCCGTCTACTACCATCCTTGGACCAGCCCTTCTGAATTTATCATACCTGTTGATCAATGCGATAAGTTGACTGCCATTGACTATTCTGTTGGGACAAGAATCAGAATGGCGTTTGAGACTGAAGACTTTACGGAAAAGAGAATTGCTGGCACGGTTGTTGGTATCGAAGATGTTGATCGAATTAGGTGGCCTGGTTCAGAGTGGAGATGCCTCAGGGTGAAATGGGATGCCTTATCTGATAGAATAAGGTGTCCAGAAAGAGTCTCTCCATGGAATGTTGAACTGATAGAAGGTGCCAACAGGAAGCCTGCTTCATTTCTGCATCGACAAAAGAGGCCACGTCCAGATAATTTGTCATTTCCTGGGTGTAGCAATGGGGTCTTGCAAGGTCAAAAAAACAATGTCACAGGTGTGAAGGAATCGGGTCCTCTTGAACCGCTGTCATTTCCATATTTGGTTCCCCAAAAGAGTTCTGATTGGGGCCATGTTTTAATGCATGATCCATTTTATCCATGTCACAATGGTAGGGTGTCAATTGCAAGTGGAATCATAGTGAGGAGTTCAGGTCTTCCTAATTACTGGCCTGCGACATATGCAACAAATAGTGTTCGTAACAATGCTGTGTTAAGCAGAAATATTTCAGCTCCCAGTGGCAACTCTCTTAATTCGAGCTCTAAGGAGCGGAGATCTTTGGAATCGAAGAATGAAAATGAATCTCCTCTCTCCCAGTCTCATGGTGGCAAAAAATACATGCTTTTTGGAGTCAATTTAGCCAATACTAGTCCACCGGAGCTCCCTTTACTTCAACAAGCCACGTCCAATGAGCTCGAAAGTCGTTGTTCAGGCCCTCCAATGTCTCAGTCCAGCATTTCAGGACCTTTTCAGGTTTCAGAGCCATCTAATAGAGTATCTGACATCCCTTCACCAAAACAATGCAAGAATTGCCGTCTTCTCAGGAGTTGCACCAAGGTGATCAAGTATGGAACTGCTCTTGGAAGGTCAGTTGACCTCTCGCGATTTGACGGGTATGAAGAACTCGTTTATGAGCTTGACCAGATGTTTGACTTTAATGGAACTTTGATTGATGGGAGCAGCGGGTGGGAGATCACCTTCATCGATGATGAAGGCGACATGTTGCTAACTGGAGACCACCCCTGGAGGGAATTCCTTTGCGCCGTGCGAAGAATGTTCATCTGTCCAAAGGAAGAAATTGACAAACTGAATCCAAGCTCACCAAATCCAACTTGTTCGTTATTTGTGACAATCACGACAACCCTACCAAGTTCAAGCTTGAAAATCCGTGATCCTCCTCCACTCGATGTCCCAGTCTCGTCTCTCCCAATGTCGCTTCTCAAAAACCGACCTTTACATTTACCCACTATTACAAGACGTCAATTCTCTTCAAATTCAACAACCAATGACAAGGAACCACCCCCAGAAGACACGGTTGAGGATCTGAGCGTCCCAGAACATTGGCTGCTCCCTTCCACGGCTTTTGAGGAGTCAGAGTGGCTCAGGGTTACTCTTCACAAGTGGTTAGATGATGAGTACTGCCCGGAGCCCACCAATGTGGAGATCAGCAAGGTTGCTGCCCGCTCCTACTATGAATCATTGCTAGAGAAGCAGAGAGATCTGGGTGAGATTTTACTGAAGATGGCTAGAGCATTGGAGTCTATCTCTTATCAGGATAGCTTTCATGGGGCATTCTCGTCAGCAAATGCAGCAGTCAACTTGATTGTCCAACGCATGGGGCAGCCATAG

>LcARF23B

ATGGCTGGTGTGGGTGGAAATGGGAAAGGCCGTGTTGGTGGGGGTGATCAAAAAGGAAAAGCCCCAATAACTGATCCTCATGTGCATGATGATCAGAGCAGACAACGTACCAACATTAACGATGCGTTGCCTACCAACAACACTGACCAATGTAGGCAAGATGGGCTATACAAAGCACTATGGCGTGCGTGTGCCGGTCCACGTGTTTATGTTCCATGCGTTGGAGATTACGTCGACTACTTCCTTCAAGGTCACTTGGAACAGATTGAGGCATATGCAAACCATGAAATTAGAGCGGAAATGCCTGTTTATAATGTACCTTCTAAGGTTCTATGTGAGGTTGTCCATGTTCAACTAATGGCAGAAACTGGTACTGATGAGGTTTTTGCACAGATTACTTTGCAACCGAAAACAAAGCAAGACATGCAAAATTTGAAAGATGGGAATTCTGCGCCTTTGCCACCAAAAACAAAGGTGTGCTCCTTTAGCAAGAAACTCACTCAATCTGACACCAGCACACACGGTGGATGCTCTGTTCCCAAACGAAAAGCCGATGAGTGTCTACCCCAACTGGACATGTCCAAGGAACCCCCAGCTCAGGAACTGATTGCAACTGATTTGCATGGATATGAATGGCGTTTTCGGCATATATTTCGTGGTCAGCCAAAGCGCCATTTGCTTACTAGTGGTTGGAGTATATTTGTGGCATCAAAAATGCTTAACAGTGGAGATTTATGTATCTTTCTCAGAGGAGAACATGGAGAACTTCGCATAGGGATTCGTCGTCTAATGAAAAAACAGAACTGTGCTTCAACAACAATCATATCTAGCCATAGCATGCAGCATGGTATACTTGCCGGTGCCTTCCATGCAATTTCTACAGGGACCACGTTTTCCGTCTACTACCATCCTTGGACCAGCCCTTCTGAATTTATCATACCTGTTGATCAATGCGATAAGTTGACTGCCATTGACTATTCTGTTGGGACAAGAATCAGAATGGCGTTTGAGACTGAAGACTTTACGGAAAAGAGAATTGCTGGCACGGTTGTTGGTATCGAAGATGTTGATAGAATTAGGTGGCCTGGTTCAGAGTGGAGATGCCTCAGGGTGAAATGGGATACCTTATCTGATAGAATAAGGTGTCCAGAAAGAGTCTCTCCATGGAATGTTGAACTGATAGAAGGTGCCAACAGGAAGCCTGCTTCATTTCTGCATCGACAAAAGAGGCCACGTCCAGATAATTTGTCATTTCCTGGGTGTAGCAATGGGGTCTTGCAAGGTCAACAAAACAATGTCACAGGTGTCAAGGAATCGGGTCCTCTTGAACCGCTGTCATTTCCATATTTGGTTCCCCAAAAGAGTTCTGATTGGGGCCATGTTTTAATGCATGATCCATTTTATCCATGTCACAATGGTAGAGTGTCAATTCCAAGTGGAATCATAGTGAGGAGTTCAGGTCTGCCTACTTACTGGCCTGCGACATATGCAACAAATAGTGTTCGTAACAATGCTGTGTTAAGCAGAAATATTTCGGCTCCCAGTGGCAAGTCTCTTAATTCCAGCTCTAAGGAGCGGAGATCTTTGGAATCGAAGAATGAAAATGAATCTCCTCTCTCGCAGTCTCATGGTGGCAAAAAATACATGCTTTTTGGAGTCAATTTAGCCAATACTAGTCCACCGGAGCTCCCTTTACTTCAACAAGCCACTTCCAATGAGCTCGAAAGTCGTTGTTCAGTCCCTCCAATGTCTCAGTCCGGCATTTCAGAAACTTTTCAGGTTTCAGAGCCATCTAATAGTGTATCTGACATCCCTTCAGGAAAACAATGCAAGAATTGCCGTCTTCTCAAGAGTTGCACCAAGGTGATCAAGTATGGAACTGCTCTTGGAAGGTCAGTTGACCTCTCGCGATTTGACGGGTATGAAGAACTCGTTTATGAGCTTGACCAGATGTTTGACTTTAATGGAACTTTGATTGATGGGAGCAGCGGGTGGGAGATAACCTTCATCGATGATGAAGGCGACATGTTGCTAACTGGAGACCACCCCTGGAAGGAATTCCTTTGCGCCGTGCGAAGAATGTTCATCTGTCCAAAGGAAGACATTGACAAACTGAATCCAAGCTCACCAAATCCAACTTCTGTTTGA

>LcARF10A

ATGGAGACAGAGAAGAGCATTGATCCACAGCTGTGGCACGCATGCGCAGGATCCATGGTTCAAATCCCTCCAATGAACTCCACTGTGTTTTACTTCCCACAAGGCCACGCAGAGCACTGTTTTGGCTCTGTGAATTTCCCTTCTTCAACAACACCAATCCCACCTCTCCTTCTCTGCCGTGTCACCTGTCTCAAGTTCCTTGCAGATTCCGAAACCGATGAAGTGTATGCAAAGATAAGGCTGGTTCCATTACCAGCCACTAGTGATGTTGATTTTGAAGACAGCACCAATAGCATGAGTCTGAATTCTGATACTGATTCAGAGAAACCAGCCTCTTTTGCTAAGACTTTAACCCAATCTGATGCCAATAATGGGGGTGGCTTCTCTGTGCCTAGATACTGTGCTGAGACTATATTTCCAAGGTTGGATTACACTGCTGATCCTCCTGTGCAGACTGTGGTGGCTAAAGATGTTCATGGCGAGATTTGGAAGTTTAGGCACATTTATAGAGGCACCCCTAGAAGGCACTTGTTGACTACTGGGTGGAGTACTTTTGTTAATCAAAAGAAACTTGTTGCTGGGGATTCAATTGTGTTCTTGAGAGCCCAAAACGGGGATCTTTGTGTTGGGATAAGGAGGGCTAAGAAGGGAATTGACTCTCCTTCATCGGGTTGGAATTCGAATAGTGGAAGTGGTGTTGGTGTTAGTCCTTATGGAGGGTTTTCGTGTTTCTTGAGAGAAGAAGAGAACAAGATGATGAGAAATGGAGCTATGAGTTTAAATTCCAATGGAAGTTGCAATTCTAGTGGGAATTTGAGGGGGAGTGGAGGAGGAAGCAAAGTGAAGCCTGATGCAGTCGTGGAGGCAGTGGCGCTTGCAGTTGGTGGGCAGCCATTTGAGGTTGTTTATTATCCCCGTGCAAGCACACCGGAGTTCTGCGTCAAGGCATCTGCTGTGAGGGCGGCAATGAGGGTTCATTGGTGTTCTGGACTGAGATTCAAGATGGCTTTCGAAACGGAGGATTCTTCTAGGATAAGTTGGTTCATGGGGACTATATCATCTGTGCAGGTTGCTGATCCCATCCGCTGGCCTAATTCCCCTTGGCGGCTCCTCCAGGTGACGTGGGATGAGCCAGATTTGCTACAAAATGTGAAACGTGTCAGCCCATGGTTGGTTGAACTGGTATCAAACATGCCCGTCCTTCATCTATCACCATTCTCACCACCAAGAAAGAAGTTGCGACTCCCGCAACAGTTAGACTTCCCTCTTGACGGCCAATTTTCAATGCCGTCATTTTCAGGCAACCCCCTTGGGCCCAGCAGCCCCTTGTGTTGTCTTTCTGACAACACTCCTGCAGGCATACAGGGAGCCAGGCATGCTCAATTCGGAACATCTTTATCAGATTTTCATGTTTACAACAAACTGCAGTCAGGGCTGTTTATGTCCAGTTTCCAGCGGTTCAATCCACAATCTAGAATTTCAGACAGCATCATGGCAAACCACACAAATAGTAACGAGAATTTATCTTGCTTGCTGACAGTGGGGAATTCTAGTCAGAATTTGGAGAAATGTAATAACGTGAAAAAGCACCAGTTTGTACTCTTTGGTCAACCAATACTAACCGAGCAGCAGATCTCTCACAGTTGTTCTAGTGAGGCAGTCACACAAGTTCTTGGAAAAAGTTTGTCAGACGAGATTTCAGGCAAAGCGAAAGCATCTTCTGATGGTTCAGGAATCACTCGTGAACTGCAAATTTCACCACAAAAGTCATCCAGTCCTGAATATTTTTGGAACCGTGGTTTACAAACAACTGATCCTGGCCTAGATACTGGTCACTGCAAGGTATTCATGGAGTCAGAAGATGTGGGACGAACTCTTGACCTCTCGGTTTTTGGCTCTTATGAAGAGCTGTACAGGAGGCTGGCCAACATGTTTGGCATAGAAAGATCAGAGATGCTAAACCATGTACTCTATCAAGATGCAGCTGGTGCTGTTAAAAGAATTGGAGATCAACCATTCAGTGACTTCATAAGGAAGGCCAAAAGATTGACAATTCTGATGGGTTCAGGCAGTGACAATGTCGGAAGGACATGGATCACAGGGATGCGGAATGCTGACAATGGTCTAGACACCCCCAATAAGACCGGTCCGCTGAGCATTTTCGCATAG

>LcARF10B

ATGGAGACAGAGAAGAGCATTGATCCACAGCTATGGCACGCATGCGCAGGATCCATGGTTCAAATCCCTCCAATGAACTCCACTGTGTTTTACTTCCCACAAGGCCACGCAGAGCACTGTTTTGGCTCTGTGAATTTCCCTTCTTCAACAACACCAATCCCACCTCTCCTTCTCTGCCGTGTCACCTGTCTCAAGTTCCTTGCAGATTCCGAAACCGATGAAGTGTATGCAAAGATAAGGCTGGTTCCATTACCAGCCACTAGTGATGTTGATTTTGAAGAGAGCACCAATAGCATGAGTCTGAATTCTGATACTGATTCAGAGAAACCAGCCTCTTTTGCTAAGACTTTAACCCAATCTGATGCCAATAATGGGGGTGGCTTCTCTGTGCCTAGATACTGTGCTGAGACTATATTTCCAAGGTTGGATTACACTGCTGATCCTCCTGTGCAGACTGTGGTGGCTAAAGATGTTCATGGCGAGATTTGGAAGTTTAGGCATATTTATAGAGGCACCCCTAGAAGGCATTTGTTGACTACTGGGTGGAGTACTTTTGTTAATCAAAAGAAACTTGTTGCTGGGGATTCAATTGTGTTCTTGAGAGCCCAAAACGGGGATCTTTGTGTTGGGATAAGGAGGGCTAAGAAGGGAATTGACTCTCCTTCATCGGGTTGGAATTCGAATAGTGGAAGTGGTGTTGGTGTTAGTCCTTATGGAGGGTTTTCGTGTTTCTTGAGAGAAGAAGAGAACAAGATGATGAGAAATGGAGCTATGAGTTTAAATTCCAATGGAAGTTGCAATTCTAGTGGGAATTTGAGGGGGAGTGGAGGAGGAAGCAAAGTGAAGCCTGAGGCAGTCGTGGAGGCAGTGGCGCTTGCAGTTGGTGGGCAGCCATTTGAGGTTGTTTATTATCCCCGTGCAAGCACACCGGAGTTCTGCGTCAAGGAATCCGCTGTGAGGGCGGCAATGAGGGTTCATTGGTGTTCTGGACTGAGATTCAAGATGGCTTTCGAAACGGAGGATTCTTCTAGGATAAGTTGGTTCATGGGGACTATATCATCTGTGCAGGTTGCTGATCCCATCCGCTGGCCTAATTCCCCTTGGCGGCTCCTCCAGGTGACATGGGATGAACCAGATTTGCTACAAAATGTGAAACGTGTCAGCCCATGGTTGGTTGAACTGGTATCAAACATGCCCGTCCTTCATCTATCACCATTCTCACCACCAAGAAAGAAGTTGCGACTCCCGCAACAGTTAGACTTCCCTCTTGACGGCCAATTTTCAATGCCGTCATTTTCAGGCAACCCCCTTGGGCCCAGCAGCCCCTTGTGTTGTCTTTCTGACAACACTCCTGCAGGCATACAGGGAGCCAGGCATGCTCAATTCGGAACATCTTTATCAGATTTTCATGTTTACAACAAACTGCAGTCAGGGCTGTTTATGTCCAGTTTCCAGCGGTTCAATCCACAATCTAGAATTTCAGACAGCATCATGGCAAACCACACAAATAGTAACGAGAATTTATCTTGCTTGCTGACAGTGGGGAATTCTAGTCAGAATTTGGAGAAATGTAATAACGTGAAAAAGCACCAGTTTGTACTCTTTGGTCAACCAATACTAACCGAGCAGCAGATCTCTCATAGCTGTTCCAGTGAGGCAGTCGCACAAGTTCTTGGAAAAAGTTTGTCAGACGAGATTTCAGGCAAAGCGAAAGCATCTTCTGATGGTTCAGGAATCACTCGTGAACTCCAAATTTCACCACAAAAGTCATCCAGTCCTGAATATTTTTGGAACCGTGGTTTACAAACAACTGATCCTGGCCTAGATACTGGTCACTGCAAGGTATTCATGGAGTCAGAAGATGTGGGACGAACTCTTGACCTCTCGGTTTTTGGCTCTTATGAAGAGCTGTACAGGAGGCTGGCCAACATGTTTGGCATAGAAAGATCAGAGATGCTAAACCATGTACTCTATCAAGATGCAGCTGGTGCTGTTAAAAGAATTGGAGATCAACCATTCAGTGACTTCATAAGGAAGGCCAAAAGATTGACAATTCTGATGGGTTCAGGCAGTGACAATGTCGGAAGGATATGGATCACAGGGATGCGGAATGCTGACAATGGTCTAGACACCCCCAGTAAGACCGGTCCACTGAGCATTTTCGCATAG

>LcARF16A

ATGAATAGAGTGATGGATTCAGACAAAAACAGTTCAATCAAGAAGAACTCAACAGAGAGCTGCTTAGATGCACAGCTATGGCATGCCTGTGCTGGTGGCATGGTTCAAATTCCACCAATCAACTCCAAAGTCGTTTACTTTCCTCAAGGCCACTTAGAACATGCCAAAGGAAACAACACTGAGCTCTCAAATCTTCACATCCCTTGCATGATTCCATGTAGAGTTTCTGGTATAAAGTACATGGCAGATACTGAAACTGATGAGGTTTATGCAAAAATCAGGCTGATTTCGTTGAAAAACAATGGATTGGATGATTTTGAGGATGTGGGAAAAGGAGAGTGCGGTGAAAAGCCACCATCTTTTGCAAAGACTTTGACACAGTCTGATGCAAACAATGGTGGTGGCTTCTCAGTTCCACGTTACTGTGCTGAGACTATTTTTCCCCGATTGGATTATAGTGCTGAGCCTCCTGTGCAGACCATTCTTGCTAAGGATGTCCATGGTGAAGTGTGGAAGTTTAGGCATATTTACAGGGGGACTCCTCGTCGTCACCTTTTGACGACAGGGTGGAGCAATTTTGTGAACCAGAAGAAGCTTGTTGCTGGGGATTCAATAGTTTTCTTGAGAGCTGATAATGGGGATCTCTGTGTGGGGATTAGAAGGGCTAAAAAGGGAGGGATTGCTGATGGATCTGATTATTCTTCTGTTGGCTCCTGGAATTCTTCTGGCTTTCCTTTTGCTAGCGGCTATTCTGTTTATGTGAGGGGAGATGATAGTAAGTTGATGAGGAGGAATTCCAATACTGATTTCAGGGCAAATGTCAGCGCTGACTCTGTTGTCCGGGCTGCAACTCTTGCTGCCAATGCCCAACCTTTTGAGGTTGTTTACTATCCCCGTGCCAGTACCCCAGAGTTTGTCGTCAAGGCCTCAGCCGTTAGAGCTGCAATGCAAATTCAGTGGTGTTCTGGGATGAGGTTTAAGATGGCTTTCGAAACTGAGGATTCTTCTCGGATTAGCTGGTTCATGGGAACCATTTCTTCTGTTCAGGTTGCTGATCCCATTCGTTGGCCAAATTCACCTTGGCGAATTCTCCAGGTGGCATGGGATGAGCCAGATCTACTGCAGAATGTGAAGCGTGTTAACCCATGGTTGGTTGAATTAGTATCAAGCATTCCGGCCATCCATCTTTCAACATTCTCACCACCAAGAAAAAGGTTGCGGCTTCCCGAACACCCTGAGTTTCCTCTAATTAACCAAGTTCCAATGCCATCATTTTCCAGGAACCCTGTCATGTTCAGCAGCCCATTAGGTTGCGTTAGGGATAGTACTCCTGCAGGCATACAGGGAGCCAGGCATGCCCACTATGGACCATCTTTATCAGATCTCCAGTTCAACAAGCTGCAGTTGGGTCAGTTTCCGGTTGGTTTTCAGCATCATCATCATCATCCTACTATGGCCCCTAGAATCCCCAGTTGCAATTTCATAAGTGACACTGAAAATAATGACAATATATCCAGCTGGCTTACGATGGGAAATCCCACTCAGAGTTTTAAGGAAAACAATGAAACAAAGACACCCCATATCTTATTGTTTGGTCAGCTCATTCTCCCCCAGCAAAATAGTTCTCAAAGCTGCTCTGGTGATACACTAAGAAACAGTTCTTCGGATGGGAATCTAGAGAAGACAGTGATATCTTCTGACGGCTCTGGTTCTGCAGTACATCAGAATGGCCCATTAGAAAACTCTTCAGATGAAGGGTCTCCTTGGTGCAAAGATCACCAAAAATCTTATATCAGTTTGGAGACTGGCCATTGTAAGGTGTTCATGGAATCAGAAGATGTAGGTCGAACTCTTAATCTGTCGGTTCTTGGATCTTATGAGGAGCTTTATGGAAAGCTAGCCAACATGTTTGGCATAGAAAGTTCGGAGATGTTTAGCAACGTGTCGTATCGTGATGCAGCAGGTTCTACTAAACATACTGGAGATGAGCCCTTCAGTGAGTTTTTGAAGACCGCAAGAAGGCTAACGATTCTTACAGATTCTAGCAGTGACAATGTTGGAAGCTAG

>LcARF16B

ATGAATAGAGTTATGGATTCAGGCAAAAACAGTTCAATCAAGAAGAACTCAACAGAGAACTGCTTAGATGCACAGCTATGGCATGCCTGTGCTGGTGGCATGGTTCAAATTCCACCAGTCAACTCCAAAGTCGTTTACTTTCCTCAAGGCCACTTAGAACATGCCAAAGGGAACAACACTGAGCTCTCAAATCTTCACATCCCTTGCATGATTCCATGTAGAGTTTCTGGTATAAAGTACATGGCAGATACTGAAACTGATGAGGTTTATGCAAAAATCAGGCTGATTTCGTTGAAAAACAATGGATTGGATGATTTTGAGGATGTGGGAAAAGGAGAGTGCGGTGAAAAGCCACCATCTTTTGCAAAGACTTTGACACAGTCTGATGCAAACAATGGTGGTGGCTTCTCAGTTCCACGTTACTGTGCTGAGACTATTTTTCCCCGATTGGATTATAGTGCTGAGCCTCCTGTGCAGACCATTCTTGCTAAGGATGTCCATGGTGAAGTGTGGAAGTTTAGGCATATTTACAGGGGGACTCCTCGTCGTCACCTTTTGACGACAGGGTGGAGCAATTTTGTGAACCAGAAGAAGCTTGTTGCTGGGGATTCAGTAGTTTTCTTGAGAGCTGATAATGGGGATCTCTGTGTGGGGATTAGAAGGGCTAAAAAGGGAGGGATTGGTGATGGATCTGATTATTCTTCTGTTGGCTCCTGGAATTCTTCTGGCTTTCCTTTTGCTAGCGGCTATTCTGTTTATGTGAGGGGAGATGATAGTAAGTTGATGAGGAGGAATTCCAATACTGATTTCAGGGCAAATGTCAGCGCTGACTCTGTTGTCCAGGCTGCAACTCTTGCTGCCAATGCCCAACCTTTCGAGGTTGTTTACTATCCCCGTGCCAGTACCCCAGAGTTTGTCGTCAAGGCCTCAGCCGTTAGAGCTGCAATGCAAATTCAGTGGTGTTCTGGGATGAGGTTTAAGATGGCTTTCGAAACTGAGGATTCTTCTCGGATTAGCTGGTTCATGGGAACCATTTCTTCTGTTCAGGTTGCTGATCCCATTCGTTGGCCAAATTCACCGTGGCGAATTCTCCAGGTGGCATGGGATGAGCCAGATCTACTGCAGAATGTGAAGCGTGTTAACCCATGGTTGGTTGAATTAGTATCAAGCATTCCGGCCATCCATCTTTCAACATTCTCACCACCAAGAAAAAGGTTGCGGCTTCCCGAACACCCAGAGTTTCCTCTAATTAACCAAGTTCCAATGCCGTCATTTTCCAGAAACCCCGTCATGTTCAGCAGCCCATTAGGTTGCGTTAGGGATAGTACTCCTGCAGGCATACAGGGAGCCAGGCATGCCCACTATGGACCATCTTTATCAGATCTCCACTTCAACAAGCTGCAGTCGGGTCAGTTTCCGGTTGGTTTTCAGCATCATCATCATCCTACTATGCCCCCTAGAATCCCCAGTTGCAATTTCATAAGTGACACTGAAAATAATGACAATATATCCAGCTTGCTTACGATGGGAAATACCACTCAGAGTTTTAAGGAAAACAATGAAACAAAGACACCCCATATCTTATTGTTTGGTCAGCTCATTCTCCCCCAGCAAAATAGTTCTCAAAGCTGCTCTGGTGATACACTAAGAAACAGTTCTTCGGATGGGAATCTAGAGAAGACAGTGATATCTTCTGACGGCTCTGGTTCTGCAGTACATCAGAATGGCCCATTAGAAAACTCTTCAGATGAAGGGTCTCCTTGGTGCAAAGATCACCAAAAATCTTATATCAGTTTGGAGACTGGCCATTGTAAGGTGTTCATGGAATCAGAAGATGTAGGTCGAACTCTTAATCTGTCGGTTCTTGGATCTTATGAGGAGCTTTATCGAAAGCTAGCCAACATGTTTGGCATAGAAAGATCGGAGATGTTTAGCAACGTGTCGTATCGTGATGCAGCAGGTTCTACTAAACATACTGGAGATGAGCCCTTCAGTGAGTTTTTGAAGACCGCAAGAAGGCTAACGATTCTTACAGATTCTAGCAGTGACAATGTTGGAAGCTAG

>LcARF16C

ATGGACAAGTGCTTGGATTCTCAACTATGGCACGCCTGCGCTGGAGGGATGGTGCAAATGCCTCCTGTGAACTCCAAAGTCTTCTACTTTCCTCAAGGCCACGCCGAGCACGCTTGTGGCCCTGTAGATTTCAGGAACTGCCCGAGAATTCCACCTTACATTCTCTGTAGAGTCTCAGCTATCAAATTCATGGCTGATCCTGAAACTGATGAGGTTTTTGCCAAAATTAAGTTGGTTCCTATAAGCAACAACGAGCCTGGTTTTGATGATGATGTGATCCTTAATAGGGAGCAAGAGAAGCCGCCTTCTTTTGCCAAGACATTGACTCAATCAGACGCCAACAACGGTGGGGGGTTTTCTGTTCCGAGGTACTGTGCTGAGACGATTTTCCCCAGATTGGATTACTCGGCTGATCCTCCTGTGCAGACCATTTTGGCCAAGGATGTTCATGGGGAGACGTGGAAGTTTAGGCATATTTATAGAGGGACGCCAAGGAGGCATTTGTTGACTACTGGGTGGAGCACTTTTGTGAATCATAAGAAGCTTGTTGCAGGGGATTCGATTGTGTTTTTGAGGGCTGAGAATGGAGATCTTTGTGTTGGAATTCGTCGTGCAAAAAGAGGGATTGGGGGTGGCCCGGAGGTTTCTTCAGGTTGGAATGGGAATTATGTGTCGCCTTATGGGGGCTTTACGGCCTTTTTGAGGGAGGATGAGGGCAAGTTGATGAGAAATGGCAATGGATCGAGCTCCCATAGTGGGAATGGTGGTTTGATGGGGAAGGGAAAAGTGAGGCCTGAATCGGTTATTGAAGCCGCAACGCTTGCAGCGAATAGACAACCGTTTGAGGTTGTGTACTACCCTCGAGCTAGTACTCCGGAGTTCTGTGTTAAGGCATCCTTGGTGAAAGCAGCATTGCAGATCCGGTGGTGTTCCGGGATGAGATTTAAGATGGCTTTTGAAACAGAGGATTCTTCGCGGATTAGTTGGTTTATGGGAACTATATCTTCTGTTCTGGTTTCTGATGCCCTCTATTGGCCTGATTCGCCTTGGAGGCTTCTTCAGGTTACGTGGGATGAACCGGATTTGCTGCAAAATGTGAAACGTGTTAGCCCGTGGTTGGTGGAATTGGTATCAAACATGCCTGCCTTTCATCTATCTACCTTTTCGCCGCCTAGGAAGAAGACAAGATTGCCACAACACCCTGATTTCCCCCTTGATGGCCAACTTCCAATGCCAACATTTCCCGGCAACCTCCTTGGGCACAATAGCCCCTTTGGGTGTCTACCCGATAACACTCCTGCTGGCATGCAGGGAGCCAGGCATGCTCATTATGGTCTATCTTTATCGGATCACCATCTCAATAAACTGCAGTCAGGTCTGTTTTCGGCTGGTTTCCCGCCGCTTGATCGTGCTGCTCCACCCACGAGGGCCTCCAGTAGCCCAGTCATCCAGAAGCCTAGCATGAGTGAGAATGTTTCTTGCTTGCTAACCATGGCGCATTCTACACAGACTTCCAAAAAGCCTGATAATGCAAAGACACCCCAGCTTGTACTTTTTGGCCAACCGATACTTACAGAGCAGCAGATCTCTCTCAGCTGCTCCGGTGATACAGTTTCACCTGTTCTTACAGGAAATAGTTCATCAGAAGGAAATTTAGATAAGATGGCAAACTTTTCTGACAATTCTGGATCTGCAGTTCATCAACAAGGCCTATCTGAGCGCTCATTCTGCGAAGGGCTCCAATGGTACAGGGACAATCGCCAAGAAACAGACAACTTGGAGACTGGTCATTGTAAAGTGTTCATGGAATCCGAGGATGTAGGTCGCACTCTAAACCTTTCACTTCTTGGTTCTTATGATGAATTGTACAAAAAGCTTGCGGACATGTTTGGCATAGAGAATTCTGAGACACTGAGCCATGTACTCTATCGTGATGTTACAGGTGCGGTCAAGCATATTGGAGATGAACCATTCAGTGACTTCATGAAAACAGCTAGAAGGTTGACAATTCTAATGGATTCAAGCAGTGACAATGTAGGAATGTAG

>LcARF16D

ATGGACAAGTGCTTGGATTCTCAACTATGGCACGCCTGCGCTGGAGGGATGGTGCAAATGCCTCCTGTGAACTCCAAAGTCTTCTACTTTCCTCAAGGCCACGCCGAGCACGCTTGTGGCCCTGTAGATTTCAGGAACTGCCCGAGAATTCCACCTTACATTCTCTGTAGAGTCTCAGCTATCAAATTCATGGCTGATCCTGAAACTGATGAGGTTTTTGCTAAAATTAAGTTGGTTCCTATAAGCAACAACGAGCCTGGTTTTGATGATGATGTGATCCTTAACAGGGAGCAAGAGAAGCCGCCTTCTTTTGCCAAGACATTGACTCAATCAGACGCCAACAATGGTGGGGGGTTTTCTGTTCCGAGGTACTGTGCTGAGACGATTTTCCCCAGATTGGATTACTCGGCTGATCCTCCTGTGCAGACCATTTTGGCCAAGGATGTTCATGGGGAGACGTGGAAGTTTAGGCATATTTATAGAGGGACGCCAAGGAGGCATTTGTTGACTACTGGGTGGAGCACTTTTGTGAATCATAAGAAGCTTGTTGCAGGGGATTCGATTGTGTTTTTGAGGGCTGAGAATGGAGATCTTTGTGTTGGAATTCGTCGTGCAAAAAGAGGGATTGGGGGTGGACCGGAGGTTTCTTCAGGTTGGAATGGGAATTGTGTGTCGCCTTATGGGGGCTTTACGGCCTTTTTGAGGGAGGATGAGGGCAAGTTGATGAGAAATGGCAATGGAAATGTTAATGGATCGAGCTCCCATAGTGGGAATGGTGGTTTGATGGGGAAGGGAAAAGTGAGGCCTGAATCGGTTATTGAAGCTGCAACGCTTGCAGCGAATAGACAACCGTTTGAGGTTGTGTACTACCCTCGAGCTAGTACTCCGGAGTTCTGTGTTAAGGCATCCTTGGTGAAAGCAGCATTGCAGATCCGGTGGTGTTCCGGGATGAGATTTAAGATGGCTTTTGAAACAGAGGATTCTTCGCGGATTAGTTGGTTTATGGGAACTATATCTTCTGTTCTGGTTTCTGATGCCCTCTATTGGCCTGATTCGCCTTGGAGGCTTCTTCAGGTTACGTGGGATGAACCGGATTTGCTGCAAAATGTGAAACGTGTTAACCCGTGGTTGGTGGAATTGGTATCAAACATGCCTGCCTTTCATCTGTCTACCTTTTCGCCGCCTAGGAAGAAGACAAGATTGCCACAACACCCTGATTTCCCCCTTGATGGCCAACTTCCAATGCCAACATTTCCCGGCAACCTCCTTGGGCACAATAGCCCCTTTGGGTGTCTACCCGATAACACTCCTGCTGGCATGCAGGGAGCCAGGCATGCTCATTATGGTCTATCTTTATCGGATCACCATCTCAATAAACTGCAGTCAGGTCTGTTTTCGGCTGGTTTCCCGCCGCTTGATCGTGCTGCTCCACCCATGAGGGCCTCCAGTAGCCCAGTCATCCAGAAGCCTAGCATGAGTGAGAATGTTTCTTGCTTGCTAACCATGGCGCATTCTACACAGACTTCTAAAAAGCCTGATAATGCAAAGACACCCCAGCTTGTACTTTTTGGCCAACCGATACTTACAGAGCAACAGATCTCTCTCAGCTGCTCCGGTGATACAGTTTCACCTGTTCTTACAGGAAATAGTTCATCAGAAGGAAATTTAGATAAGATGGCAAACTTTTCTGACAATTCCGGATCTGCAGTTCATCAACAAGGCCTATCTGAGCGCTCATTCTGCGAAGGGCTCCAATGGTACAGGGACAATCGCCAAGAAACAGACAACTTGGAGACTGGTCATTGTAAAGTGTTCATGGAATCCGAGGATGTAGGTCGCACTCTAAACCTTTCACTTCTTGGTTCTTATGATGAATTGTACAAAAAGCTTGCGGACATGTTTGGCATAGAGAATTCTGAGACACTGAGCCATGTACTCTATCGTGATGTTACAGGTGCGGTCAAGCATATTGGAGATGAACCATTCAGTGACTTCATGAAAACAGCTAGAAGGTTGACAATTCTAATGGATTCAAGCAGTGACAATGTAGGAATGTAG

>LcARF17A

ATGCCTCAGCAACGACGCCCCTCTCCCTCCCAGCCTAGCCGGGTCGACCCGGCCGTCTGGCGCGCTTGCGCCGGTTCTCTTGTTCAAATCCCAACCGTCCATTCTAGGGTTTACTACTTCCCCCAAGGCCACCTGGAGCGATCCTCTCCCTCCCCTACCGTCCTCTCCTCTCTCAACATCTCCAGTGCCGTCATCCAATGCGTCGTCTCACAACTTCACTTCCTCGCCGACCCCGTCACCGACCAAGTCTTCGCCAAAGTGCTTCTCACGCCTGTCAACACTCTAGAGCCTTCCAGAGAGCAGCAGCAACCACAGCACCAAATGGAGGACAGGAATGTCGAGAACTATGTTCACGAGAATGTCGTGGCTCCGTTTGCTAAGATTTTAACGCCGTCAGATGCTAATAACGGCGGCGGCTTCTCCGTCCCGAGATTCTGCGCCGATTCGATCTTCCCGCCGCTGAACTACCAGGCGGATCCTCCCGTGCAAACCCTATCCATCACCGACATCCACGGCCACGTTTGGGATTTCCGCCACATTTACCGGGGGACGCCGCGGCGCCACCTCTTGACCACCGGCTGGAGCAAGTTCGTCAACCGCAAGAAACTGATCGCTGGTGATTCCGTCGTTTTCATGAGAGATTCGATGGGGATGCTGTTCGTCGGAATCCGGCGGGCAGAGAGGTATGGGAGCAGTGGAGGAGACGGTGCGCGGTGGGGGGAGCATTCGGGAGTCAAGGTGGAGGCGTTCACGGAGGCGCTGGAGAGGGCGTCGCTGGGGCTCCCTTTCGAGGTGGTGTATTATCCTAGGACTGGATGGCCGGATTTTATAGTAAGGGCTGATGTTGTGGAGGCCGCCATGAAGATCTTTTGGACAGCTGGCGTGAGAGTGAAGATGGTGACAGAGACGGAGGATTCCTCAAGGGTCACGTGGTTTCAGGGGACCGTCAGCGATGCTTCTATGCCCACTACCGGGCCTTGGTGTGGCTCTCCATGGCGGATGCTTGAGGTTACATGGGATGAACCTGAAGTTCTGCTGAACGTGAAGAGAGTAAGCCCTTGGCAACTTGAGTTTGTGTCATCCTCCCTACCACTTCATACCCCTTTCCCCTCAGCAAAGAGATTGAAATTTCCTCAGAATTCTGGGTTGGTGACTGATGGAGAGGAGGACATCTTCTCTCCGCTGAGAGGATTAACTAATTCAACAATGGGGCAGATGAACCCTTCATCGTTGAATTGTAATACTTTTCCTGTTGGCATGCAGGGAGCCAGGCAAAATACTAATAGCATTTTTAGTTTATCAAATTTCATAAGTGAGAATACTCATCAGACATGCACTGAGAATACCACTGGCATCAACATTGTGCCAAAGTTGAAAAATGTGTGCACTGAGTTGAACATGGGCAGTTCACAGTCTGAGAACTTGTCACCTGATAGCCATAGTAGTGCATACTCCTTTGGCACTGAACTTGGAAATCAGGGATGCAACTCCTCTAAAATTGGGGCTAGTTCATTTCAATTGTTTGGTAAGATCATACATATGAATCAGCCTGTTGAAAGTGGTTTTGATGATGTTGCTTGTGTAGAAGATGATGGTGGCAAGGAGTTCAATGAAACTGAAGGTGTGAACAAGCCAACAGATCTTTCCTTGACTTACACGGCACTGCTTAACAGGTTTGGTGTTCAATGCCAAGGAGAATCAGCAGATGAAGAAACTTGCTCTTTATGA

>LcARF17B

ATGCCTCAGCAACGACGCCCCTCTCCCTCCCAGCCTAGCCGGGTCGACCCGGCCGTCTGGCGCGCTTGCGCCGGTTCTCTTGTTCAAATCCCAACCGTCCATTCTAGGGTTTACTACTTCCCCCAAGGCCACCTGGAGCGATCCTCTCCCTCCCCTACCGTCCTCTCCTCTCTCAACATCTCCAGTGCCGTCATCCAATGCGTCGTCTCACAACTTCACTTCCTCGCCGACCCCGTCACCGACCAAGTCTTCGCCAAAGTGCTTCTCACGCCTGTCAACACTCTAGAGCATTCCAGAGAGCAGCAGCAACCACGGCACCAAATGGAGGACAGGAATGTCGAGAACTATGTTCACGAGAATGTCGTGGCTCCGTTTGCTAAGGTTTTAACGCCGTCAGATGCTAATAACGGCGGCGGCTTCTCCGTCCCGAGATTCTGCGCCGATCAACCCCCTCTCTCTCTCGATCCTCCCGTGCAAACCCTATCCATCAGCGACATCCACGGCCACGTTTGGGATTTCCGCCACATTTACCGGGGGACGCCTCGGCGCCACCTCTTGACCACCGGCTGGAGCAAGTTCGTCAACCGCAAGAAGCTGGTCGCTGGTGATTCCGTCGTTTTCATGAGAGATTCGATGGGGATGCTGTTCGTCGGAATCCGGCGGGCGGAGAGGTATGGGAGCAGTGGAGGAGACGGTTCGCGGTGGGGGGAGCATTCGGGAGTCAAGGTGGAGGCGGTGACGGAGGCGCTGGAGAGGGCGTCGCTGGGGCTCCCTTTCGAGGTGGTGTATTATCCTAGGACTGGATTGCCGGATTTTATAGTAAGGGCTGATGTTGTGGAGGCCGCCATGAAGATCTTTTGGACAGCTGGCGTGAGAGTGAAGATGGTGACAGAGACGGAGGATTCCTCAAGGGTCACGTGGTTTCAGGGGACCGTTAGCGATGCTTCTATGCCCACTACTGGGCCTTGGTGTGGCTCTCCATGGCGGATGCTTGAGGTTACATGGGATGAACCTGAAATTCTGCTGAACGTGAAGAGAGTAAGCCCTTGGCAACTTGAGTTTGTGTCATCCTCTCTACCACTTCATACCCCTTTCCCCTCAGCAAAGAGATTGAAATTTCCTCAGAATTCTGGGTTGGTGACTGATGGAGAGGAGGACATCTTCTCTCCGCTGAGAGGATTAACTAATTCAACAATGGGGCAGATGAACCCTTCATCGTTGAATTGTAATACTTTTCCTGTTGGCATGCAGGGAGCCAGGCAAAATACTAATAGCATTTTTAGTTTATCAAATTTCATAAGTGAGAATACTCATCAGACATGCACTGAGAATACCACTGGCATCAACATTGTGCCAAAGTTGAAAAATGTGTGCACTGAGTTGAACATGGGCAGTTCACAGTCTGAGAACTTGTCACCTGATAGCCATAGTAGTGCATACTCCTTTGGCACTGAACTTGGAAATCAGGGATGCAACTCCTCTAAAATTGGGGCTAGTTCATTTCAATTGTTTGGTAAGATCATACATATGAATCAGCCTGTTGAAAGTGGTTTTGATGATGTTGCTTGTGTAGAAGATGATGGTGGCAAGGAGTTCAATGAAACTGAAGGTGCGAACAAGCCAACAGATCTTTCCTTGACTTACACGGCACTGCTTAACAGGTTTGGTGTTCAATGCCAAGGAGAATCAGCAGATGAAGAAACTTGCTCTTTATGA
